# Novel Functionalized Amino Acids as Inhibitors of GABA Transporters with Analgesic Activity

**DOI:** 10.1021/acschemneuro.1c00351

**Published:** 2021-08-04

**Authors:** Beata Gryzło, Paula Zaręba, Katarzyna Malawska, Gabriela Mazur, Anna Rapacz, Kamil Ła̧tka, Georg C. Höfner, Gniewomir Latacz, Marek Bajda, Kinga Sałat, Klaus T. Wanner, Barbara Malawska, Katarzyna Kulig

**Affiliations:** † Faculty of Pharmacy, Jagiellonian University Medical College, 9 Medyczna Street, 30-688 Kraków, Poland; ‡ Department of Pharmacy, Center for Drug Research, Ludwig-Maximilians-Universität München Butenandtstraße 5-13, 81377 Munich, Germany

**Keywords:** GABA transporters, mGAT1−4 inhibitors, [^3^H]GABA uptake, neuropathic pain models, antiallodynic activity, antihyperalgesic activity

## Abstract

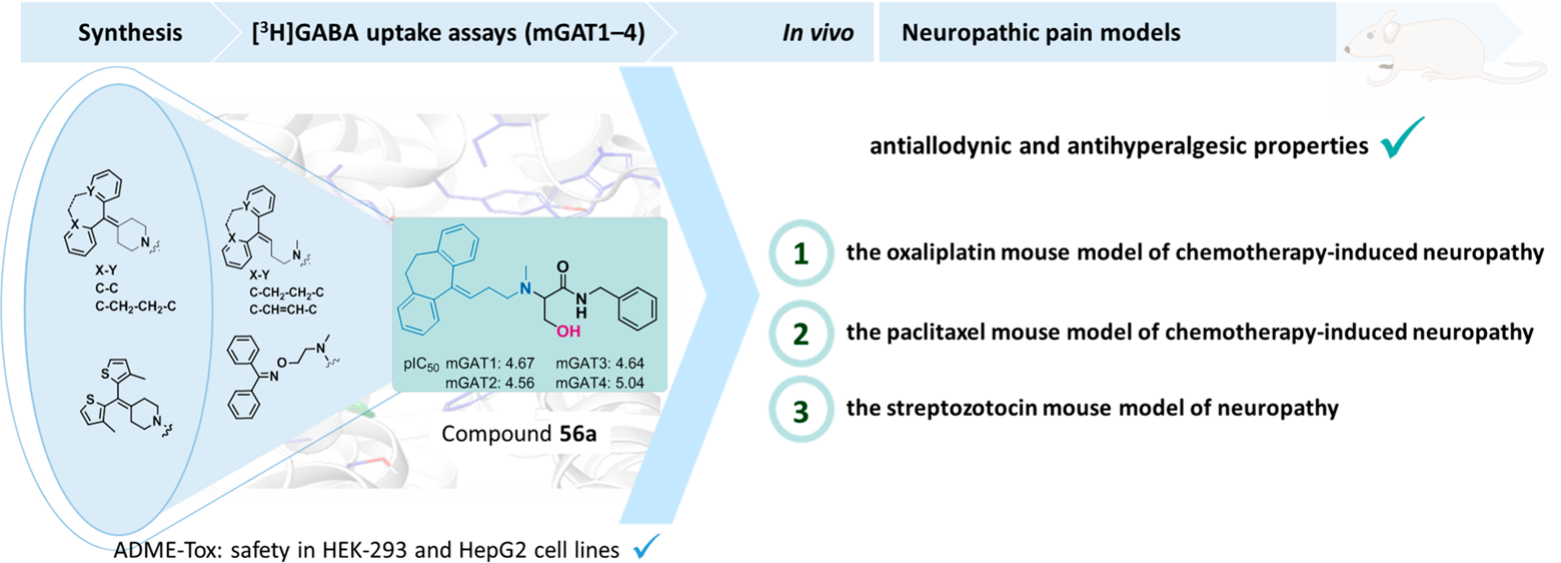

Neuropathic pain resistance to pharmacotherapy has encouraged researchers to develop effective therapies for its treatment. γ-Aminobutyric acid (GABA) transporters 1 and 4 (mGAT1 and mGAT4) have been increasingly recognized as promising drug targets for neuropathic pain (NP) associated with imbalances in inhibitory neurotransmission. In this context, we designed and synthesized new functionalized amino acids as inhibitors of GABA uptake and assessed their activities toward all four mouse GAT subtypes (mGAT1–4). According to the obtained results, compounds 2*RS*,4*RS*-**39c** (pIC_50_ (mGAT4) = 5.36), **50a** (pIC_50_ (mGAT2) = 5.43), and **56a** (with moderate subtype selectivity that favored mGAT4, pIC_50_ (mGAT4) = 5.04) were of particular interest and were therefore evaluated for their cytotoxic and hepatotoxic effects. In a set of *in vivo* experiments, both compounds **50a** and **56a** showed antinociceptive properties in three rodent models of NP, namely, chemotherapy-induced neuropathic pain models (the oxaliplatin model and the paclitaxel model) and the diabetic neuropathic pain model induced by streptozotocin; however compound **56a** demonstrated predominant activity. Since impaired motor coordination is also observed in neuropathic pain conditions, we have pointed out that none of the test compounds induced motor deficits in the rotarod test.

## 1 Introduction

**Figure 1:**
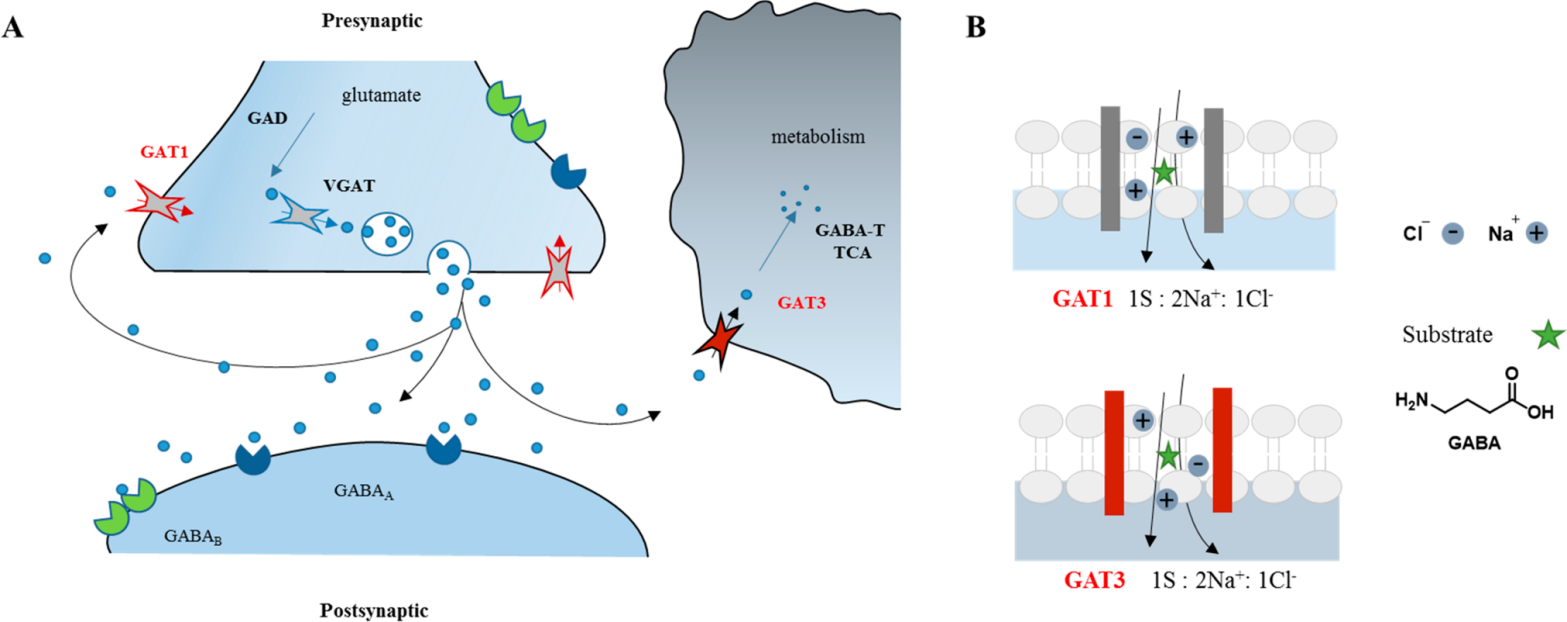
Diagram of GABA transport systems in neurons and glia (A) and stoichiometry between GABA and co-ions in GAT1 and GAT3 (B).

The γ-aminobutyric acid (GABA) is a neurotransmitter known for its inhibitory modulation of neuronal networks.^1,2^ Endogenous GABA is synthesized from glutamate^3,4^ and controls the generation of membrane potential oscillations by acting on two types of receptors, ionotropic (GABA_A_) and metabotropic (GABA_B_) as summarized in Figure 1.^5^ Plasma membrane transporters of GABA (GATs) are components of one of the pathways responsible for terminating inhibitory signaling. GATs expression in different cell types is highly dynamic and can be modified depending on the activity. Reuptake achieved through GATs occurs in nerve terminals (allowing GABA to be recycled as a neurotransmitter) and/or the surrounding glial cells, whereby glial GATs are responsible for 20% reuptake of GABA, Figure 1.^6^

GABA specific transport systems represent a mechanism that regulates the efficiency of GABA transmission; thus, since 1990, the family of GAT sodium symporters has become an interesting biological target. Cloning of several GATs has led to a better understanding of the molecular properties of this solute carrier family, Figure 1. GATs have a confusing numbering system; hence, we present a summary of the current nomenclature based on that from the International Union of Basic and Clinical Pharmacology (IUPHAR). Human (and rat) GAT1 (SLC6A1), GAT2 (SLC6A13), GAT3 (SLC6A11), and BGT1 (SLC6A12)^7^ correspond to mouse mGAT1, mGAT2, mGAT3, and mGAT4, respectively.^8^ In this paper, we present the results of an *in vitro* test on murine GATs, and the mouse nomenclature (mGAT1–GAT4) will be used in a later section.

mGAT1 (representing neuronal uptake) and mGAT4 (mediating transport into glial cells) are mainly localized in the central nervous system (CNS). The peripherally located mGAT2 (BGT1) is largely expressed in the liver, with lower levels observed in the kidneys and at the brain surface in the leptomeninges.^9,10^ mGAT3 is a second GABA transporter distributed mainly in the peripheral tissues, and since there is a lack of selective and potent mGAT3 inhibitors, the function of mGAT3 remains unclear.^9−11^ Due to the diversity in GAT subtype localization and function, researchers have focused on the synthesis of subtype-selective inhibitors.

**Figure 2:**
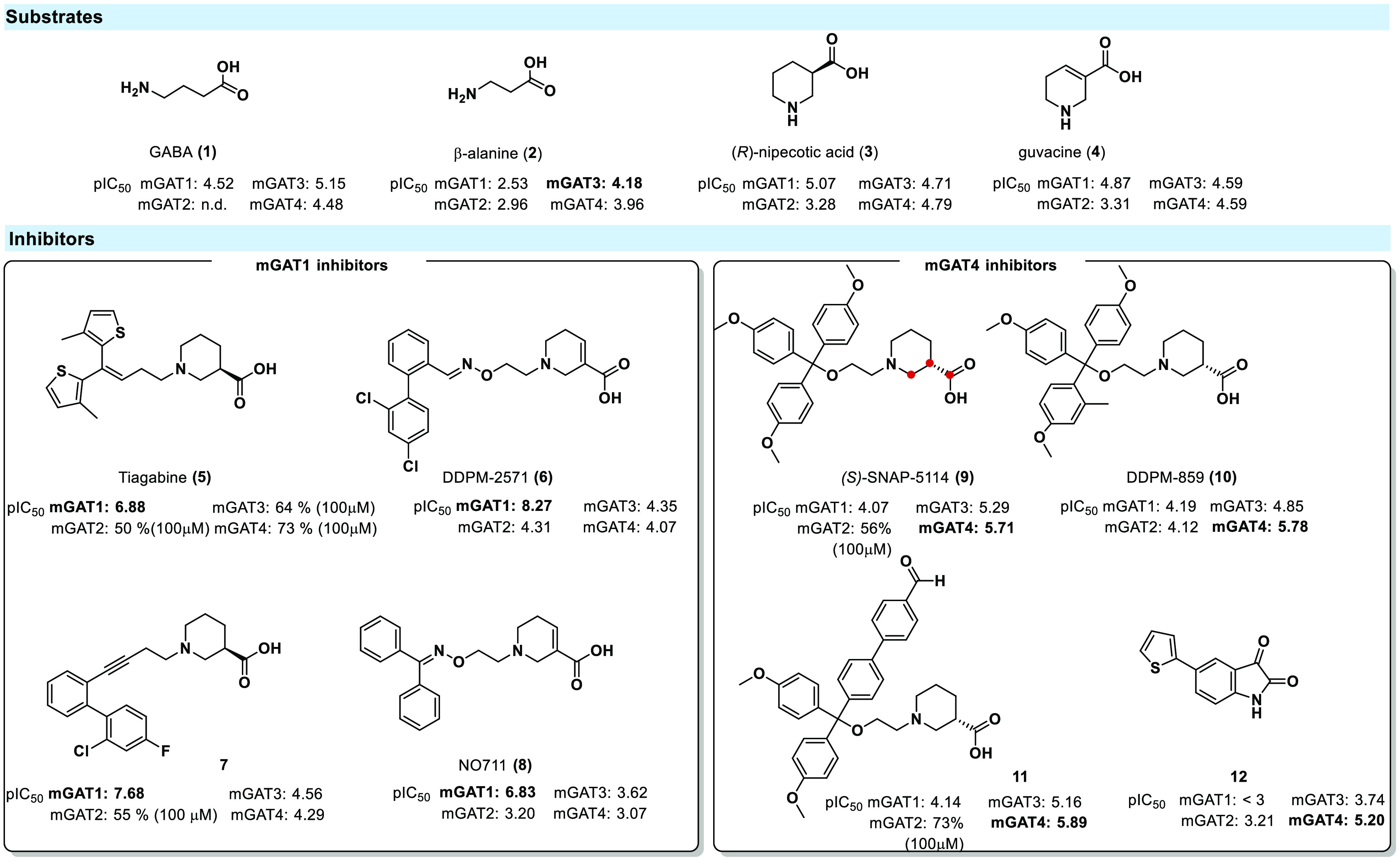
Structures and inhibitory activities (pIC_50_ values) of GAT substrates (**1**,^18^**2**,^18^**3**,^19^**4**^19^) and GAT inhibitors (**5**–**12**) separated according to their transporter subtype selectivity: mGAT1 (tiagabine (**5**),^20^ DDPM-2571 (**6**),^20^**7**,^21^ NO711 (**8**)^19^) and mGAT4 ((*S*)-SNAP-5114 (**9**),^22^ DDPM-859 (**10**),^22^**11**,^23^**12**^24^).

Small amino acids are known to be GAT substrates; moreover, some substrate preferences toward GAT subtypes are well established. All four GATs can transport GABA (**1**). Additionally, β-alanine (**2**) is a substrate for both mGAT3 and mGAT4 with a low affinity for mGAT1 and mGAT2. A functional approach based on small molecules, such as (*R*)-nipecotic acid (**3**) or guvacine (**4**), has resulted in the synthesis of many subtype-selective inhibitors (Figure 2). Effective blockade of the uptake toward GAT is believed to have therapeutic value for not only epileptic seizures but also neuropathic pain (NP) and several abnormalities, including tremors, ataxia, and nervousness.^12^ mGAT1 inhibitors are the most potent compounds, and a wide range of these subtype-selective inhibitors are known (**5**–**8**, Figure 2). One example is the mGAT1 selective compound tiagabine (**5**), which has been approved by the FDA for adjunctive treatment of seizures. Furthermore, tiagabine (**5**) turned out to be highly effective in various rodent neuropathic pain models.^13,14^ The guvacine derivative DDPM-257 (**6**) is another selective mGAT1 inhibitor that is effective in mouse models of seizures, anxiety, depression, and acute and tonic pain.^15^ Moreover, mGAT4 remains also a challenging target, especially since mGAT4 inhibitors seem to be suitable for antinociceptive activity.^16,17^ In this context, the GABA transporters were found to be interesting biological targets in the search for new treatment of NP. Nevertheless, due to the low to moderate subtype selectivity of **9**–**12**, the development of new selective inhibitors remains an important approach for distinguishing non-mGAT1 pharmacology (Figure 2).

**Figure 3:**
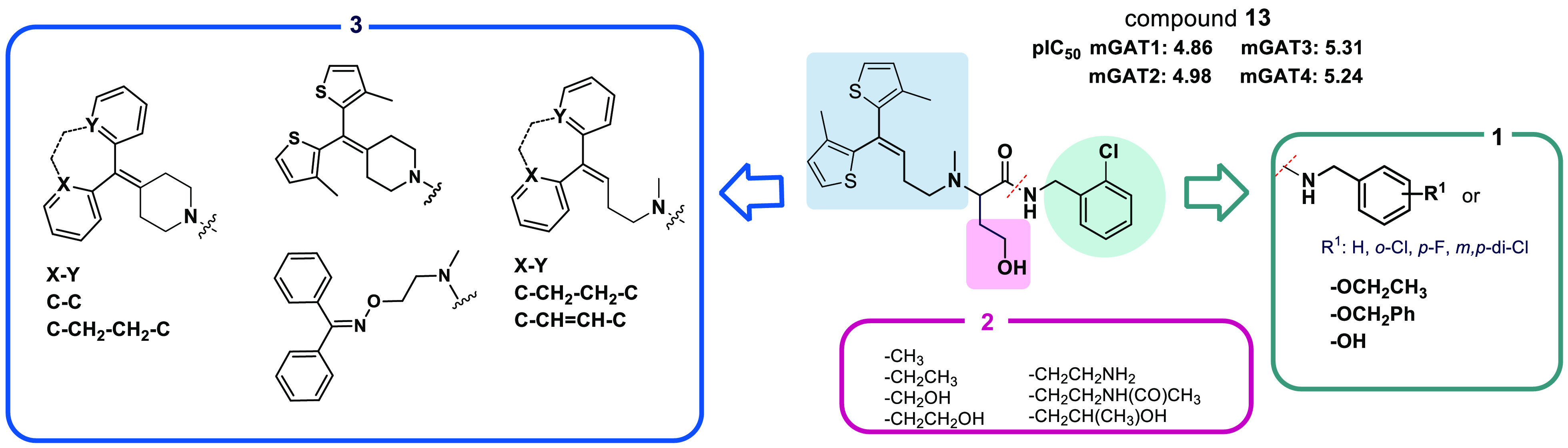
Schematic summary of the structural modification approach from parent compound **13**.^25^

We previously obtained a series of GABA analogs with mGAT3/4 subtype preference with the most interesting compound **13** that could reduce tactile allodynia in neuropathic mice.^25^ In this paper, we present a continuation of our previous work with new derivatives that can be classified as analogs of parent compound **13**. A summary of these modifications is presented in Figure 3. The first purpose of this study was to investigate how stiffening the lipophilic fragment in the second position of the *N*-benzylamide derivatives affects subtype preference and/or mGAT1–4 transporter inhibition compared to the more flexible analogs. Therefore, we introduced a piperidine ring to replace the flexible carbon chain (the lipophilic fragment; blue rectangle, Figure 3). To maintain an analogous structure, bisthiophene, fluorenyl, or suberenone was introduced in the 4-position of the piperidine ring (fragment 3, Figure 3). Moreover, motivated by the inhibitory activity of NO711 (**8**), we decided to introduce an oxime subunit into the 4-position of the 4-hydroxy- and 4-aminobutanamide derivatives. This moiety is interesting for the structure–activity relationship (SAR) discussion due to its potential ability to impact the binding mode of mGAT1 inhibitors.^26^ Second, on the basis of the fact that a large number of mGAT1 ligands possess a carboxylic acid fragment, we previously synthesized propanoic acid ethyl and benzyl ester derivatives for hydrolysis into the corresponding carboxylic analogs of the parent *N*-benzamides (fragment 1, Figure 3). The last structural change was to introduce variation into the length of the main carbon chain. Therefore, the synthesized compounds are 3–5 carbon atoms in length. We exchanged the hydroxyl/amino groups for methyl or isopropyl groups to determine whether the presence of hydrogen bond donors affects GAT inhibitory potency in the present group of compounds. To explore the molecular interactions of novel obtained GABA uptake inhibitors with GABA transporters, computational docking and molecular dynamics studies have been performed. Finally, to confirm the therapeutic potential of the obtained compounds, we tested selected the most potent compounds in *in vitro* assays, for their antiallodynic and antihyperalgesic activities in three rodent models of NP.

## 2 Results and Discussion

### 2.1 Chemistry

To synthesize the target compounds, we used secondary amines **17** (**A**), **20** (**B**), **21** (**C**), **25** (**D**), **26** (**E**), **32** (**F**), and **33** (**G**). Amines were prepared according to the synthetic route presented in Scheme S1 (see Supporting Information).^28−31,33−40^

#### 2.1.1 Synthesis of the 4-Hydroxypentanamide (**37a**–**c**, **39a**–**c**, **41**, **43a**,**b**, **45**), 4-Hydroxybutanamide (**47a**–**c**), 4-Aminobutanamide (**50a**,**b**), and 4-Acetamidobutanamide (**51a**,**b**) Derivatives

**Scheme 1:**
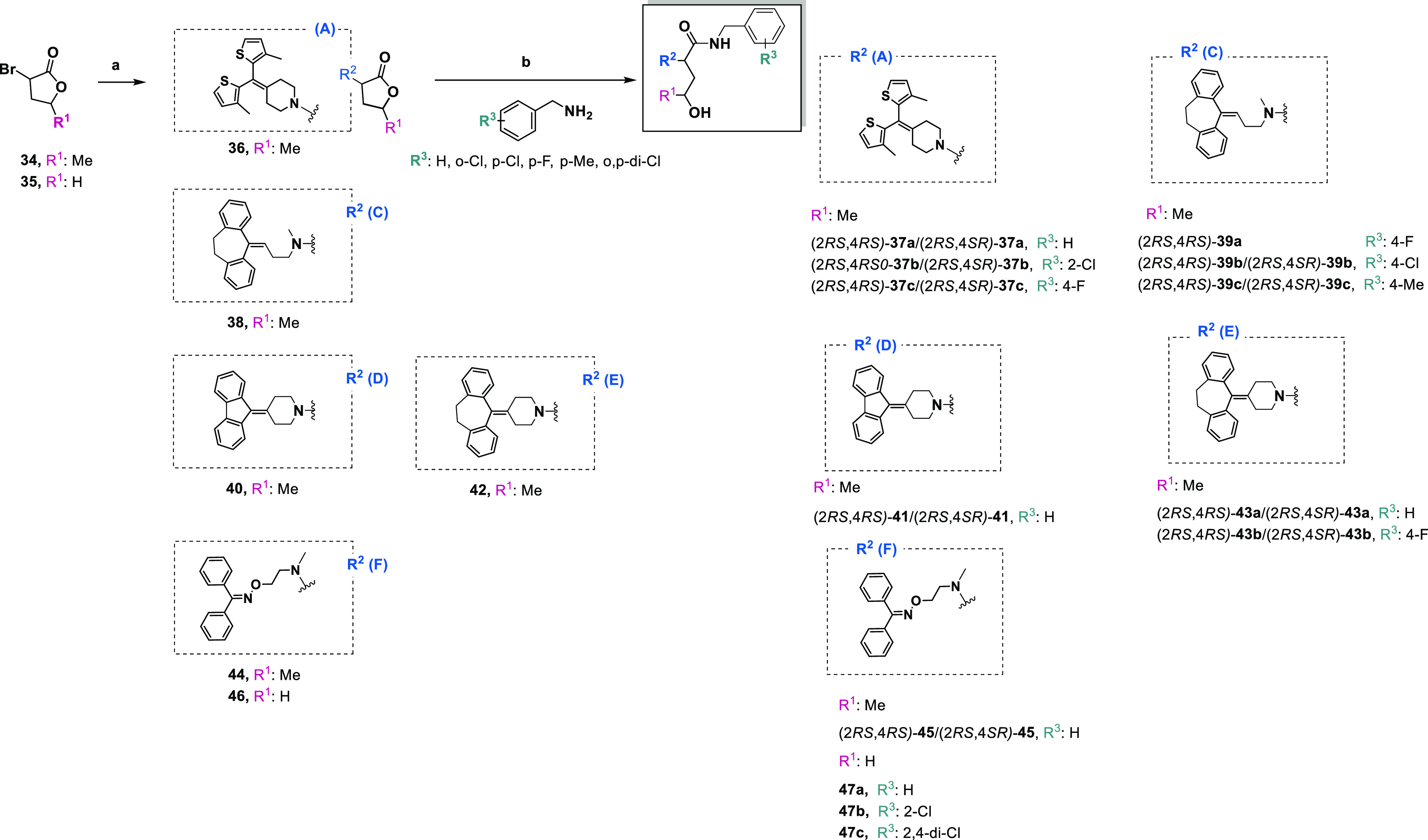
Synthesis of the 4-Hydroxypentanamide (**37a**–**c**, **39a**–**c**, **41**, **43a**,**b**, **45**) and 4-Hydroxybutanamide (**47a**–**c**) Derivatives Reagents and conditions: (a) suitable amine (**17** (**A**), **21** (**C**), **25** (**D**), **26** (**E**), or **32** (**F**)), TBAB, K_2_CO_3_, CH_3_CN, 15 min at 0 °C and 16 h at rt; (b) argon, dry THF, reflux, 48 h.

The designed target 4-hydroxypentanamide (**37a**–**c**, **39a**–**c**, **41**, **43a**,**b**, **45**) and 4-hydroxybutanamide (**47a**–**c**) derivatives were obtained in the two main steps. First, nucleophilic substitution between building blocks **17** (**A**), **21** (**C**), **25** (**D**), **26** (**E**), or **32** (**F**) and 3-bromo-5-methyldihydrofuran-2(3*H*)-one (**34**) or 3-bromodihydrofuran-2(3*H*)-one (**35**) was performed to obtain compounds **36**, **38**, **40**, **42**, **44**, and **46** (Scheme 1).^41^ Then, aminolysis was conducted following previously described synthetic procedures.^25,41−44^ Compounds **37a**–**c**, **39a**–**c**, **41**, **43a**,**b**, and **45** were isolated in pure form as a mixture of racemic diastereomers, e.g., 2*RS*,4*RS*-**45** and 2*RS*,4*SR*-**45**, with a diastereoselectivity (ds) of approximately 7:3 (see the experimental section).

**Scheme 2:**
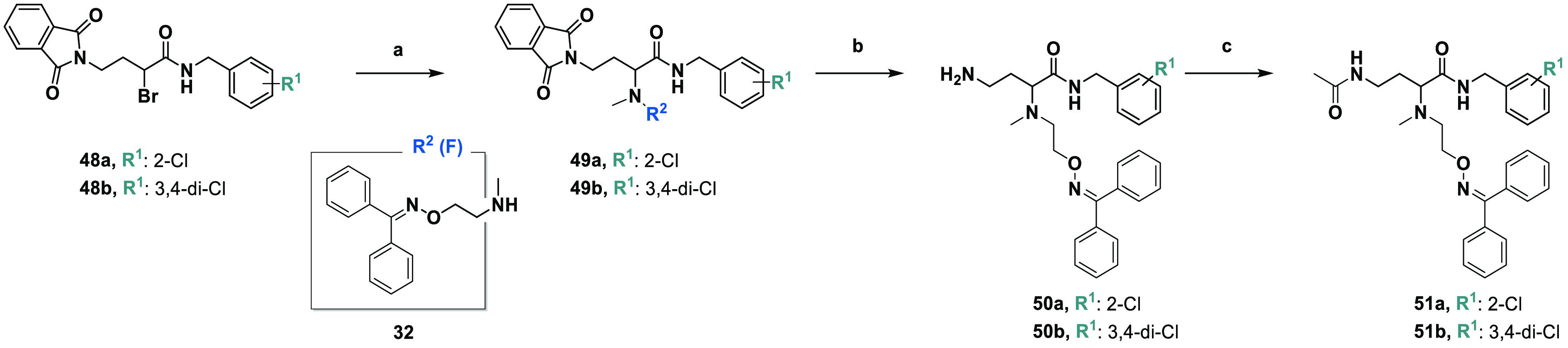
Synthesis of the 4-Aminobutanamide (**50a**,**b**) and 4-Acetamidobutanamide (**51a**,**b**) Derivatives Reagents and conditions: (a) KI, K_2_CO_3_, CH_3_CN, reflux, 24 h; (b) NH_2_NH_2_ (50–60%), EtOH, 2 h at 60 °C and 5 h at rt; (c) DMAP, DCC, CH_3_COOH, DCM, 10 min at 0 °C and 20 h at rt.

The 4-aminobutanamide derivatives (**50a**,**b**) and 4-acetamidobutanamide derivatives (**51a**,**b**) were prepared according to the synthetic route shown in Scheme 2. Compounds **50a** and **50b** were obtained from previously described synthetic procedures^46,47^ via the N-alkylation of **32** (F) with **48a**,**b** and the hydrazinolysis of **49a**,**b**. Acetylation of **50a** and **50b** gave **51a** and **51b**, respectively (Scheme 2).^25,48^

#### 2.1.2 Synthesis of 3-Hydroxypropanamide Derivatives (**54a**–**c**, **55a**–**e**, **56a**–**e**, **57**, **58**, and **59a**–**c**)

**Scheme 3:**
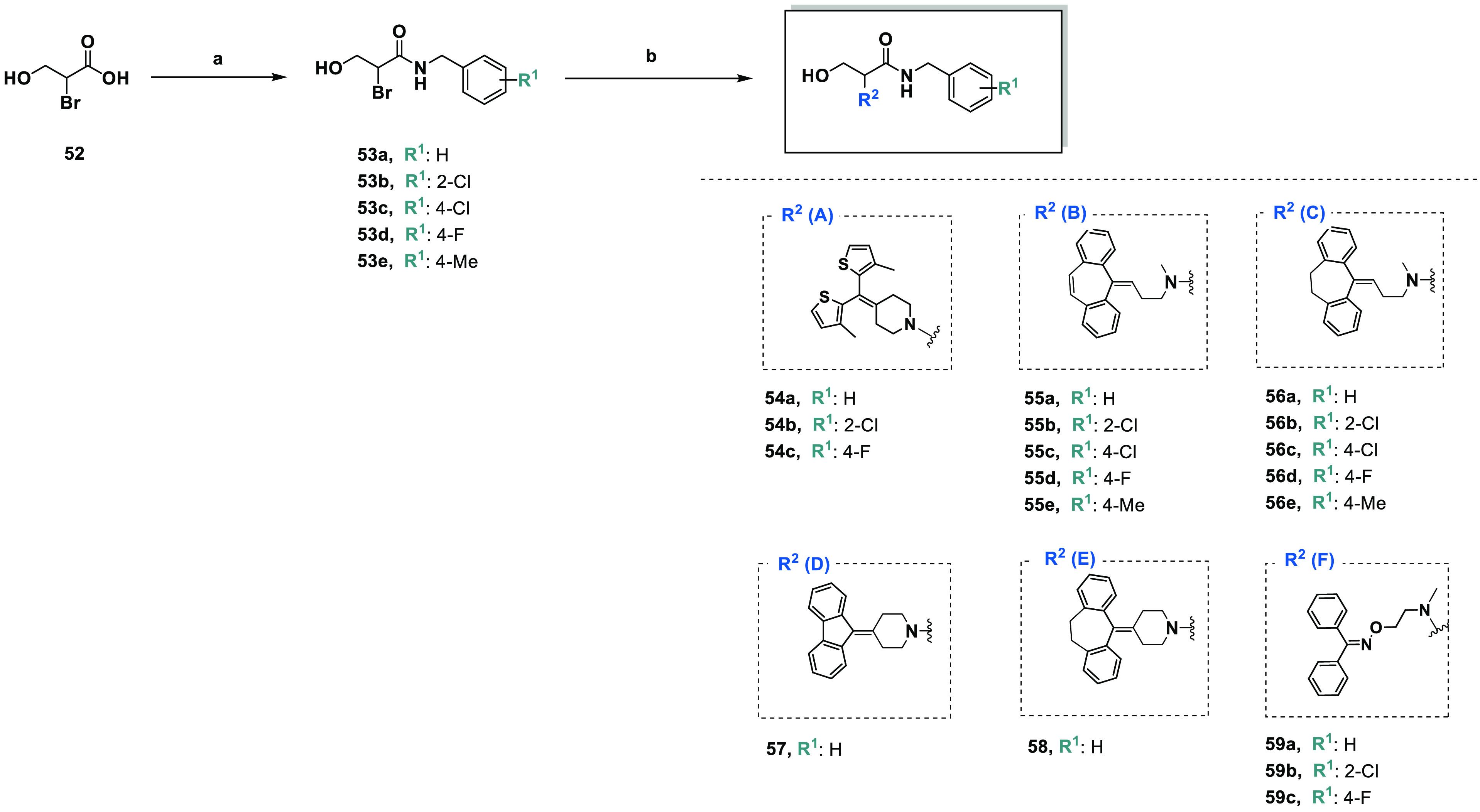
Synthesis of 3-Hydroxypropanamide Derivatives (**54a**–**c**, **55a**–**e**, **56a**–**e**, **57**, **58**, and **59a**–**c**) Reagents and conditions: (a) T3P, TEA, *N*-benzylamine derivative, dry DCM, −17 °C for 30 min and rt for 1 h, argon; (b) suitable amine (**17** (**A**), **20** (**B**), **21** (**C**), **25** (**D**), **26** (**E**), or **32** (**F**)), DIPEA, TBAB, dry DMF, reflux, 12 h.

3-Hydroxypropanamide derivatives were obtained according to a previously reported synthetic route shown in Scheme 3.^25,49^ 2-Bromo-3-hydroxypropanoic acid (**52**) has been obtained from unprotected serine. Designed amides (**53a**–**e**) were formed after activation of acid **52** by *n*-propanephosphoric acid anhydride (T3P).

Alkylation of amine **17** (**A**), **20** (**B**), **21** (**C**), **25** (**D**), **26** (**E**), or **32** (**F**) by 2-bromo-3-hydroxypropanoic acid *N*-benzylamide (**53a**–**e**) was carried out overnight at reflux in dry dimethylformamide (DMF) with *N*,*N*-diisopropylethylamine (DIPEA) and tetra-*n*-butylammonium bromide (TBAB).

#### 2.1.3 Synthesis of Propanoic Acid and Butanoic Acid Derivatives (**64a**,**b**, **65**, **66a**,**b**, **67**, **68**, and **71**–**75**)

**Scheme 4:**
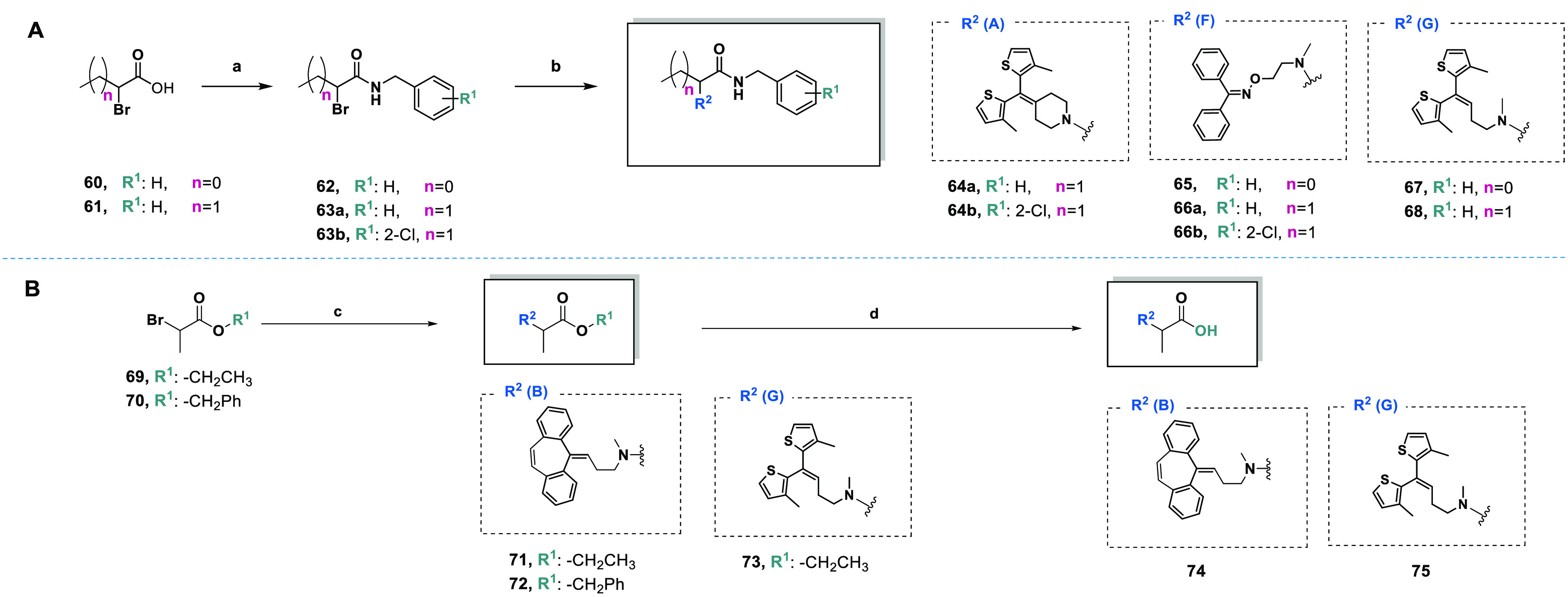
Synthesis of 2-Substituted Derivatives of Propanoic Acid and Butanoic Acid (**64a**,**b**, **65**, **66a**,**b**, **67**, **68**, and **71**–**75**) Reagents and conditions: (a) T3P, TEA, *N*-benzylamine derivative, dry DCM, −17 °C for 30 min and 1 h at rt, argon; (b) suitable amine (**17** (**A**), **32** (**F**), or **33** (**G**)), DIPEA, TBAB, dry DCM, reflux, 12 h; (c) suitable amine (**20** (**B**) or **33** (**G**)), K_2_CO_3_, DCM reflux, 12 h; (d) MeOH, 10 wt % NaOH, 35 °C, 4.5 h.

The 2-substituted derivatives of propanoic acid and butanoic acid (**64a**,**b**, **65**, **66a**,**b**, **67**, **68**, and **71**–**75**) were formed from *N*-benzylamides (**62** or **63a**,**b**, Scheme 4 panel A) or commercially available esters (**69** or **70**, Scheme 4 panel B) following the synthetic route shown in Scheme 4. Alkylation of amines **17** (**A**), **32** (**F**), or **33** (**G**) with 2-bromo-3-propanoic acid (**60**) or 2-bromobutanoic acid (**61**) gave *N*-benzylamides **64a**,**b**, **65**, **66a**,**b**, **67**, and **68**. A similar reaction was carried out for amines **20** (**B**) and **33** (**G**) with 2-bromo-3-propanoic ethyl ester (**69**) or 2-bromo-3-propanoic benzyl ester (**70**) to obtain desired compounds **71**–**73**. Carboxylic acid analogs **74** and **75** were the alkaline hydrolysis products of parent compounds **71** and **73**.

### 2.2 *In Vitro* Evaluation and SAR

The inhibitory potencies of all obtained final compounds were determined for the four mouse GABA transporter subtypes (mGAT1–4). The assay utilized was based on [^3^H]GABA uptake using human embryonic kidney cells (HEK-293) stably expressing mouse GATs according to the literature.^19^ Specific binding affinity toward mGAT1 was determined via a competitive mass spectrometry (MS) binding assay quantified by liquid chromatography–electrospray ionization tandem mass spectrometry (LC–ESI-MS/MS) with NO711 as an unlabeled marker.^50^ The compounds that could reduce GABA uptake or NO711 binding by at least 50% at an inhibitor concentration of 100 μM were considered active. The pIC_50_ or p*K*_i_ values from the [^3^H]GABA uptake or MS binding assays were determined in triplicate samples for competition and in three independent experiments only for compounds with pIC_50_ ≥ 5.00. If at a screening concentration of 100 μM the test compounds could not reduce [^3^H]GABA uptake or NO711 binding below 50% (pIC_50_ = 4.00), the percent of remaining [^3^H]GABA uptake or NO711 binding is given in the presence of 100 μM inhibitor.

**Figure 4:**
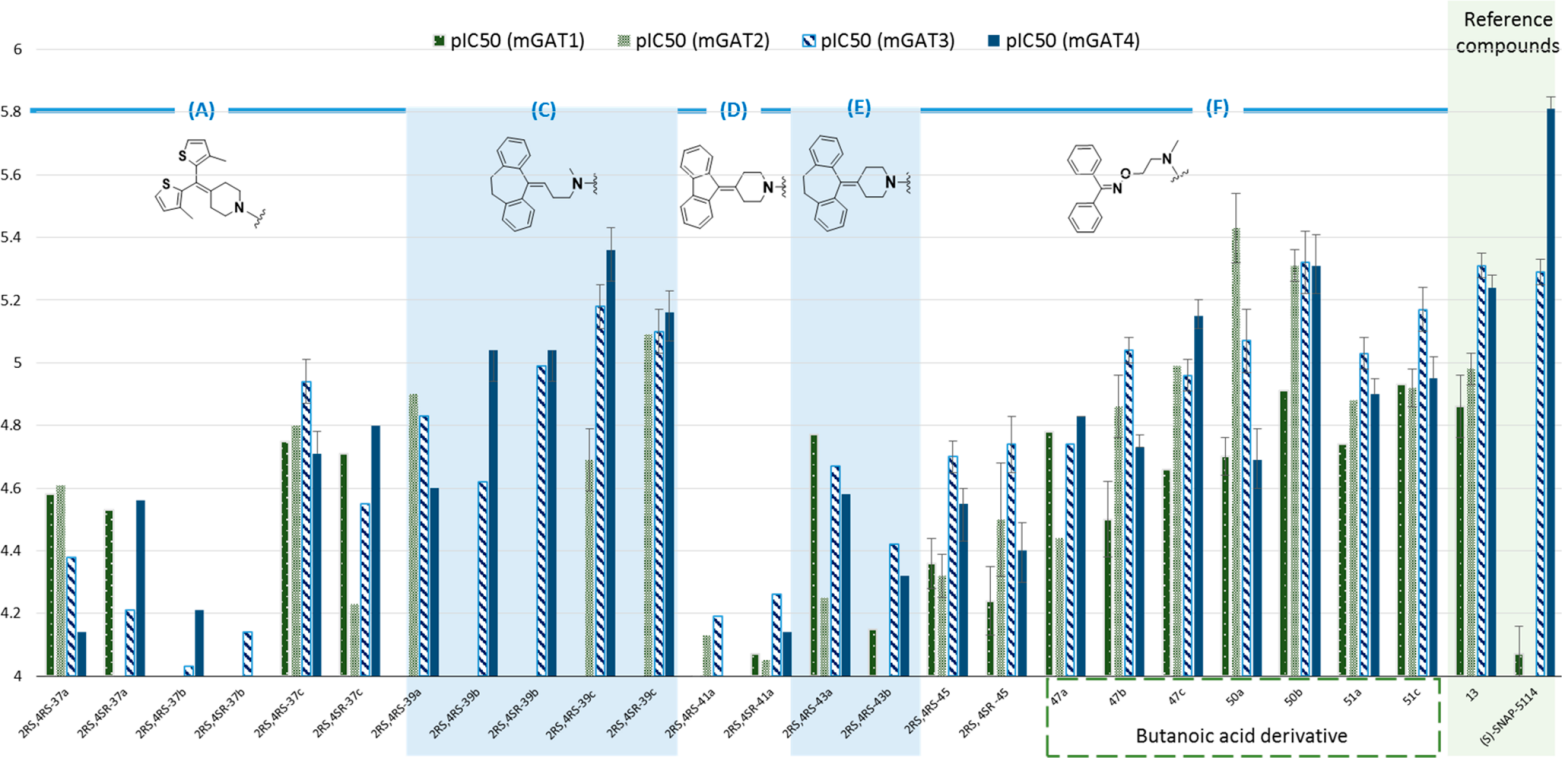
Bar graphs of the pIC_50_ values for the library of 4-hydroxypentanamide (**37a**–**c**, **39a**–**c**, **41a**, **43a**,**b**, **45**), 4-hydroxybutanamide (**47a**–**c**), 4-aminobutanamide (**50a**,**b**), and 4-acetamidobutanamide (**51a**,**b**) derivatives compared to the values for the reference compounds (*S*)-SNAP-5114 (**9**) and **13**. Error bars are not present when only one experiment was performed in triplicate. Additionally, bars are not presented when the pIC_50_ value is lower than 4.00.

In the current approach, we focused on diversifying lipophilic side chain substituents at the α position of *N*-benzylamides. Newly synthesized 4-hydroxypentanoic acid derivatives possessing a suberone-*N*-methylpropan-1-amine (**C**) moiety ((2*RS*,4*RS***/**2*RS*,4*SR*)-**39a**–**c**) and diphenylmethanone *O*-(2-(methylamino)ethyl) oxime (**F**) moiety ((2*RS*,4*RS***/**2*RS*,4*SR*)-**45**) in general showed inhibitory potency comparable with parent compound **13** except for compounds with a more rigid moiety such as 4-(bis(3-methylthiophen-2-yl)methylene)piperidine (**A**) moiety ((2*RS*,4*RS***/**2*RS*,4*SR*)-**37a**–**c**), 4-(suberone)piperidine (**E**) moiety ((2*RS*,4*RS***/**2*RS*,4*SR*)-**43a**,**b**), or 4-(fluorene)piperidine (**D**) moiety ((2*RS*,4*RS***/**2*RS*,4*SR*)-**41**), which displayed reduced activity toward all GABA transporter subtypes (Figure 4, Table 1). On the other hand, when we compared 4-hydroxybutanamide derivatives with diphenylmethanone *O*-(2-(methylamino)ethyl) oxime (**F**) moiety **47a**–**c** with 4-hydroxypentanoic acid analogs 2*RS*,4*RS*-**45** and 2*RS*,4*SR*-**45**, slightly higher inhibitory activity was observed for the 4-hydroxybutanamide analogs (Table 1, Figure 4). Moreover, in the group of oximes containing benzamides ((2*RS*,4*RS***/**2*RS*,4*SR*)-**45**, **47a**–**c**, **50a**,**b**, and **51a**,**b**), the 4-aminobutanamide derivatives (**50a**,**b**) also displayed slightly improved inhibitory activity. Hence, among the oxime derivatives, compound **50a** with a chlorine atom in the 2-position of the benzyl moiety exhibited the highest inhibitory potency toward mGAT2 among all compounds obtained (**50a**, pIC_50_(mGAT2) = 5.43). Acylation of the amino functionality at the 4-position of butanoic acid (**51a**,**b**) did not change the inhibitory potency profile against mGAT1–4 (Table 1, Figure 4). An interesting effect was observed for derivatives **39a**–**c** with a suberone-*N*-methylpropan-1-amine (**C**) moiety. In this group of compounds, we identified two dual mGAT3/4 subtype selective inhibitors (2*RS*,4*RS*-**39b** and 2*RS*,4*SR*-**39b**) with a similar GAT preference to that of (*S*)-SNAP-5114 (**9**) but with reduced potency. On the other hand, compound 2*RS*,4*RS*-**39c**, with a methyl group in the *para* position of the benzyl moiety, was found to be the most potent mGAT4 inhibitor (pIC_50_(mGAT4) = 5.36) with favorable GAT subtype selectivity.

**Table 1:**
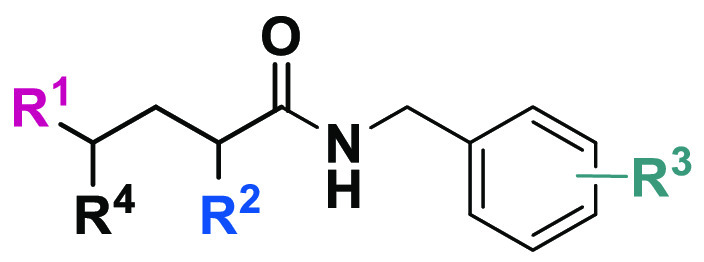
Inhibitory Potencies (pIC_50_ ± Standard Error of the Mean (SEM)) toward mGAT1–4 Determined by the [^3^H]GABA Uptake Experiments and mGAT1 Binding Affinities (p*K*_i_ ± SEM) from the MS Binding Assays of the Obtained Compounds

					pIC_50_ a ± SEM				p*K*_i_ a ± SEM
compd	R^1^	R^2^	R^3^	R^4^	mGAT1	mGAT2	mGAT3	mGAT4	mGAT1
2*RS*,4*RS*-**37a**	Me	A	H	OH	4.58	4.61	4.38	4.14	100 μM: 74%
2*RS*,4*SR-***37a**	Me	A	H	OH	4.53	100 μM: 60%	4.21	4.56	100 μM: 78%
2*RS*,4*RS*-**37b**	Me	A	2-Cl	OH	100 μM: 52%	100 μM: 58%	4.03	4.21	100 μM: 94%
2*RS*,4*SR-***37b**	Me	A	2-Cl	OH	100 μM: 50%	100 μM: 69%	4.14	100 μM: 56%	100 μM: 108%
2*RS*,4*RS*-**37c**	Me	A	4-F	OH	4.75	4.80	4.94 ± 0.07	4.71	100 μM: 76%
2*RS*,4*SR-***37c**	Me	A	4-F	OH	4.71	4.23	4.55	4.8	100 μM: 73%
2*RS*,4*RS*-**39a**	Me	C	4-F	OH	100 μM: 51%	4.90	4.83	4.60	100 μM: 110%
2*RS*,4*RS*-**39b**	Me	C	4-Cl	OH	100 μM: 56%	100 μM: 73%	4.62	5.04 ± 0.10	100 μM: 84%
2*RS*,4*SR-***39b**	Me	C	4-Cl	OH	100 μM: 51%	100 μM: 73%	4.99	5.04 ± 0.10	100 μM: 87%
2*RS*,4*RS*-**39c**	Me	C	4-Me	OH	100 μM: 52%	4.69	5.18 ± 0.07	5.36 ± 0.10	100 μM: 69%
2*RS*,4*SR-***39c**	Me	C	4-Me	OH	100 μM: 51%	5.09	5.10 ± 0.07	5.16 ± 0.09	100 μM: 73%
2*RS*,4*RS*-**41a**	Me	D	H	OH	100 μM: 49%	4.13	4.19	52%	100 μM: 92%
2*RS*,4*SR-***41a**	Me	D	H	OH	4.07	4.05	4.26	4.14	100 μM: 89%
2*RS*,4*RS*-**43a**	Me	E	H	OH	4.77	4.25	4.67	4.58	100 μM: 77%
2*RS*,4*SR-***43a**	Me	E	H	OH	4.71	4.23	4.55	4.80	100 μM: 73%
2*RS*,4*RS*-**43b**	Me	E	4-F	OH	4.15	100 μM: 59%	4.42	4.32	100 μM: 86%
2*RS*,4*SR-***43b**	Me	E	4-F	OH	100 μM: 54%	100 μM: 80%	100 μM: 87%	100 μM: 62%	100 μM: 102%
2*RS*,4*RS*-**45**	Me	F	H	OH	4.36 ± 0.08	4.34 ± 0.07	4.70 ± 0.05	4.55 ± 0.12	100 μM: 100%
2*RS*,4*SR-***45**	Me	F	H	OH	4.24 ± 0.11	4.50 ± 0.18	4.74 ± 0.09	4.40 ± 0.06	100 μM: 86%
**47a**	H	F	H	OH	4.78	4.44	4.74	4.83	100 μM: 87%
**47b**	H	F	2-Cl	OH	4.50 ± 0.12	4.86 ± 0.10	5.04 ± 0.04	4.73 ± 0.12	100 μM: 86%
**47c**	H	F	2,4-di-Cl	OH	4.66	4.99	4.96 ± 0.05	5.15 ± 0.04	100 μM: 82%
**50a**	H	F	2-Cl	NH_2_	4.70 ± 0.06	5.43 ± 0.11	5.07 ± 0.10	4.69 ± 0.09	4.48 ± 0.13
**50b**	H	F	3,4-di-Cl	NH_2_	4.91	5.31 ± 0.05	5.32 ± 0.10	5.31 ± 0.09	4.48
**51a**	H	F	2-Cl	NH(CO)CH_3_	4.74	4.88	5.03 ± 0.05	4.90	100 μM: 74%
**51b**	H	F	3,4-di-Cl	NH(CO)CH_3_	4.93	4.92 ± 0.06	5.17 ± 0.07	4.95	100 μM: 72%
tiagabine (**5**)^19^					6.88 ± 0.12	100 μM: 52%	100 μM: 64%	100 μM: 73%	7.43 ± 0.11 ^51^
(*S*)-SNAP-5114 (**3**)^19^					4.07 ± 0.09	100 μM: 56%	5.29 ± 0.04	5.81 ± 0.10	4.56 ± 0.02
DDPM-859 (**4**)^22^					4.19 ± 0.07	4.12 ± 0.08	4.85 ± 0.04	5.78 ± 0.03	nd
DDPM-2571 (**5**)^20^					8.27 ± 0.03	4.31	4.35	4.07	8.29 ± 0.02
**13**(25)					4.86 ± 0.10	4.98 ± 0.05	5.31 ± 0.04	5.24 ± 0.05	100 μM: 77%

a Data are given as the mean ± SEM of three independent experiments that were performed in triplicate. The results presented as a percent represent [^3^H]GABA uptake or NO711 binding in the presence of 100 μM inhibitor. Data without the SEM imply that only one experiment was performed in triplicate. nd: not determined.

**Table 2:**
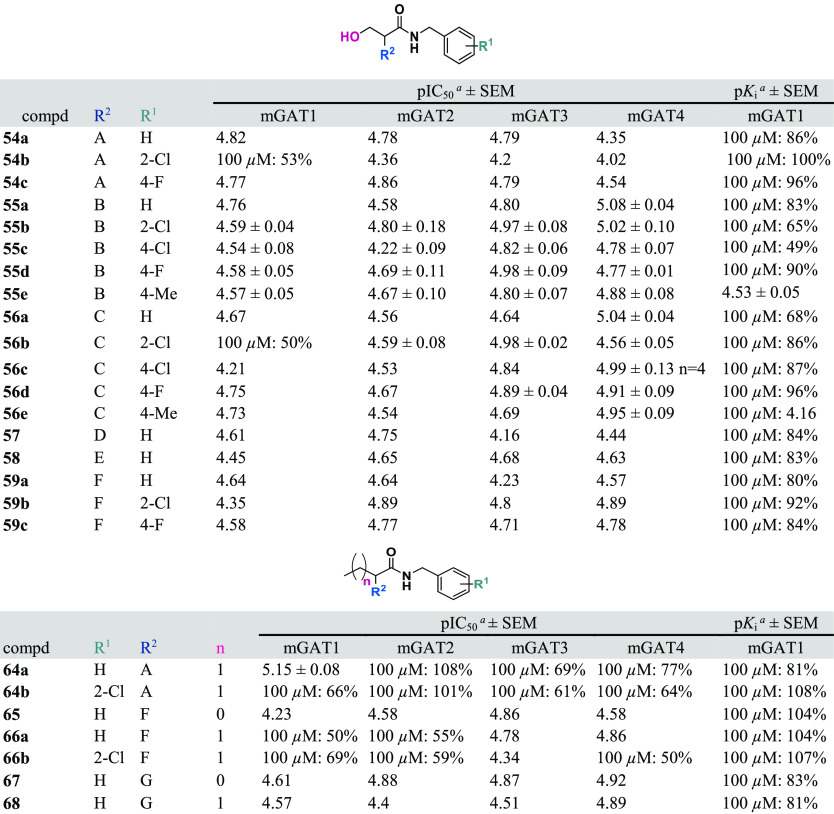
Inhibitory Potencies (pIC_50_ ± SEM) toward mGAT1–4 Determined from the [^3^H]GABA Uptake Experiments and mGAT1 Binding Affinities (p*K*_i_ ± SEM) from the MS Binding Assays of the Obtained Compounds

a Data are given as the mean ± SEM of three independent experiments that were performed in triplicate. The results presented as a percent represent [^3^H]GABA uptake or NO711 binding in the presence of 100 μM inhibitor. Data without the SEM imply that only one experiment was performed in triplicate. n.d.: not determined.

**Figure 5:**
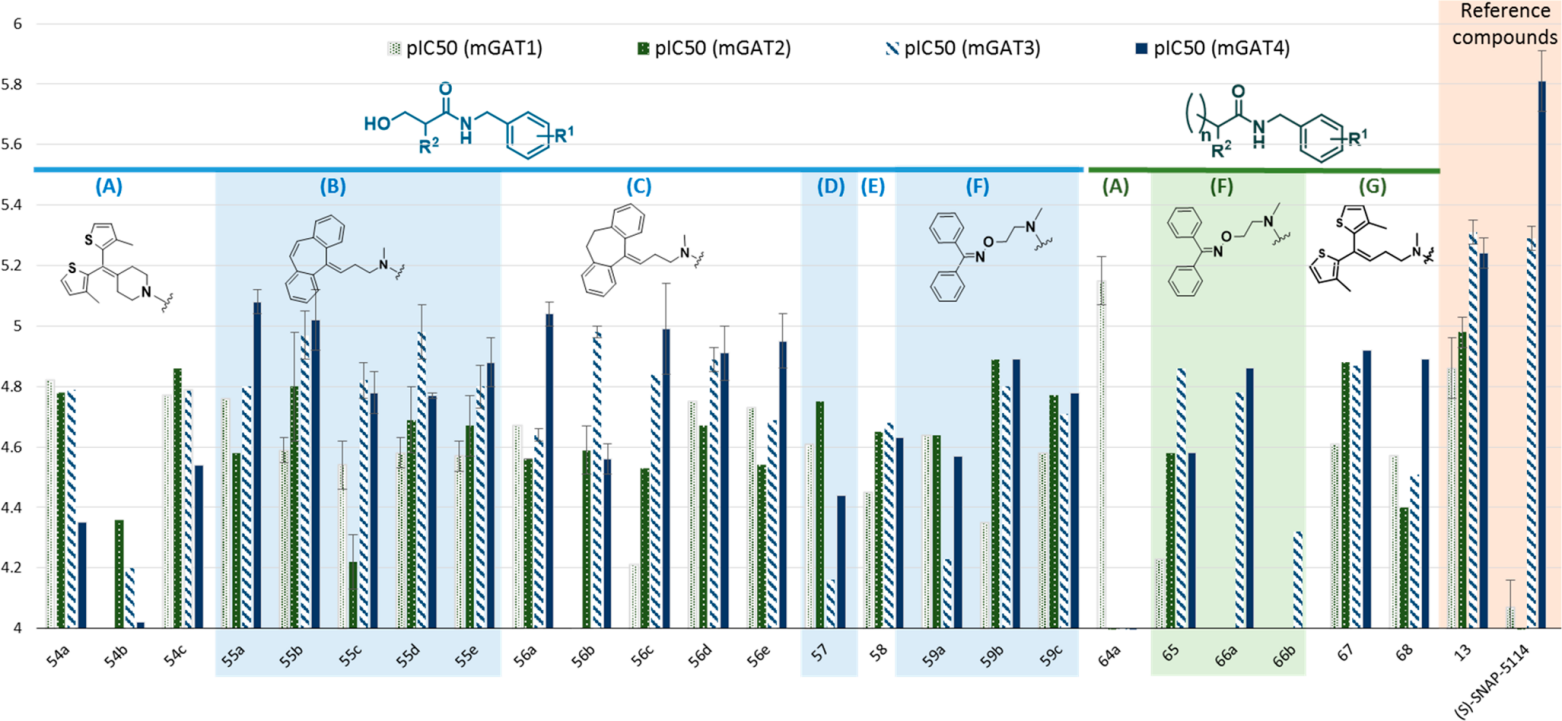
Bar graphs of the pIC_50_ values for the library of 3-hydroxypropanamide (**54a**–**c**, **55a**–**e**, **56a**–**e**, **57**, **58**, and **59a**–**c**), propanoic acid, and butanoic acid derivatives (**64a**,**b**, **65**, **66a**,**b**, **67**, and **68**) compared to the values for the reference compounds (*S*)-SNAP-5114 (**9**) and **13**. Error bars are not present when only one experiment was performed in triplicate. Additionally, bars are not presented when the pIC_50_ value is lower than 4.00.

The exchange of a hydroxyl group for a methyl group is represented in many of the 3-hydroxypropanamide derivatives (**54a**–**c**, **55a**–**e**, **56a**–**e**, **57**, **58**, and **59a**–**c**) and propanoic acid and butanoic acid derivatives (**57**, **64a**,**b**, **65**, **66a**,**b**, **67**, **68**, and **71**–**75**; Table 2, Figure 5). The most potent synthesized serine derivatives have a tricyclic building block as the lipophilic substituent (dibenzocycloheptadiene moiety; **C**) in the α position of the *N*-benzylamides, **56a**–**e** (Table 2, Figure 5). **56a**–**e** showed the highest inhibitory activity toward mGAT3/4 (pIC_50_ in a range of 5.46–5.04). Notably, one mGAT1-selective butanoic acid derivative (**64a**, pIC_50_(mGAT1) = 5.15) has a rigid bisthiophene moiety (**A**) in the α position. Surprisingly, the small structural change in **64a**, with a hydrogen substituted for the chlorine atom in the *N*-benzylamide moiety (**64b**), results in a loss of activity. Unfortunately, we failed to observe an increase in inhibitory activity when the *N*-benzylamide moiety was exchanged for a carboxylic acid group (**74** and **75**), ethyl ester (**71** and **73**), or benzyl ester (**72**) (Table 3).

**Table 3:**
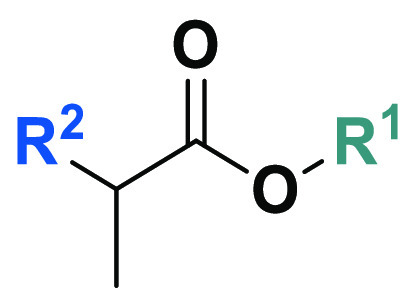
Inhibitory Potencies (pIC_50_ ± SEM) toward mGAT1–4 Determined from the [^3^H]GABA Uptake Experiments and mGAT1 Binding Affinities (p*K*_i_ ± SEM) from the MS Binding Assays of the Obtained Compounds

			pIC_50_ a ± SEM				p*K*_i_ a ± SEM
compd	R^1^	R^2^	mGAT1	mGAT2	mGAT3	mGAT4	mGAT1
**71**	–CH_2_CH_3_	B	100 μM: 52%	100 μM: 54%	4.66	4.20	100 μM: 103%
**72**	–CH_2_Ph	B	100 μM: 98%	100 μM: 63%	100 μM: 86%	100 μM: 97%	100 μM: 109%
**73**	–CH_2_CH_3_	G	100 μM: 55%	100 μM: 52%	4.6	4.28	100 μM: 94%
**74**	H	G	100 μM: 55%	100 μM: 55%	100 μM: 62%	100 μM: 77%	4.54
**75**	H	B	100 μM: 71%	100 μM: 59%	100 μM: 52%	100 μM: 60%	100 μM: 81%
tiagabine (**5**)^19^			6.88 ± 0.12	100 μM: 52%	100 μM: 64%	100 μM: 73%	7.43 ± 0.11 ^51^
(*S*)-SNAP-5114 (**3**)^19^			4.07 ± 0.09	100 μM: 56%	5.29 ± 0.04	5.81 ± 0.10	nd
DDPM-859 (**4**)^22^			4.19 ± 0.07	4.12 ± 0.08	4.85 ± 0.04	5.78 ± 0.03	nd
DDPM-2571 (**5**)^20^			8.27 ± 0.03	4.31	4.35	4.07	8.29 ± 0.02
**13**(25)			4.86 ± 0.10	4.98 ± 0.05	5.31 ± 0.04	5.24 ± 0.05	100 μM: 77%

a Data are given as the mean ± SEM of three independent experiments that were performed in triplicate. The results presented as a percent represent [^3^H]GABA uptake or NO711 binding in the presence of 100 μM inhibitor. Data without the SEM imply that only one experiment was performed in triplicate. nd: not determined.

### 2.3 Molecular Modeling

To determine the binding mode of the tested compounds with GATs, molecular modeling calculations were performed. For this purpose, models of human GAT-1, BGT-1, GAT-2, and GAT-3 were used, as they are the targets for the new inhibitors. Although the activity of the compounds was tested on mouse transporters, we assumed that the compounds could bind to human and mouse in the same place and with comparable affinity due to only slight differences in the amino acid sequences, mainly concerning the N- and C-terminus.^32,52^ Homology modeling of the GABA transporters and the differences between the structures of the particular types of these proteins were described in more detail in our previous work.^53^

**Figure 6:**
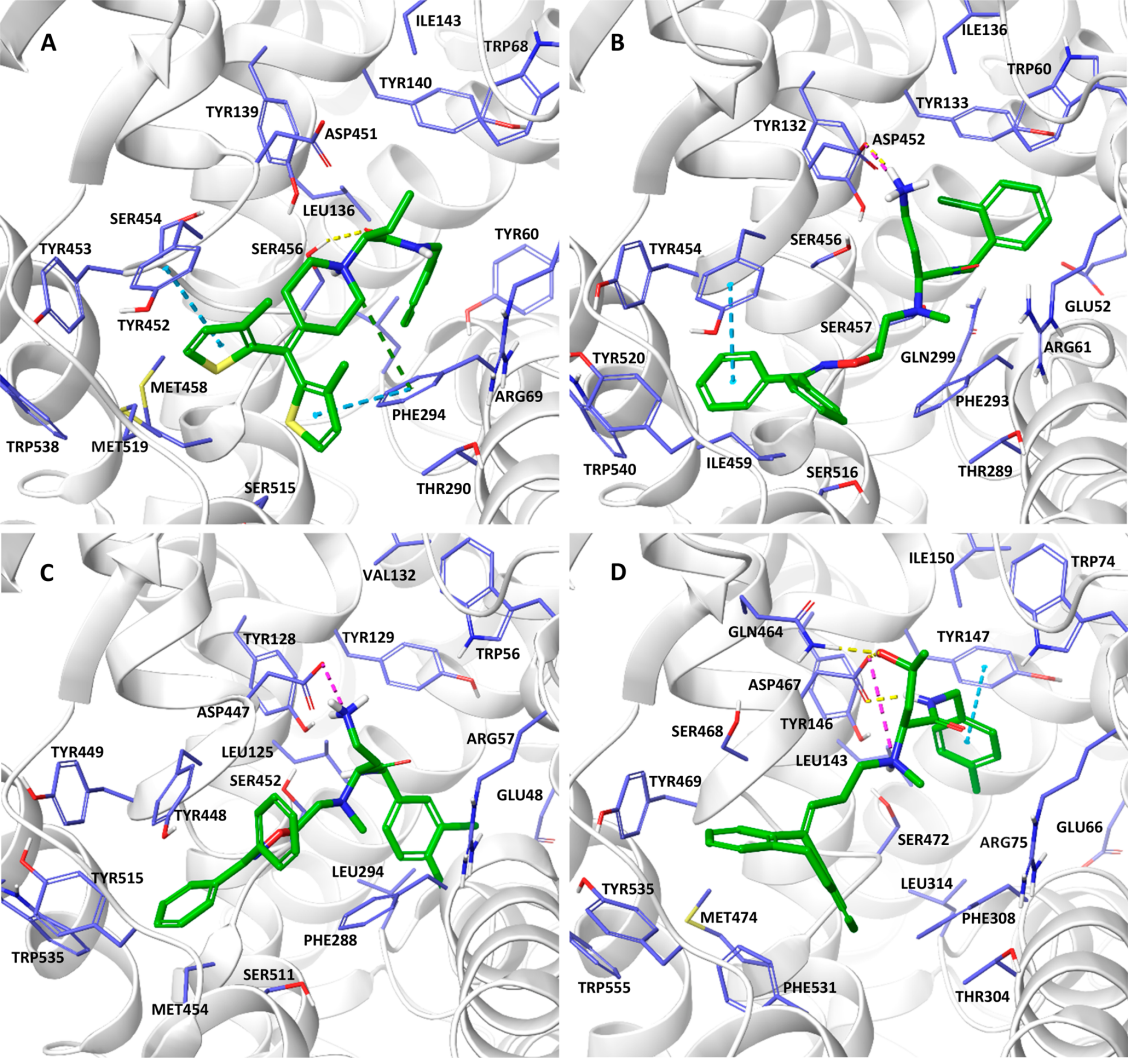
Binding modes of compound (*S*)-**64a** in GAT-1 (A); compound (*S*)-**50a** in BGT-1 (B); compound (*S*)-**50b** in GAT-2 (C); compound (2*S,*4*S*)-**39c** in GAT-3 (D). CH−π and π–π interactions are marked with blue dashes, ionic interactions with pink dashes, cation−π interactions with green dashes, and hydrogen bonds with yellow dashes.

**Figure 7:**
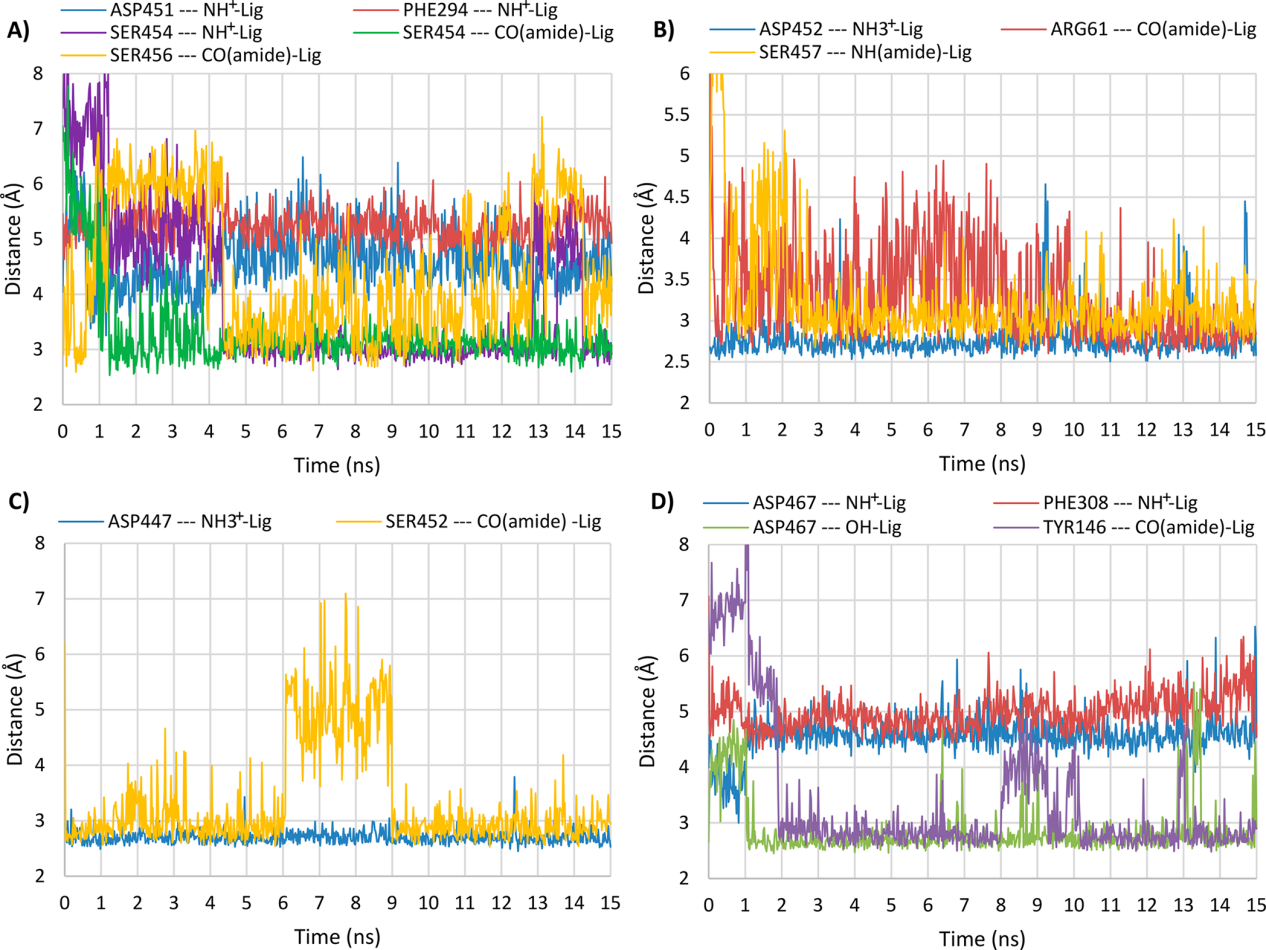
Distance changes between the particular transporter residues and ligand moieties during molecular dynamics simulations for compound (*S*)-**64a** in GAT-1 (A), compound (*S*)-**50a** in BGT-1 (B), compound (*S*)-**50b** in GAT-2 (C), and compound (2*S,*4*S*)-**39c** in GAT-3 (D).

Molecular docking studies indicated that compounds generally bind in a similar manner in all types of transporters (Figure 6). The compounds are located along the vestibule of the transporters, which is consistent with our previous results obtained for similar 4-amino- and 4-hydroxybutanamide derivatives.^53^ Molecular dynamics simulations were performed on representatives of the most active compounds toward particular types of transporters to confirm the stability of the presented binding modes and created interactions (Figure 7, Figure S1). For compounds with an undefined absolute configuration, all possible stereoisomers were investigated. For the tested compounds, more consistent arrangements and beneficial interactions among all types of transporters were generally observed for the α carbon *S* isomers.

In the case of GAT-1, the bisthiophene fragment in the most active compound **64a** creates hydrophobic interactions and CH−π stacking with TYR452 and PHE294 near the entrance to the transporter (Figure 6, panel A). The amino group of **64a** is located between the aromatic ring of PHE294 and the carboxyl moiety of ASP451, i.e., residues that are part of the extracellular gate. This arrangement allows for the formation of a stable ionic bond with ASP451 and cation−π interaction with PHE294. During molecular dynamics simulation, as a result of the bending of the nonhelical fragment of domain 10, the side chain of SER454 approached the protonated amino group of the compound. This enabled the creation of a stable hydrogen bond between these groups (Figure 7, panel A). The ethyl substituent in the α position reaches TRP68, creating hydrophobic interactions. The amide carbonyl group forms a hydrogen bond with the side chain of SER456. During the dynamic calculations, a hydrogen bond with the hydroxyl group of SER454 was also observed. The benzyl fragment reaches into the S1 site, creating hydrophobic interactions mainly with LEU300, LEU460, LEU136, and PHE294. This arrangement appears to be beneficial considering that the S1 site in GAT-1 is the most hydrophobic among all types of transporters.

The diphenylmethylidene fragment of compound **50a**, the most active toward BGT-1, locates itself in this transporter close to the EL6 loop compared to the poses observed in GAT-1. Compound **50a** participates in hydrophobic interactions mainly with TYR520, TRP540, TYR454, ILE459, and TYR453 as well as π–π stacking with TYR453 (Figure 6, panel B). The protonated primary amine is engaged in a salt bridge with ASP452. The amide group is located close to SER457, which enables the creation of a hydrogen bond during molecular dynamics simulation (Figure 7, panel B). During the simulation, a change in the conformation of the ARG61 side chain was also observed. This provided an additional hydrogen bond with the aforementioned amide group. The 2-chlorobenzyl fragment is located, contrary to that observed in the GAT-1 transporter, above the extracellular gate in the S2 site, creating hydrophobic interactions mainly with TYR133, TYR132, and TRP60. This arrangement is beneficial because the S1 site in BGT-1 is more polar than in other types of transporters. Additionally, a halogen bond between the chlorine atom of **50a** and the carboxyl group of ASP452 was observed. In the case of another relatively highly active compound **50b**, the diphenylmethylidene and 4-aminobutanamide fragment retain the interactions described above, whereas the 3,4-dichlorobenzyl fragment is located at the level of the extracellular gate, creating hydrophobic and CH−π interactions with TYR133.

In GAT-2, the most active compound **50b** is placed similarly as in the BGT-1 transporter. The diphenylmethylidene fragment forms hydrophobic interactions mainly with TYR448, TYR515, and MET454. The protonated amino group of this compound creates a stable salt bridge with ASP447 (Figure 6, panel C). The amide moiety is located near the nonhelical fragment of TM10. During the molecular dynamics simulation, the fragment containing this moiety rotates which enables creation of a hydrogen bond between the carbonyl oxygen of the amide group and the hydroxyl group of SER452. In contrast to the position in the BGT-1 transporter, the 3,4-dichlorobenzyl fragment reaches the inside of the S1 site in GAT-2. It forms hydrophobic interactions mainly with LEU294, LEU456, and PHE288.

In the case of GAT-3, the diaromatic fragments of the most active compounds **50b** and 2*RS*,4*RS*-**39c** are in a similar position compared to that observed in the GAT-2 transporter. However, due to the presence of SER468 and PHE531, which are replaced by tyrosine and serine residues, respectively, in the other transporters, the diphenylmethylidene and dibenzocycloheptadiene fragments are bound slightly higher within the vestibule. These moieties engage in hydrophobic interactions mainly with the above-mentioned PHE531, as well as with TYR535 and TYR469. The protonated amino group forms an ionic bond (compound 2*RS*,4*RS*-**39c**) or a salt bridge (compound **50b**) with ASP467, similar to the previously described compounds (Figure 6, panel D). During the molecular dynamics simulation performed for compound 2*RS*,4*RS*-**39c**, this protonated amino group slightly moves away from ASP467 while simultaneously approaching the aromatic ring of PHE308, which enables cation−π interaction while maintaining an ionic bond with ASP467 (Figure 7, panel D). At the same time the hydroxyl moiety creates a stable hydrogen bond with ASP467. The amide groups of both described compounds are also located close to ASP467 being involved in the hydrogen bond with this residue. However, over the course of the dynamics simulation for 2*RS*,4*RS*-**39c** it was observed that the amide moiety can move closer to the side chain of TYR146, creating a hydrogen bond. The 4-methylbenzyl and 3,4-dichlorobenzyl fragments are located at the level of the lower part of the extracellular gate, creating hydrophobic and CH−π interactions with TYR147. Compound **50b** additionally forms a halogen bond with the amide moiety of GLY71.

### 2.4 Hepatotoxicity and Cytotoxicity

In this study we investigated three representative compounds, 2*RS*,4*RS*-**39c**, **50a**, and **56a**, for *in vitro* studies to verify their safety in HepG2 and HEK-293 cells. Among all obtained compounds, compound 2*RS*,4*RS*-**39c**, a 4-hydroxypentanamide derivative, and compound **50a**, a 4-aminobutanamide derivative, were selected for further studies because they provided the highest inhibitory activity toward mGAT4 (pIC_50_ = 5.36 ± 0.10) or mGAT2 (pIC_50_ = 5.43 ± 0.11), respectively. Compound **56a**, a 3-hydroxypropanamide derivative, was selected as one of two compounds with moderate subtype selectivity for mGAT4.

**Figure 8:**
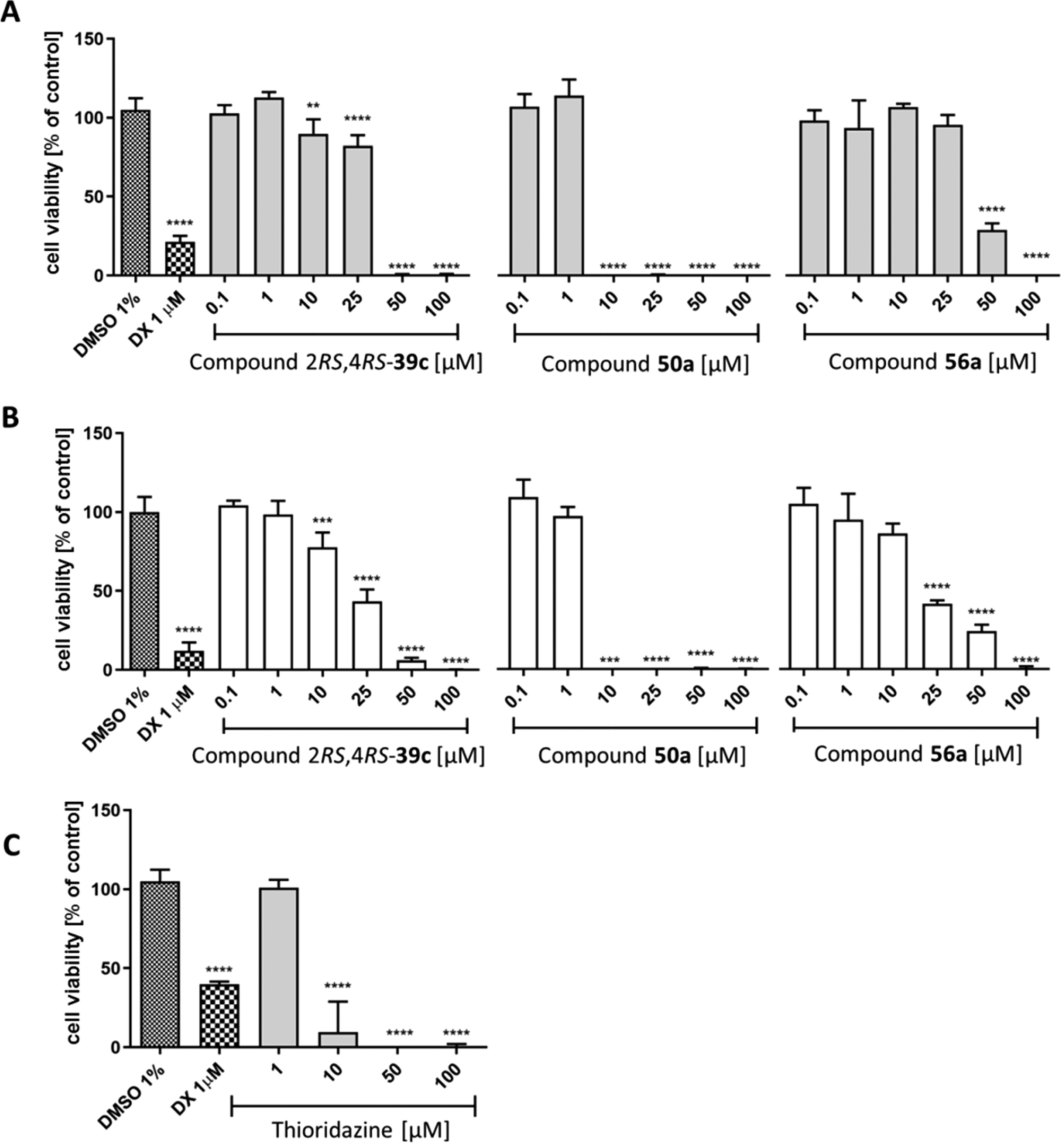
Influence of 2*RS*,4*RS*-**39c**, **50a**, **56a**, and the reference cytostatic drug DX on the viability of hepatoma HepG2 cells (A) and HEK-293 cells (B) after 72 h of incubation. The influence of thioridazine on the viability of hepatoma HepG2 cells (C) after 72 h of incubation. Statistical significance (****p < 0.0001) was analyzed by GraphPad Prism 8.0.1 software using one-way ANOVA and Bonferroni’s multiple comparison post hoc test.

To investigate the safety of compounds 2*RS*,4*RS*-**39c**, **50a**, and **56a**, a HepG2 hepatoma cell-based hepatotoxicity assay was used. Compounds were tested at six concentrations (0.1–100 μM). The results showed that at lower compound concentrations (0.1 and 1 μM), none of the tested compounds caused a statistically significant decrease in HepG2 cell viability and thus were not hepatotoxic in comparison to doxorubicin (DX) at 1 μM (Figure 8, panel A). However, only compound **56a** did not induce significant hepatotoxicity after 72 h of incubation at concentrations up to 25 μM. A statistically significant (*p* < 0.0001) decrease in HepG2 cell viability was observed for **56a** only at the highest concentrations of 50 and 100 μM (Figure 8, panel A). Compound 2*RS*,4*RS*-**39c** was slightly more toxic than **56a**, as it showed a statistically significant (*p* < 0.0001) decrease in HepG2 cell viability at 25 μM, whereas **56a** showed 100% viability compared to the control at this concentration (1% DMSO in culture media). Compound **50a** significantly eradicated cell viability at concentrations between 10 and 100 μM (*p* < 0.0001). Nevertheless, these results are in accordance with the hepatotoxicity examination of thioridazine, an antipsychotic agent that is still in use (Figure 8, panel C).^54^

Subsequently, a similar study was performed with the HEK-293 cell line. Overall, compounds 2*RS*,4*RS*-**39c** and **56a** showed stronger toxic effects than in the HepG2 assay, where a significant (****p < 0.0001) decrease in HEK-293 cell viability was observed at the concentration of 25 μM. On the other hand, compound **50a** showed a comparable safety profile in HEK-293 and HepG2 cells.

Considering the observed antiproliferative effects of compounds 2*RS*,4*RS*-**39c** and **56a** at 25 μM and at 1 μM for compound **50a**, it can be generalized that these effects were still lower than those for DX (at 1 μM) or comparable with those of thioridazine (over the concentration range of 10–100 μM). In this respect and based on the very promising biological results, compounds 2*RS*,4*RS*-**39c**, **50a**, and **56a** were selected for further investigation to elevate their antinociceptive activity in mouse models of NP. However, taking into account the obtained range of toxicity of the tested compounds, we assumed that further drug-like property optimization is required to obtain an acceptable safety profile, which will be the next stage of our research.

### 2.5 *In Vivo* Pharmacological Evaluation (Mouse Models of Neuropathic Pain)

In this part of the present research, we assessed if the compounds 2*RS*,4*RS*-**39c, 50a**, and **56a** display analgesic (antiallodynic and antihyperalgesic) properties in NP conditions.

For this purpose, we used three mouse models of NP, namely, chemotherapy-induced NP models (i.e., the oxaliplatin model and the paclitaxel model) and the diabetic NP model induced by streptozotocin (STZ). We assessed the effect of the test compounds on tactile allodynia and thermal (heat or cold) hyperalgesia in the von Frey, hot plate, or cold plate tests, respectively. Since oxaliplatin is responsible for inducing cold hypersensitivity, in both humans and experimental animals,^55,56^ the cold plate test was used to assess the effect of the test compounds on the thermal pain threshold in oxaliplatin-treated mice. In the two other NP models the hot plate test was applied to measure heat pain threshold in paclitaxel- and STZ-treated mice.^57,58^ Since impaired motor coordination is also observed in NP conditions, we additionally tested the influence of compounds 2*RS*,4*RS*-**39c**, **50a**, and **56a** on motor coordination in the rotarod test.

#### 2.5.1 Oxaliplatin-Induced Peripheral Neuropathy: Influence on Tactile Allodynia (von Frey Test)

**Figure 9:**
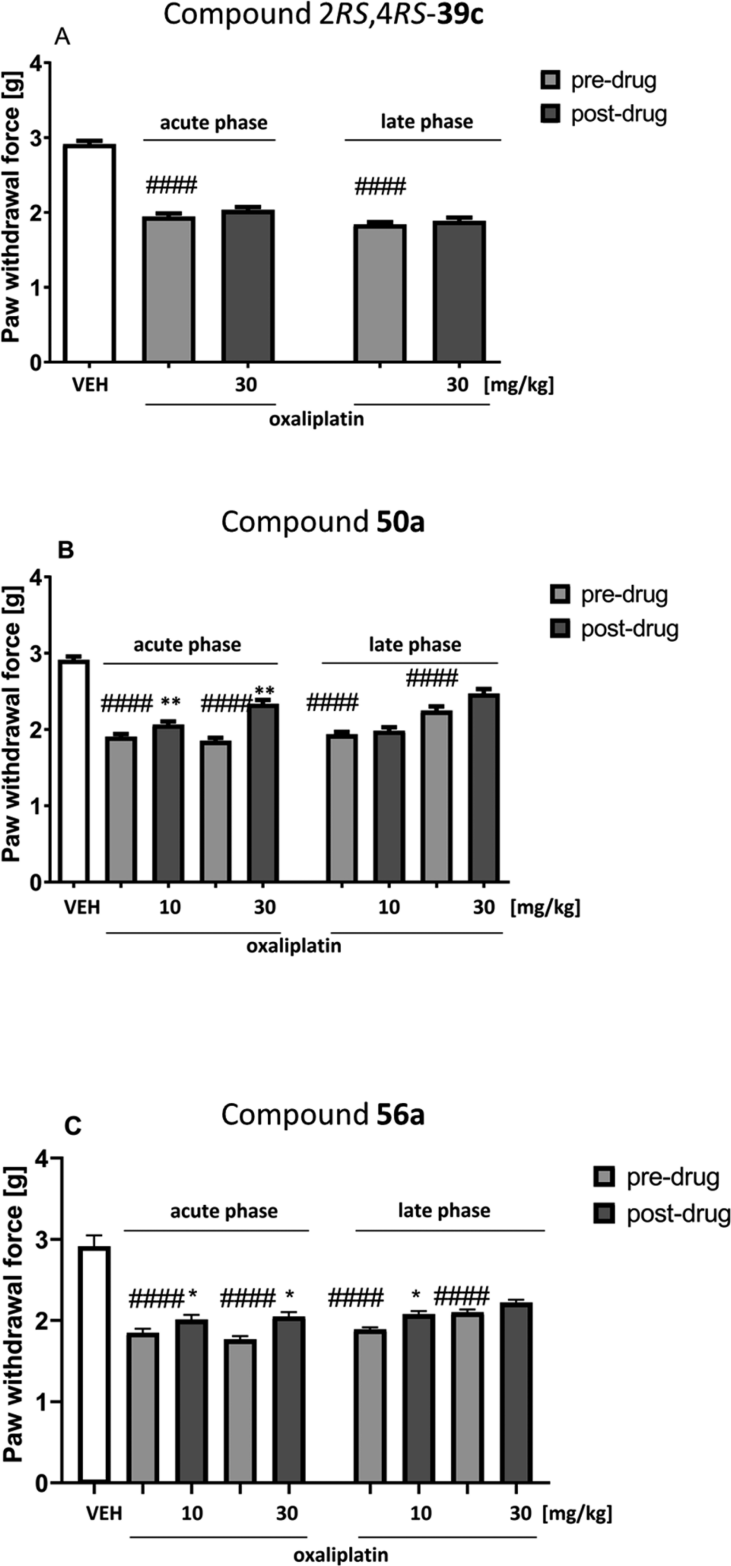
Effects of intraperitoneally administered 2*RS*,4*RS*-**39c** (A), **50a** (B), and **56a** (C) on the mechanical nociceptive threshold in a mouse oxaliplatin-induced NP model measured using the von Frey test in the early phase (on the day of oxaliplatin injection) and in the late phase (7 days after oxaliplatin injection) of neuropathy. The results are shown as the mean (±SEM) force applied to elicit paw withdrawal. Statistical analysis: one-way analysis of variance followed by Tukey’s post hoc comparison. Significance vs paw withdrawal threshold of control, nonneuropathic mice: ^####^*p* < 0.0001. Significance vs predrug (after oxaliplatin) paw withdrawal threshold: *p < 0.05, **p < 0.01. In vehicle-treated mice (VEH), pain sensitivity threshold measurements were taken in the same manner and at the same time points as in the oxaliplatin-treated groups, but vehicle-treated mice were not treated with oxaliplatin; *n* = 8–10.

In the early phase of oxaliplatin-induced neuropathy, an overall effect of treatment on the mechanical nociceptive threshold was observed (2*RS*,4*RS*-**39c**, *F*[1.904, 23.80] = 180.6, *p* < 0.0001; **50a**, *F*[3.037, 24.30] = 136.7, *p* < 0.0001; **56a**, *F*[2.371, 21.34] = 83.50, *p* < 0.0001). In this early phase of neuropathy, the administration of oxaliplatin significantly lowered the pain threshold for mechanical stimulation (*p* < 0.0001 vs vehicle-treated nonneuropathic mice) (Figure 9). Compound 2*RS,*4*RS*-**39c** was not effective in this phase of oxaliplatin-induced neuropathy (Figure 9, panel A). Compound **50a** at both doses significantly elevated the pain threshold for mechanical stimulation (*p* < 0.01 vs predrug paw withdrawal threshold) (Figure 9, panel B). Additionally, both doses of compound **56a** reduced tactile allodynia in the acute phase of oxaliplatin-induced neuropathy (*p* < 0.05 vs predrug paw withdrawal threshold) (Figure 9, panel C).

In the late phase of oxaliplatin-induced neuropathy, an overall effect of treatment on the mechanical nociceptive threshold was observed (2*RS*,4*RS*-**39c**, *F*[1.705, 13.64] = 282.0, *p* < 0.0001; **50a**, *F*[2.354,18.83] = 83.09, *p* < 0.0001; **56a**, *F*[2.651, 23.86] = 108.3, *p* < 0.0001). In this phase, only compound **56a** at a dose of 10 mg/kg showed antiallodynic properties (significant at *p* < 0.05 vs predrug paw withdrawal threshold) (Figure 9, panel C).

#### 2.5.2 Oxaliplatin-Induced Peripheral Neuropathy: Influence on Cold Hyperalgesia (Cold Plate Test)

**Figure 10:**
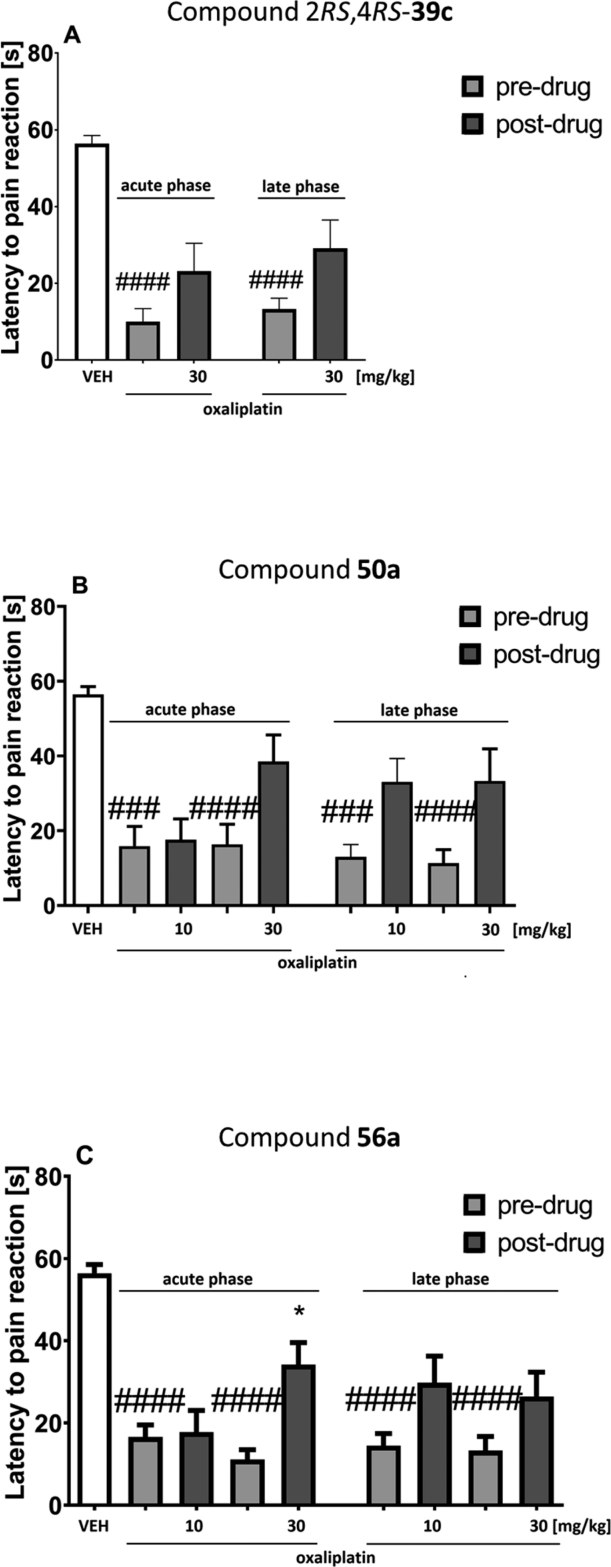
Effects of intraperitoneally administered 2*RS*,4*RS*-**39c** (A), **50a** (B), and **56a** (C) on the thermal (cold) pain threshold in a mouse oxaliplatin-induced NP model measured using the cold plate test in the early phase (on the day of oxaliplatin injection) and in the late phase (7 days after oxaliplatin injection) of neuropathy. The results are shown as the mean (±SEM) latency to pain reaction. Statistical analysis: one-way analysis of variance followed by Tukey’s post hoc comparison. Significance vs latency of control, nonneuropathic mice: ^###^*p* < 0.001, ^####^*p* < 0.0001. Significance vs predrug (after oxaliplatin) latency to pain reaction: *p < 0.05. In the vehicle-treated mice (VEH), measurements of the pain sensitivity threshold were taken in the same manner and at the same time points as in the oxaliplatin-treated groups, but vehicle-treated mice were not treated with oxaliplatin; *n* = 8–10.

In the early phase of oxaliplatin-induced neuropathy, an overall effect of treatment on the thermal (cold) nociceptive threshold was observed (2*RS*,4*RS*-**39c**, *F*[1.393, 16.02] = 30.05, *p* < 0.0001; **50a**, *F*[2.634, 21.07] = 16.57, *p* < 0.0001; **56a**, *F*[2.701, 24.31] = 27.96, *p* < 0.0001). In this phase, the administration of oxaliplatin significantly lowered the pain threshold for cold stimulation (*p* < 0.001 vs vehicle-treated nonneuropathic mice) (Figure 10). Compounds 2*RS*,4*RS*-**39c** and **50a** were not effective in this phase of oxaliplatin-induced neuropathy (Figure 10, panels A and B). Compound **56a** at a dose of 30 mg/kg reduced cold hyperalgesia in the acute phase of oxaliplatin-induced neuropathy (*p* < 0.05 vs predrug paw withdrawal threshold) (Figure 10, panel C).

In the late phase of oxaliplatin-induced neuropathy, an overall effect of treatment was observed (2*RS*,4*RS*-**39c**, *F*[1.171, 14.64] = 23.22, *p* < 0.001; **50a**, *F*[3.125, 24.38] = 9.974, *p* < 0.001; **56a**, *F*[2.508, 22.57] = 16.37, *p* < 0.0001). In this phase, none of the tested compounds showed antihyperalgesic properties (Figure 10).

On the basis of the results obtained in the oxaliplatin-induced neuropathic pain model, i.e., due to lack of activity of 2*RS*,4*RS*-**39c**, for further pain tests and NP models, only the compounds **50a** and **56a** were selected.

#### 2.5.3 Paclitaxel-Induced Peripheral Neuropathy: Influence on Tactile Allodynia (von Frey Test)

**Figure 11:**
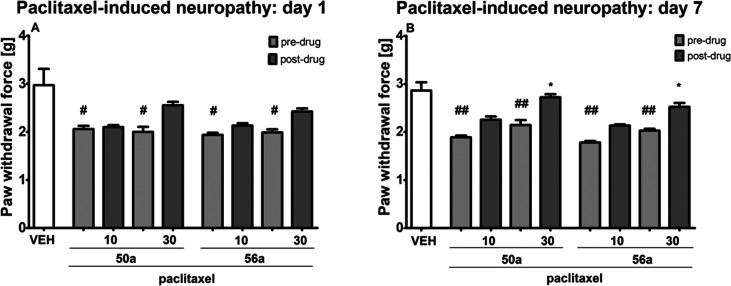
Effects of intraperitoneally administered **50a** and **56a** on the mechanical nociceptive threshold in a mouse paclitaxel-induced NP model measured using the von Frey test on the day of paclitaxel injection A) and 7 days after single-dose paclitaxel injection (B). The results are shown as the mean (±SEM) force applied to elicit paw withdrawal. Statistical analysis: one-way analysis of variance followed by Tukey’s post hoc comparison. Significance vs paw withdrawal threshold of control, nonneuropathic mice: ^#^*p* < 0.05, ^##^*p* < 0.01. Significance vs predrug (after paclitaxel) paw withdrawal threshold: *p < 0.05. In vehicle-treated mice (VEH), pain sensitivity threshold measurements were taken in the same manner and at the same time points as in the paclitaxel-treated groups, but vehicle-treated mice were not treated with paclitaxel; *n* = 8–10.

In the paclitaxel-induced NP model, the effect of **50a** and **56a** on mechanical nociceptive threshold was assessed at two time points, i.e., on the day of paclitaxel administration (4 h after paclitaxel administration) and 7 days later. On the day of paclitaxel administration, in the von Frey test an overall effect of treatment was observed for **50a** (*F*[4, 35] = 4.854, *p* < 0.01) and **56a** (*F*[4, 37] = 5.655, *p* < 0.01). The post hoc analysis revealed that compared to vehicle-treated nonneuropathic mice, paclitaxel significantly lowered mechanical nociceptive threshold in mice (*p* < 0.05). The comparison between predrug and postdrug paw withdrawal thresholds in each experimental group revealed that on the day of paclitaxel administration neither **50a** nor **56a** at doses 10 and 30 mg/kg was able to elevate the mechanical nociceptive threshold in paclitaxel-treated mice (Figure 11, panel A).

Seven days after paclitaxel administration, an overall effect of treatment was noted for **50a** and **56a** (*F*[4, 35] = 13.76, *p* < 0.0001, and *F*[4, 37] = 18.69, *p* < 0.0001, respectively). On this day of experiment vehicle-treated nonneuropathic mice had still significantly elevated mechanical nociceptive threshold as compared to paclitaxel treated mice (*p* < 0.01 vs predrug, i.e., before compound **50a** or **56a** administration, values of paw withdrawal thresholds). Of note, the comparison of predrug and postdrug paw withdrawal thresholds in **50a**-treated neuropathic mice and in **56a**-treated neuropathic mice showed that on day 7 after paclitaxel injection both compounds **50a** and **56a** at the dose of 30 mg/kg elevated mechanical nociceptive threshold (*p* < 0.05). The lower dose of **50a** or **56a** was not effective in the von Frey test (Figure 11, panel B).

#### 2.5.4 Paclitaxel-Induced Peripheral Neuropathy: Influence on Heat Nociceptive Threshold (Hot Plate Test)

**Figure 12:**
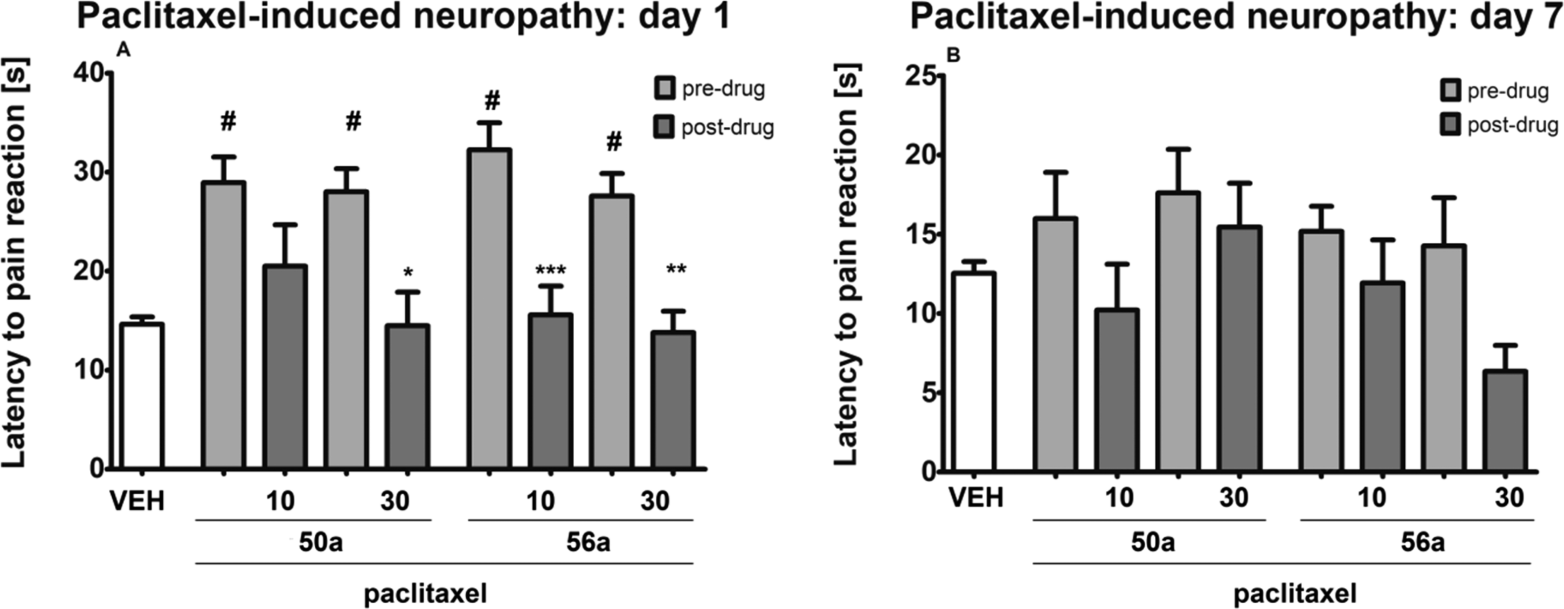
Effects of intraperitoneally administered **50a** and **56a** on the thermal (heat) pain threshold in a mouse paclitaxel-induced NP model measured using the hot plate test on the day of paclitaxel injection (A) and 7 days after paclitaxel injection (B). The results are shown as the mean (±SEM) latency to pain reaction. Statistical analysis: one-way analysis of variance followed by Tukey’s post hoc comparison. Significance vs latency of control, nonneuropathic mice: ^#^*p* < 0.05. Significance vs predrug (after paclitaxel) latency to pain reaction: *p < 0.05, **p < 0.01, *p < 0.001. In the vehicle-treated mice (VEH), measurements of the pain sensitivity threshold were taken in the same manner and at the same time points as in the paclitaxel-treated groups, but these vehicle-treated mice were not treated with paclitaxel; *n* = 8–10.

Both compounds **50a** and **56a** were also assessed for their ability to affect thermal (heat) nociceptive threshold in paclitaxel-treated mice. As shown in Figure 12 (panel A), on the day of paclitaxel administration a significantly prolonged latency to pain reaction and an increased heat nociceptive threshold were noted in all groups treated with this taxane derivative (*p* < 0.05 vs vehicle-treated nonneuropathic mice). This effect indicated that paclitaxel induced hypoalgesia in mice, which was noted on the day of paclitaxel administration but not 7 days later (Figure 12, panel A vs panel B).

On the day of paclitaxel administration, in the hot plate test, one-way ANOVA showed an overall effect of treatment for **50a** (*F*[4, 39] = 6.075, *p* < 0.001) and **56a** (*F*[4, 39] = 14.27, *p* < 0.0001). The post hoc analysis demonstrated that in paclitaxel-treated mice the compound **50a** reduced latency to pain reaction at the dose of 30 mg/kg (*p* < 0.05 vs predrug latency to pain reaction; Figure 12, panel A) and **56a** reduced latency to pain reaction at doses 10 mg/kg (*p* < 0.001 vs predrug latency to pain reaction) and 30 mg/kg (*p* < 0.01 vs predrug latency to pain reaction; Figure 12, panel A).

Seven days after paclitaxel administration, one-way ANOVA did not show an overall effect of treatment for **50a** (*F*[4,35] = 1.407, *p* > 0.05) in the hot plate test. In contrast to this, in this assay, an overall effect of treatment was noted for **56a** (*F*[4, 37] = 2.765, *p* < 0.05). At this time point of testing, Tukey’s post hoc analysis did not reveal the effect of paclitaxel and compounds **50a** and **56a** on the thermal nociceptive threshold in the hot plate test (Figure 12, panel B).

#### 2.5.5 Diabetic, STZ-Induced Peripheral Neuropathy: Influence on Tactile Allodynia (von Frey Test)

**Figure 13:**
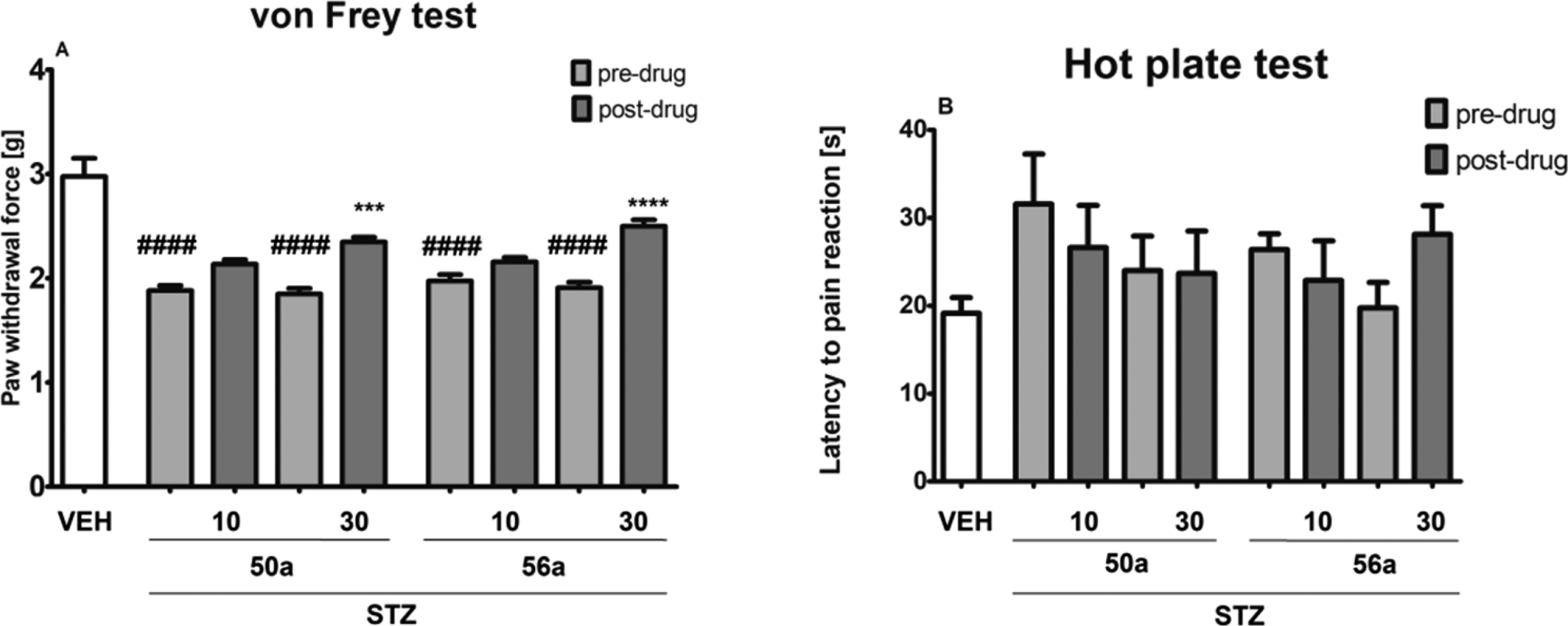
Effects of intraperitoneally administered **50a** and **56a** on the mechanical nociceptive threshold measured using the von Frey test (A) and effects of **50a** and **56a** on the thermal nociceptive threshold measured using the hot plate test (B) in a mouse STZ-induced NP model. The results are shown as the mean (±SEM) force applied to elicit paw withdrawal or the mean (±SEM) latency to pain reaction. Statistical analysis: one-way analysis of variance followed by Tukey’s post hoc comparison. Significance vs paw withdrawal threshold of control, normoglycemic (nonneuropathic) mice: ^####^*p* < 0.0001. Significance vs predrug (after STZ) paw withdrawal threshold: ***p < 0.001, ****p < 0.0001. In vehicle-treated mice (VEH), pain sensitivity threshold measurements were taken in the same manner as in the STZ-treated groups, but vehicle-treated mice were not treated with STZ; *n* = 8–10.

In the mouse model of painful diabetic neuropathy induced by STZ, an overall effect of treatment was demonstrated for both **50a** and **56a** in the von Frey test (*F*[4, 42] = 33.02, *p* < 0.0001, and *F*[4, 40] = 26.59, *p* < 0.0001, respectively). As shown in Figure 13 (panel A), STZ lowered mechanical nociceptive threshold in mice (*p* < 0.0001 vs normoglycemic control). In the von Frey test, compounds **50a** and **56a** were effective only at the dose of 30 mg/kg (**50a**, *p* < 0.001; **56a**, *p* < 0.0001 vs predrug paw withdrawal in the individual group).

#### 2.5.6 Diabetic, STZ-Induced Peripheral Neuropathy: Influence on Heat Hyperalgesia (Hot Plate Test)

In the mouse model of painful diabetic neuropathy induced by STZ, an overall effect of treatment on the heat nociceptive threshold was not demonstrated for both **50a** and **56a** in the hot plate test (*F*[4,38] = 0.9254, *p* > 0.05, and *F*[4,40] = 1.589, *p* > 0.05, respectively). As shown in Figure 13 (panel B), STZ slightly increased thermal nociceptive threshold in mice but this effect compared to that of normoglycemic control did not reach statistical significance. In the hot plate test, neither **50a** nor **56a** was effective.

#### 2.5.7 Effects on Motor Coordination (Rotarod Test)

In the rotarod test, the effect of the test compounds on motor coordination of mice was assessed. Compared to the vehicle-treated group, none of the test compounds induced motor deficits in the rotarod test.

## 3 Conclusions

Neuropathic pain is a global public health problem and is most frequently caused by chronic, progressive nerve disease after surgery or trauma and viral infections in the course of diabetes or could be induced by chemotherapy. It is worth pointing out that painful diabetic neuropathy is a major complication of diabetes and a cause of increased mortality. Unfortunately, currently used drugs have limited efficacy and patients remain refractory to existing pharmacological treatment. Hence, there is a substantial need for further development of new and effective drugs for NP therapy. Considering the above, the present work describes SAR studies of new functionalized amino acids as inhibitors of GATs, the biological targets in the search for new treatment of NP. A series of 56 novel derivatives of 3-hydroxypropanamide, 4-hydroxybutanamide, and 4-hydroxypentamide were synthesized and evaluated toward all four mouse GAT subtypes (mGAT1–4). On the basis of the obtained *in vitro* results, we selected three compounds 2*RS*,4*RS*-**39c** (pIC_50_(mGAT4) = 5.36 ± 0.10), **50a** (pIC_50_(mGAT2) = 5.43 ± 0.11), and **56a** (pIC_50_(mGAT4) = 5.04 ± 0.04) for further investigation. The obtained results indicated a negligible hepatotoxic and cytotoxic effect of the tested compounds at 0.1 and 1 μM on HepG2 and HEK-293 cells. Their safety profile was also examined in *in vivo* studies, where the tested compounds did not show a neurotoxic effect in mice at the doses displaying analgesic effect. In a set of *in vivo* experiments, two compounds, **50a** and **56a** at doses of 10 and 30 mg/kg, showed antiallodynic properties in rodent models of NP induced by oxaliplatin. Interestingly, compound **56a** at a dose of 10 mg/kg showed antiallodynic properties in the acute and late phases of oxaliplatin-induced neuropathy, and additionally at the dose of 30 mg/kg **56a** reduced cold hyperalgesia in the acute phase. In the paclitaxel model of NP both **50a** and **56a** were able to reduce tactile allodynia when administered 7 days after paclitaxel injection. This effect was noted for the dose of 30 mg/kg of both test compounds. Interestingly, in this model of NP, on the day of paclitaxel administration, both **50a** and **56a** reduced hypoalgesia induced by paclitaxel and they restored a physiological heat nociceptive threshold in paclitaxel-treated mice. The compound **56a** was more effective in reducing heat hypoalgesia than **50a**. As for the STZ model of NP, both compounds were able to reduce tactile allodynia in diabetic, neuropathic mice. Finally, compound **56a** demonstrated predominant antinociceptive properties in rodent models of NP and has provided a great contribution to the current knowledge on the importance of GABA reuptake in the pathophysiology and pharmacotherapy of NP.

## 4 Methods

### 4.1 Chemistry

Commercially available reagents were purchased from Merck, Aldrich, Acros, or ChemPur and were used without further purification. Solvents for reactions carried out under inert gas (argon), such as tetrahydrofuran (THF) and DCM, were dried, distilled, and collected under argon before use. THF was distilled from a mixture of sodium and benzophenone, while DCM was distilled from calcium hydride. Triethylamine (TEA) was distilled under vacuum before use. Reactions carried out under microwave irradiation used a Discover LabMate (CEM Corporation, USA). Purification of chemical compounds by column chromatography was carried out using Sigma-Aldrich silica gel (mesh 0.063–0.200 mm) as the stationary phase. Reactions were monitored by thin-layer chromatography (TLC) (aluminum sheets precoated with silica gel 60 F_254_ (Merck)). Compounds were visualized with UV light (254 nm). Additionally, the plates were stained with a 0.5% solution of ninhydrin in *n*-propanol or a solution of 5% (NH_4_)_6_Mo_7_O_24_ and 0.2% Ce(SO_4_)_2_ in 5% H_2_SO_4_. The retention factor (*R*_*f*_) was defined using the following solvent systems: S_1_ (petroleum ether (PE)/EtOAc 7:3, v/v), S_2_ (PE/EtOAc 1:1, v/v), S_3_ (*n-*hexane/ethanol (EtOH)/TEA 7:2:1, v/v/v), S_4_ (DCM/methanol (MeOH)/NH_3_ 9.5:0.5:0.1, v/v/v), S_5_ (NH_3_/MeOH/DCM/PE 9:45:120:18, v/v/v/v), S_6_ (DCM/acetone (Ace) 9:1, v/v), S_7_ (*n-*hexane/ethyl acetate (EtOAc) 1:1, v/v), S_8_ (DCM/MeOH, 95:5, v/v), S_9_ (chloroform (Chl)/Ace 9:1, v/v), S_10_ (DCM/EtOAc 3:2, v/v) S_11_ (Chl/Ace 1:1, v/v), S_12_ (DCM/Ace 7:3, v/v), S_13_ (25% NH_3_/MeOH/DCM/PE = 640:140:100:25, v/v/v/v). ^1^H NMR and ^13^C NMR spectra were recorded on a Varian Mercury-VX 300, with ^1^H at 300.08 MHz and ^13^C at 75.46 MHz or a JEOL ECA400II or ECX500 at magnetic field strengths of 11.75 T corresponding to ^1^H and ^13^C resonance frequencies of 500.16 and 125.77 MHz at ambient temperature (25 °C). Chemical shifts (δ) are reported in parts per million (ppm), and coupling constants (*J*) are reported in hertz (Hz). High-resolution (HR) MS was performed on a Synapt G2-S HDMS (Waters Inc.) mass spectrometer equipped with an electrospray ionization source and q-TOF type mass analyzer. The instrument was controlled, and recorded data were processed using the MassLynx v4.1 software package (Waters Inc.). Purities of the final compounds were determined with a Waters ACQUITY ultraperformance liquid chromatography (UPLC) instrument (Waters, Milford, MA, USA) coupled to a Waters TQD mass spectrometer (ESI-tandem quadrupole).

#### 4.1.1.1 General Procedure for the Synthesis of the 3-Substituted 5-Methyldihydrofuran-2(3*H*)-one or Dihydrofuran-2(3*H*)-one Derivatives (**36**, **38**, **40**, **42**, **44**, **46**) (GP1)

A mixture of anhydrous K_2_CO_3_, corresponding amine **A**, **C**, **D**, **E**, or **F** (1 equiv) and TBAB (0.18 mmol, 0.06 g, 0.01 equiv) in acetonitrile (7 mL) was stirred at 0 °C for 15 min. Then, a solution of 3-bromodihydrofuran-2(3*H*)-one (**34**) or 3-bromo-5-methyldihydrofuran-2(3*H*)-one (**35**) (1 equiv) was added dropwise, and stirring continued for 20 h at rt. After the reaction was complete, the precipitate was filtered off, and the filtrate was concentrated under vacuum. The crude product was purified by column chromatography over silica gel.

##### 4.1.1.1.1 3-{4-[Bis(3-methylthiophen-2-yl)methylidene]piperidin-1-yl}-5-methyloxolan-2-one (**36**)

According to GP1 with amine **A** (**17**) (0.86 mmol, 0.25 g, 1 equiv), 3-bromo-5-methyldihydrofuran-2(3*H*)-one (**34**) (0.86 mmol, 0.15 g, 1 equiv), TBAB (0.086 mmol, 27.7 mg, 0.1 equiv), and anhydrous K_2_CO_3_ (2.58 mmol, 0.36 g, 3 equiv) were combined in acetonitrile (7 mL). The crude product was purified by column chromatography over silica gel (DCM/Ace = 9:1) to yield **36** (260 mg, 78%, *R*_*f*_ = 0.65 (S_9_) as a yellow oil. Formula C_21_H_25_NO_2_S_2_, FW 387.56. ^1^H NMR (300 MHz, chloroform-*d*) δ ppm 1.43 (d, *J* = 5.86 Hz, 3H) 2.11 (s, 6H) 2.32–2.47 (m, 6H) 2.54–2.68 (m, 2H) 2.85 (dd, *J* = 10.84, 5.57 Hz, 2H) 3.72 (dd, *J* = 12.02, 8.50 Hz, 1H) 4.45 (dt, *J* = 10.99, 5.35 Hz, 1H) 6.77 (d, *J* = 5.28 Hz, 2 H) 7.13 (d, *J* = 4.69 Hz, 2H).

##### 4.1.1.1.2 3-((3-(10,11-Dihydro-5*H-*dibenzo[*a*,*d*][7]annulen-5-ylidene)propyl)(methyl)amino)-5-methyldihydrofuran-2(3*H*)-one (**38**)

According to GP1 with amine **C** (**21**) (5.47 mmol, 1.44 g, 1 equiv), 3-bromo-5-methyldihydrofuran-2(3*H*)-one (**34**) (5.47 mmol, 0.76 g, 1 equiv), TBAB (1.75 mmol, 0.56 g, 0.32 equiv), and anhydrous K_2_CO_3_ (5.47 mmol, 0.76 g, 1 equiv) were combined in acetonitrile (7 mL). The crude product was purified by column chromatography over silica gel (DCM/Ace = 9:1) to yield **38** (1.88 g, 95%, *R*_*f*_ = 0.74 (S_10_)) as a yellow oil. Formula C_24_H_27_NO_2_, FW 361.49. ^1^H NMR (300 MHz, chloroform-*d*) δ ppm 1.30–1.43 (m, 3H (CH*CH*_3_)) 1.61–1.88 (m, 2H (CH*CH*_2_CH)) 2.22–2.36 (m, 5H (N*CH*_3_, CH*CH*_2_CH_2_)) 2.59–2.88 (m, 3H (Ar*CH*_2_CH_2_Ar, CHCH_2_*CH*_2_)) 2.96 (br s, 1H (Ar*CH*_2_CH_2_Ar)) 3.28 (br s, 1H (Ar*CH*_2_CH_2_Ar)) 3.37 (d, *J* = 9.96 Hz, 1H (Ar*CH*_2_CH_2_Ar)) 3.60–3.70 (m, 1H (N*CH*)) 4.36 (dt, *J* = 10.99, 5.35 Hz, 1H (*CH*CH_3_)) 5.81–5.89 (m, 1H (CH*CH*_2_CH_2_)) 6.99–7.07 (m, 1H (*Ar*)) 7.08–7.23 (m, 6H (*Ar*)) 7.24–7.31 (m, 1H (*Ar*)). LCMS *m*/*z* [M + H]^+^ 362.20.

##### 4.1.1.1.3 3-[4-(9*H-*Fluoren-9-ylidene)piperidin-1-yl]-5-methyloxolan-2-one (**40**)

According to GP1 with amine **D** (**25**) (1.17 mmol, 0.29 g, 1 equiv), 3-bromo-5-methyldihydrofuran-2(3*H*)-one (**34**) (1.17 mmol, 0.21 g, 1 equiv), TBAB (0.12 mmol, 40 mg, 0.1 equiv), and anhydrous K_2_CO_3_ (3.51 mmol, 0.48 g, 3 equiv) were combined in acetonitrile (10 mL). The crude product was purified by column chromatography over silica gel (Chl/Ace = 9:1) to yield **40** (289 mg, 71%, *R*_*f*_ = 0.61 (S_9_)) as a yellow oil. Formula C_23_H_23_NO_2_, FW 361.49. ^1^H NMR (300 MHz, chloroform-*d*) δ ppm 1.30–1.41 (m, 3H) 1.98–2.54 (m, 6H) 2.88–2.98 (m, 4H) 3.43–3.59 (m, 1H) 4.40–4.52 (m, 1H) 7.19–7.45 (m, 6H (*Ar*)) 7.63–7.95 (m, 2H (*Ar*)).

##### 4.1.1.1.4 3-(4-(10,11-Dihydro-5*H-*dibenzo[*a*,*d*][7]annulen-5-ylidene)piperidin-1-yl)-5-methyldihydrofuran-2(3*H*)-one (**42**)

According to GP1 with amine **E** (**26**) (0.22 mmol, 60 mg, 1 equiv), 3-bromo-5-methyldihydrofuran-2(3*H*)-one (**34**) (0.22 mmol, 40 mg, 1 equiv), TBAB (0.022 mmol, 7 mg, 0.1 equiv), and anhydrous K_2_CO_3_ (0.65 mmol, 90 mg, 3 equiv) were combined in acetonitrile (7 mL). The crude product was purified by column chromatography over silica gel (DCM/Ace = 7:3) to yield **42** (40 mg, 49%, *R*_*f*_ = 0.46 (S_1_)) as a yellow oil. Formula C_25_H_27_NO_2_, FW 373.49. ^1^H NMR (300 MHz, chloroform-*d*) δ ppm 1.70–1.85 (m, 2H) 2.15 (s, 4H) 2.34–2.54 (m, 4H) 2.76–2.93 (m, 4H) 3.47–3.57 (m, 1H) 3.62–3.76 (m, 3H) 4.30–4.42 (m, 1H) 7.07–7.27 (m, 8H).

##### 4.1.1.1.5 3-[(2-{[(Diphenylmethylidene)amino]oxy}ethyl)(methyl)amino]-5-methyloxolan-2-one (**44**)

According to GP1 with amine **F** (**32**) (2.16 mmol, 0.55 g, 1 equiv), 3-bromo-5-methyldihydrofuran-2(3*H*)-one (**34**) (2.16 mmol, 0.38 g, 1 equiv), TBAB (0.22 mmol, 69.5 mg, 0.1 equiv), and anhydrous K_2_CO_3_ (6.48 mmol, 0.90 g, 3 equiv) were combined in acetonitrile (10 mL). The crude product was purified by column chromatography over silica gel (DCM/EtOAc = 3:2) to yield **44** (500 mg, 66%, *R*_*f*_ = 0.71 (S_10_)) as a yellow oil. Formula C_21_H_24_N_2_O_3_, FW 352.43. ^1^H NMR (300 MHz, chloroform-*d*) δ ppm 1.34 (d, *J* = 6.16 Hz, 3H (CH*CH*_3_)) 1.67 (td, *J* = 12.31, 10.52 Hz, 1H (CH*CH*_2_CH)) 2.07–2.14 (m, 1H (CH*CH*_2_CH)) 2.38 (s, 3H (N*CH*_3_)) 2.85–3.03 (m, 2H (OCH_2_*CH*_2_N)) 3.67–3.79 (m, 1H (N*CH*)) 4.06–4.23 (m, 1H (CH*CH*_3_)) 4.29–4.36 (m, 2H (O*CH*_2_CH_2_N)) 7.27–7.50 (m, 10H (*Ar*)).

##### 4.1.1.1.6 3-[(2-{[(Diphenylmethylidene)amino]oxy}ethyl)(methyl)amino]oxolan-2-one (**46**)

According to GP1 with amine **F** (**32**) (2.95 mmol, 0.75 g, 1 equiv), 3-bromodihydrofuran-2(3*H*)-one (**35**) (2.95 mmol, 0.52 g, 1 equiv), TBAB (0.30 mmol, 93.2 mg, 0.1 equiv), and anhydrous K_2_CO_3_ (8.85 mmol, 1.23 g, 3 equiv) were combined in acetonitrile (10 mL). The crude product was purified by column chromatography over silica gel (DCM/Ace = 7:3) to yield **46** (520 mg, 52%, *R*_*f*_ = 0.65 (S_1_)) as a yellow oil. Formula C_20_H_22_N_2_O_3_, FW 338.40. ^1^H NMR (300 MHz, chloroform-*d*) δ ppm 2.26–2.31 (m, 1H (CH*CH*_2_)) 2.35–2.55 (m, 4H *(*Me; CH*CH*_2_)) 2.63–2.74 (m, 2H (*CH*_2_N)) 3.49–3.56 (m, 1H (N*CH*)) 3.64–3.71 (m, 2H (=NO*CH*_2_)) 4.25–4.33 (m, 1H (O*CH*_2_)) 4.35–4.45 (m, 1H (O*CH*_2_)) 7.45–7.62 (m, 10H (Ar)).

#### 4.1.1.2 General Procedure for the Synthesis of the 4-Hydroxypentanamide and 4-Hydroxybutanamide Derivatives **37a**–**c**, **39a**–**c**, **41**, and **43a**,**b** (GP2)

The corresponding 3-substituted 5-methyldihydrofuran-2(3*H*)-one or dihydrofuran-2(3*H*)-one derivative (1 equiv), relevant 1-phenylmethanamine hydrochloride derivative (1.5 equiv), and sodium 2-ethylhexanoate (2.5 equiv) were dissolved in dry THF under Ar and stirred at 66 °C for 16 h. Then, the mixture was cooled to rt, and EtOAc (5 mL/1 mmol) and 30% K_2_CO_3_ (5 mL/1 mmol) were added. The mixture was stirred at rt for 15 min and was extracted with EtOAc (2 × 5 mL). The combined organic phases were dried over Na_2_SO_4_ and concentrated under reduced pressure. The crude product was purified by column chromatography.

##### 4.1.1.2.1 *N-*Benzyl-2-{4-[bis(3-methylthiophen-2-yl)methylidene]piperidin-1-yl}-4-hydroxypentanamide ((2*RS*,4*RS*)*-***37a** and 2*RS*,4*SR*)*-***37a**)

Compounds (2*RS*,4*RS*)*-***37a** and (2*RS*,4*SR*)*-***37a** were prepared according to GP2 with 3-{4-[bis(3-methylthiophen-2-yl)methylidene]piperidin-1-yl}-5-methyloxolan-2-one (**36**) (0.67 mmol, 0.26 g), 1-phenylmethanamine hydrochloride (1 mmol, 0.14 g), and sodium 2-ethylhexanoate (1.68 mmol, 0.28 g) in 4 mL of dry THF. The obtained crude products were purified by column chromatography over silica gel (DCM/Ace = 9:1) to yield (2*RS*,4*RS*)*-***37a** (180 mg, 54%, *R*_*f*_ = 0.56 (S_6_) and (2*RS*,4*SR*)*-***37a** (80 mg, 24%, *R*_*f*_ = 0.33 (S_6_)) as yellow oil. ds: (2*RS*,4*RS*)*-***37a/**(2*RS*,4*SR*)*-***37a** = 69:31. Formula C_28_H_34_N_2_O_2_S_2_, FW 494.71. Compound (2*RS*,4*RS*)*-***37a**^1^H NMR (300 MHz, chloroform-*d*) δ ppm 7.92–8.03 (m, 1H), 7.18–7.42 (m, 5H), 7.12 (d, *J* = 5.27 Hz, 1H), 6.76 (d, *J* = 5.28 Hz, 1H), 6.56–6.70 (m, 1H), 6.48–6.60 (m, 1H), 5.33–5.45 (m, 1H), 4.44 (s, 2H), 3.69–3.99 (m, 1H), 2.43–2.65 (m, 1H), 2.15–2.40 (m, 2H), 1.98 (ddd, *J* = 4.98, 9.82, 14.51 Hz, 1H), 1.71–1.93 (m, 1H), 1.40–1.66 (m, 4H), 1.14–1.32 (m, 6H), 0.76–0.96 (m, 5H). ^13^C NMR (75 MHz, chloroform-*d*) δ ppm 175.6, 175.6, 170.4, 137.6, 137.5, 133.8, 129.4, 128.8, 128.8, 128.7. 127.7, 127.6, 127.5, 124.0, 71.6, 64.1, 50.5, 47.3, 47.2, 43.5, 43.3, 41.8, 31.9, 31.6, 29.6, 29.5, 25.4, 25.4, 24.5, 23.6, 22.5, 14.6, 13.9, 13.8, 11.8. HRMS-ESI^+^*m*/*z* [M + H]^+^ calcd for C_28_H_34_N_2_O_2_S_2_, 495.2134; found, 495.2133. Compound (2*RS*,4*SR*)*-***37a**^1^H NMR (300 MHz, chloroform-*d*) δ ppm 7.21–7.41 (m, 8H), 7.13 (d, *J* = 5.28 Hz, 1H), 6.77 (d, *J* = 5.27 Hz, 1H), 4.42–4.55 (m, 2H), 3.39–3.57 (m, 1H), 2.50–2.68 (m, 2H), 2.30 (br s, 3H), 2.08 (s, 6H), 1.64–1.90 (m, 2H), 1.09–1.35 (m, 6H), 0.76–0.92 (m, 1H). ^13^C NMR (75 MHz, chloroform-*d*) δ ppm 175.5, 175.5, 170.3, 137.5, 137.4, 133.6, 129.2, 128.9, 128.9, 128.1, 127.6, 127.5, 127.4, 124.0, 71.6, 64.1, 50.4, 47.2, 47.1, 43.5, 43.2, 41.7, 31.8, 31.7, 29.7, 29.6, 25.5, 25.3, 24.6, 23.6, 22.8, 14.5, 13.8, 13.8, 11.7. HRMS-ESI^+^*m*/*z* [M + H]^+^ calcd for C_28_H_34_N_2_O_2_S_2_, 495.2134; found, 495.2139.

##### 4.1.1.2.2 2-{4-[Bis(3-methylthiophen-2-yl)methylidene]piperidin-1-yl}-*N-*[(2-chlorophenyl)methyl]-4-hydroxypentanamide ((2*RS*,4*RS*)*-***37b** and (2*RS*,4*SR*)*-***37b**)

Compounds (2*RS*,4*RS*)*-***37b** and (2*RS*,4*SR*)*-***37b** were prepared according to GP2 with 3-{4-[bis(3-methylthiophen-2-yl)methylidene]piperidin-1-yl}-5-methyloxolan-2-one (**36**) (2.12 mmol, 0.83 g), 1-(2-chlorophenyl)methanamine hydrochloride (3.18 mmol, 0.56 g), and sodium 2-ethylhexanoate (5.34 mmol, 0.95 g) in 10 mL of dry THF. The obtained crude products were purified by column chromatography over silica gel (DCM/Ace = 9:1) to yield (2*RS*,4*RS*)*-***37b** (533 mg, 47%, *R*_*f*_ = 0.58 (S_6_)) and (2*RS*,4*SR*)*-***37b** (238 mg, 21%, *R*_*f*_ = 0.35 (S_6_)) as a yellow oil. ds: (2*RS*,4*RS*)*-***37b/**(2*RS*,4*SR*)*-***37b** = 69:31. Formula C_28_H_33_ClN_2_O_2_S_2_, FW 529.15. Compound (2*RS*,4*RS*)*-***37b**: ^1^H NMR (300 MHz, chloroform-*d*) δ ppm 7.58–7.68 (m, 1H), 7.35–7.43 (m, 2H), 7.21–7.28 (m, 2H), 7.11–7.16 (m, 2H), 6.76–6.80 (m, 2H), 4.36–4.64 (m, 3H), 3.98–4.07 (m, 1H), 3.31–3.40 (m, 1H), 2.54 (d, *J* = 4.11 Hz, 4H), 2.26–2.37 (m, 4H), 2.10 (s, 6H), 1.74 (t, *J* = 6.16 Hz, 2H), 1.18 (d, *J* = 6.46 Hz, 3H). ^13^C NMR (75 MHz, chloroform-*d*) δ ppm 173.1, 143.1, 137.9, 135.5, 133.8, 133.7, 130.5, 129.7, 129.5, 129.3, 127.3, 123.9, 120.2, 65.5, 65.2, 51.0, 41.6, 34.2, 32.0, 22.9, 14.7. HRMS-ESI^+^*m*/*z* [M + H]^+^ calcd for C_28_H_33_ClN_2_O_2_S_2_, 529.1745; found, 529.1747. Compound (2*RS*,4*SR*)*-***37b**: ^1^H NMR (300 MHz, chloroform-*d*) δ ppm 7.57–7.67 (m, 1H), 7.34–7.42 (m, 2H), 7.20–7.27 (m, 2H), 7.10–7.15 (m, 2H), 6.75–6.79 (m, 2H), 4.35–4.63 (m, 3H), 3.97–4.06 (m, 1H), 3.30–3.39 (m, 1H), 2.53 (d, *J* = 4.10 Hz, 4H), 2.25–2.36 (m, 4H), 2.09 (s, 6H), 1.73 (t, *J* = 6.15 Hz, 2H), 1.17 (d, *J* = 6.45 Hz, 3H). ^13^C NMR (75 MHz, chloroform-*d*) δ ppm 173.0, 143.1, 137.8, 135.5, 133.7, 133.6, 130.5, 129.6, 129.4, 129.2, 127.2, 123.9, 120.1, 65.4, 65.1, 51.0, 41.5, 34.2, 32.0, 22.9, 14.6. HRMS-ESI^+^*m*/*z* [M + H]^+^ calcd for C_28_H_33_ClN_2_O_2_S_2_, 529.1745; found, 529.1747.

##### 4.1.1.2.3 2-{4-[Bis(3-methylthiophen-2-yl)methylidene]piperidin-1-yl}-*N-*[(4-fluorophenyl)methyl]-4-hydroxypentanamide ((2*RS*,4*RS*)*-***37c** and (2*RS*,4*SR*)*-***37c**)

Compounds (2*RS*,4*RS*)*-***37c** and (2*RS*,4*SR*)*-***37c** were prepared according to GP2 with 3-{4-[bis(3-methylthiophen-2-yl)methylidene]piperidin-1-yl}-5-methyloxolan-2-one (**36**) (2.12 mmol, 0.83 g), 1-(4-fluorophenyl)methanamine hydrochloride (3.18 mmol, 0.51 g), and sodium 2-ethylhexanoate (5.34 mmol, 0.95 g) in 10 mL of dry THF. The obtained crude products were purified by column chromatography over silica gel (DCM/Ace = 9:1) to yield (2*RS*,4*RS*)*-***37c** (527 mg, 48%, *R*_*f*_ = 0.55 (DCM/Ace = 9:1)) and (2*RS*,4*SR*)*-***37c** (230 mg, 21%, *R*_*f*_ = 0.37 (DCM/Ace = 9:1)) as a yellow oil. ds: (2*RS*,4*RS*)*-***23a/**(2*RS*,4*SR*)*-***37c** = 70:30. Formula C_28_H_33_FN_2_O_2_S_2_, FW 512.70. Compound (2*RS*,4*RS*)*-***37c**: ^1^H NMR (300 MHz, chloroform-*d*) δ ppm 7.50–7.59 (m, 1H), 7.20–7.29 (m, 2H), 7.12 (d, *J* = 5.29 Hz, 2H), 7.01 (s, 2H), 6.77 (d, *J* = 5.29 Hz, 2H), 4.28–4.54 (m, 3H), 3.97–4.08 (m, 1H), 3.34–3.43 (m, 1H), 2.50–2.66 (m, 4H), 2.24–2.38 (m, 4H), 2.09 (s, 6H), 1.71–1.81 (m, 2H), 1.13–1.21 (m, 3H). ^13^C NMR (75 MHz, chloroform-*d*) δ ppm 172.8, 163.8, 160.6, 143.0, 137.8, 134.3, 134.3, 133.8, 129.4, 129.4, 129.3, 124.1, 120.4, 115.8, 115.6, 65.7, 65.3, 51.1, 42.5, 34.5, 31.9, 31.7, 29.8, 23.2, 14.7. HRMS-ESI^+^*m*/*z* [M + H]^+^ calcd for C_28_H_33_FN_2_O_2_S_2_, 513.2040; found, 513.2045. Compound (2*RS*,4*SR*)*-***37c**: ^1^H NMR (300 MHz, chloroform-*d*) δ ppm 7.49–7.58 (m, 1H), 7.19–7.28 (m, 2H), 7.11 (d, *J* = 5.28 Hz, 2H), 7.00 (s, 2H), 6.76 (d, *J* = 5.28 Hz, 2H), 4.27–4.53 (m, 3H), 3.96–4.07 (m, 1H), 3.33–3.42 (m, 1H), 2.49–2.65 (m, 4H), 2.23–2.37 (m, 4H), 2.08 (s, 6H), 1.70–1.80 (m, 2H), 1.12–1.20 (m, 3H). ^13^C NMR (75 MHz, chloroform-*d*) δ ppm 172.7, 163.7, 160.5, 143.0, 137.7, 134.2, 134.2, 133.7, 129.4, 129.4, 129.3, 124.0, 120.3, 115.7, 115.5, 65.6, 65.3, 51.0, 42.5, 34.4, 31.9, 31.7, 29.7, 23.1, 14.6. HRMS-ESI^+^*m*/*z* [M + H]^+^ calcd for C_28_H_33_FN_2_O_2_S_2_, 513.2040; found, 513.2045.

##### 4.1.1.2.4 2-((3-(10,11-Dihydro-5*H-*dibenzo[*a*,*d*][7]annulen-5-ylidene)propyl)(methyl)amino)-*N-*(4-fluorobenzyl)-4-hydroxypentanamide ((2*RS*,4*RS*)*-***39a**)

Compound (2*RS*,4*RS*)*-***39a** was prepared according to GP2 with 3-((3-(10,11-dihydro-5*H-*dibenzo[*a*,*d*][7]annulen-5-ylidene)propyl)(methyl)amino)-5-methyldihydrofuran-2(3*H*)-one (**38**) (1.66 mmol, 0.60 g), 1-(4-fluorophenyl)methanamine hydrochloride (2.49 mmol, 0.31 g) and sodium 2-ethylhexanoate (4.15 mmol, 0.69 g) in 4 mL of dry THF. The obtained crude product was purified by column chromatography over silica gel (DCM/Ace = 9:1) to yield (2*RS*,4*RS*)*-***39a** (249 mg, 31%, *R*_*f*_ = 0.74 (DCM/Ace = 7:3) as a yellow oil. Formula C_31_H_35_FN_2_O_2_, FW 486.27.^1^H NMR (300 MHz, chloroform-*d*) δ ppm 1.18–1.34 (m, 4H (C*CH*_3_)) 1.59–1.79 (m, 2H (CH*CH*_2_CH)) 2.03 (br s, 3H (N*CH*_3_)) 2.18–2.35 (m, 2H (CH*CH*_2_CH_2_)) 2.44 (br s, 2H (CHCH_2_*CH*_2_)) 2.76 (br s, 1H (Ar*CH*_2_CH_2_Ar)) 2.86–3.08 (m, 1H (Ar*CH*_2_CH_2_Ar)) 3.08–3.37 (m, 3H (Ar*CH*_2_CH_2_Ar, N*CH*)) 3.63–3.76 (m, 1H (*CH*OH)) 4.06–4.38 (m, 2H (NH*CH*_2_)) 5.70 (br s, 1H (*CH*CH_2_CH_2_)) 6.94 (br s, 3H (*Ar*)) 6.99–7.09 (m, 3H (*Ar*)) 7.10–7.24 (m, 6H (*Ar*)) 7.97 (br s, 1H (*NH*CH_2_)). ^13^C NMR (75 MHz, chloroform-*d*) δ ppm 24.61, 31.96, 33.69, 42.51, 54.38, 66.99, 67.48, 115.29, 115.58, 125.78, 126.13, 127.27, 127.65, 128.10, 128.42, 129.22, 129.33, 130.20, 133.86, 133.91, 139.71, 163.70, 174.81. LCMS *m*/*z* [M + H]^+^ = 487.22. HRMS-ESI^+^*m*/*z* [M + H]^+^ calcd for C_31_H_35_FN_2_O_2_, 487.2755; found, 487.2758.

##### 4.1.1.2.5 *N*-(4-Chlorobenzyl)-2-((3-(10,11-dihydro-5*H-*dibenzo[*a*,*d*][7]annulen-5-ylidene)propyl)(methyl)amino)-4-hydroxypentanamide ((2*RS*,4*RS*)*-***39b** and (2*RS*,4*SR*)*-***39b**)

Compounds (2*RS*,4*RS*)*-***39b** and (2*RS*,4*SR*)*-***39b** were prepared according to GP2 with 3-((3-(10,11-dihydro-5*H-*dibenzo[*a*,*d*][7]annulen-5-ylidene)propyl)(methyl)amino)-5-methyldihydrofuran-2(3*H*)-one (**38**) (1.66 mmol, 0.60 g), 1-(4-chlorophenyl)methanamine hydrochloride (2.73 mmol, 0.43 g), and sodium 2-ethylhexanoate (4.55 mmol, 0.76 g) in 4 mL of dry THF. The obtained crude products were purified by column chromatography over silica gel (DCM/Ace = 9:1) to yield (2*RS*,4*RS*)*-***39a** (358 mg, 41%, *R*_*f*_ = 0.33 (DCM/Ace = 9:1)) and (2*RS*,4*SR*)*-***39b** (114 mg, 13%, *R*_*f*_ = 0.18 (DCM/Ace = 9:1)) as a yellow oil. ds: (2*RS*,4*RS*)*-***39a/**(2*RS*,4*SR*)*-***39a** = 76:24. Formula C_31_H_35_ClN_2_O_2_, FW 503.08. Compound (2*RS*,4*RS*)*-***39b**: ^1^H NMR (300 MHz, chloroform-*d*) δ ppm 1.20–1.27 (m, 2H (CHC*H*_3_)) 1.46–1.78 (m, 2H (CHC*H*_2_CH)) 2.03 (br s, 3H (NC*H*_3_)) 2.20–2.33 (m, 2H (CHC*H*_2_CH_2_)) 2.42 (br s, 2H (CHCH_2_C*H*_2_)) 2.75 (br s, 1H (ArC*H*_2_CH_2_Ar)) 2.93 (br s, 1H (ArC*H*_2_CH_2_Ar)) 3.16 (d, *J* = 13.48 Hz, 1H (ArC*H*_2_CH_2_Ar)) 3.27 (br s, 2H (ArC*H*_2_CH_2_Ar, NC*H*)) 3.65–3.77 (m, 1H (C*H*OH)) 4.06–4.40 (m, 2H (NHC*H*_2_)) 5.69 (br s, 1H (C*H*CH_2_CH)) 6.93 (br s, 1H (*Ar*)) 6.98–7.26 (m, 12H (*Ar*)) 7.99 (br s, 1H (N*H*CH_2_)). ^13^C NMR (75 MHz, chloroform-*d*) δ ppm 22.67, 24.56, 31.98, 33.70, 38.42, 42.52, 47.18, 53.83, 66.99, 67.43, 121.78, 125.79, 126.14, 127.29, 127.67, 128.05, 128.11, 128.42, 128.72, 128.97, 130.23, 136.61, 136.90, 139.68, 174.86. LCMS *m*/*z* [M + H]^+^ = 503.23. HRMS-ESI^+^*m*/*z* [M + H]^+^ calcd for C_31_H_35_ClN_2_O_2_, 503.2460; found, 503.2462. Compound (2*RS*,4*SR*)*-***39b**: ^1^H NMR (300 MHz, chloroform-*d*) δ ppm 1.14 (d, *J* = 6.45 Hz, 3H (CHC*H*_3_)) 1.60 (ddd, *J* = 13.63, 6.59, 3.22 Hz, 1H (CHC*H*_2_CH)) 1.83 (br s, 1H (CHC*H*_2_CH)) 2.05 (s, 3H (NC*H*_3_)) 2.26 (br s, 2H (CHC*H*_2_CH_2_)) 2.46 (br s, 2H (CHCH_2_C*H*_2_)) 2.75 (br s, 1H (ArC*H*_2_CH_2_Ar)) 2.88–3.04 (m, 1H (ArC*H*_2_CH_2_Ar)) 3.17 (br s, 1H (ArC*H*_2_CH_2_Ar)) 3.35 (dd, *J* = 9.38, 2.93 Hz, 2H (ArC*H*_2_CH_2_Ar, NC*H*)) 4.04 (br s, 1H (C*H*OH)) 4.13–4.40 (m, 2H (NHC*H*_2_)) 5.74 (br s, 1H (C*H*CH_2_CH_2_)) 6.91–7.24 (m, 12H (*Ar*)) 7.69 (br s, 1H (N*H*CH_2_)). ^13^C NMR (75 MHz, chloroform-*d*) δ ppm 22.82, 27.86, 29.28, 31.98, 33.72, 42.46, 64.88, 125.79, 126.13, 127.26, 127.62, 128.02, 128.17, 128.45, 128.74, 129.01, 130.19, 136.92, 139.77, 174.19. LCMS *m*/*z* [M + H]^+^ = 503.23. HRMS-ESI^+^*m*/*z* [M + H]^+^ calcd for C_31_H_35_ClN_2_O_2_, 503.2460; found, 503.2462.

##### 4.1.1.2.6 2-((3-(10,11-Dihydro-5*H-*dibenzo[*a*,*d*][7]annulen-5-ylidene)propyl)(methyl)amino)-4-hydroxy-*N*-(4-methylbenzyl)pentanamide ((2*RS*,4*RS*)*-***39c** and (2*RS*,4*SR*)*-***39c**)

Compounds (2*RS*,4*RS*)*-***39c** and (2*RS*,4*SR*)-**39c** were prepared according to GP2 with 3-((3-(10,11-dihydro-5*H-*dibenzo[*a*,*d*][7]annulen-5-ylidene)propyl)(methyl)amino)-5-methyldihydrofuran-2(3*H*)-one (**38**) (1.66 mmol, 0.60 g), 1-(4-methylphenyl)methanamine hydrochloride (2.73 mmol, 0.43 g), and sodium 2-ethylhexanoate (2.73 mmol, 0.43 g) in 4 mL of dry THF. The obtained crude products were purified by column chromatography over silica gel (DCM/Ace = 9:1) to yield (2*RS*,4*RS*)*-***39c** (358 mg, 41%, *R*_*f*_ = 0.39 (DCM/Ace = 9:1)) and (2*RS*,4*SR*)*-***39c** (114 mg, 13%, *R*_*f*_ = 0.32 (DCM/Ace = 9:1)) as a yellow oil. ds: (2*RS*,4*RS*)*-***39c/**(2*RS*,4*SR*)*-***39c** = 76:24. Formula C_32_H_38_N_2_O_2_, FW 482.67. Compound (2*RS*,4*RS*)*-***39c**: ^1^H NMR (300 MHz, chloroform-*d*) δ ppm 1.21–1.27 (m, 3H (CH*CH*_3_)) 1.64–1.73 (m, 2H (CH*CH*_2_CH)) 2.05 (s, 3H (Ar*CH*_3_)) 2.18–2.29 (m, 2H (CH*CH*_2_CH_2_)) 2.32 (s, 3H (N*CH*_3_)) 2.44 (br s, 2H (CH*CH*_2_CH_2_)) 2.75 (br s, 1H (Ar*CH*_2_CH_2_Ar)) 2.94 (br s, 1H (Ar*CH*_2_CH_2_Ar)) 3.12–3.39 (m, 3H (Ar*CH*_2_CH_2_Ar, N*CH*)) 3.67–3.74 (m, 1H (CHOH)) 4.13–4.40 (m, 2H (NHCH_2_)) 5.72 (br s, 1H (*CH*CH_2_CH_2_)) 6.91–7.24 (m, 12H (*Ar*)) 7.87 (br s, 1H (*NH*CH_2_)). ^13^C NMR (75 MHz, chloroform-*d*) δ ppm 21.11, 24.67, 28.04, 31.99, 33.72, 43.03, 66.90, 67.48, 125.78, 126.10, 127.61, 128.07, 128.45, 129.32, 130.17, 135.02, 136.93, 139.30, 139.79, 174.64. LCMS *m*/*z* [M + H]^+^ = 483.29. HRMS-ESI^+^*m*/*z* [M + H]^+^ calcd for C_31_H_35_ClN_2_O_2_, 483.3006; found, 483.3003. Compound (2*RS*,4*SR*)*-***23a**: ^1^H NMR (300 MHz, chloroform-*d*) δ ppm 1.13 (d, *J* = 6.45 Hz, 3H (CH*CH*_3_)) 1.61 (ddd, *J* = 14.07, 7.03, 3.52 Hz, 1H (CH*CH*_2_CH)) 1.79–1.89 (m, 1H (CH*CH*_2_CH)) 2.07 (s, 3H (Ar*CH*_3_)) 2.21–2.29 (m, 2H (CH*CH*_2_CH_2_))) 2.32 (s, 3H (N*CH*_3_)) 2.42–2.50 (m, 2H (CH*CH*_2_CH_2_)) 2.75 (br s, 1H (Ar*CH*_2_CH_2_Ar)) 2.94 (br s, 1H (Ar*CH*_2_CH_2_Ar)) 3.20 (br s, 1H (N*CH*)) 3.33 (dd, *J* = 9.38, 3.52 Hz, 2H (Ar*CH*_2_CH_2_Ar)) 4.00–4.08 (m, 1H (*CH*OH)) 4.22–4.37 (m, 2H (NHCH_2_)) 5.76 (t, *J* = 7.03 Hz, 1H (*CH*CH_2_CH_2_)) 6.94–7.24 (m, 12H (*Ar*)) 7.50 (br s, 1H (NHCH_2_)). ^13^C NMR (75 MHz, chloroform-*d*) δ ppm 21.10, 22.75, 27.95, 29.28, 31.96, 33.70, 42.94, 64.90, 125.78, 126.07, 127.16, 127.56, 127.65, 128.00, 128.11, 128.46, 129.30, 130.13, 135.19, 136.95, 139.85, 173.94. LCMS *m*/*z* [M + H]^+^ = 483.29. HRMS-ESI^+^*m*/*z* [M + H]^+^ calcd for C_31_H_35_ClN_2_O_2_, 483.3006; found, 483.3003.

##### 4.1.1.2.7 *N-*Benzyl-2-[4-(9*H-*fluoren-9-ylidene)piperidin-1-yl]-4-hydroxypentanamide ((2*RS*,4*RS*)*-***41** and (2*RS*,4*SR*)*-***41**)

Compounds (2*RS*,4*RS*)*-***41** and (2*RS*,4*SR*)*-***41** were prepared according to GP2 with 3-[4-(9*H-*fluoren-9-ylidene)piperidin-1-yl]-5-methyloxolan-2-one (**40**) (0.59 mmol, 0.23 g), 1-phenylmethanamine hydrochloride (0.89 mmol, 0.19 g), and sodium 2-ethylhexanoate (1.45 mmol, 0.285 g) in 4 mL of dry THF. The obtained crude products were purified by column chromatography over silica gel (DCM/Ace = 9:1) to yield (2*RS*,4*RS*)*-***41** (150 mg, 50%, *R*_*f*_ = 0.48 (DCM/Ace = 9:1)) and (2*RS*,4*SR*)*-***41** (51 mg, 17%, *R*_*f*_ = 0.29 (DCM/Ace = 9:1)) as a yellow oil. ds: (2*RS*,4*RS*)*-***41/**(2*RS*,4*SR*)*-***41** = 75:25. Formula C_30_H_32_N_2_O_2_, FW 452.59. Compound (2*RS*,4*RS*)*-***41**: ^1^H NMR (300 MHz, chloroform-*d*) δ ppm 1.17–1.31 (m, 3H) 1.69–1.89 (m, 2H) 1.96–2.57 (m, 2H) 2.70–2.87 (m, 2H) 3.08–3.44 (m, 4H) 3.70–3.88 (m, 1H) 4.19–4.65 (m, 3H) 7.04–7.46 (m, 10H) 7.69–7.86 (m, 3H) 7.99 (br s, 1H). Compound (2*RS*,4*SR*)*-***41**: ^1^H NMR (300 MHz, chloroform-*d*) δ ppm 1.14–1.24 (m, 3H) 1.68–1.88 (m, 2H) 2.38–2.54 (m, 1H) 2.67–2.89 (m, 3H) 2.95 (br s, 2H) 3.18–3.25 (m, 2H) 3.26–3.53 (m, 2H) 3.96–4.10 (m, 1H) 4.40–4.45 (m, 1H) 4.51 (s, 1H) 7.22–7.41 (m, 11H) 7.71–7.86 (m, 3H).

##### 4.1.1.2.8 *N*-Benzyl-2-(4-(10,11-dihydro-5*H*-dibenzo[*a*,*d*][7]annulen-5-ylidene)piperidin-1-yl)-4-hydroxypentanamide ((2*RS*,4*RS*)*-***43a** and (2*RS*,4*SR*)*-***43a**)

Compounds (2*RS*,4*RS*)*-***43a** and (2*RS*,4*SR*)*-***43a** were prepared according to GP2 with 3-(4-(10,11-dihydro-5*H-*dibenzo[*a*,*d*][7]annulen-5-ylidene)piperidin-1-yl)-5-methyldihydrofuran-2(3*H*)-one (**42**) (0.48 mmol, 0.18 g), 1-phenylmethanamine hydrochloride (0.72 mmol, 0.10 g), and sodium 2-ethylhexanoate (1.80 mmol, 0.30 g) in 4 mL of dry THF. The obtained crude products were purified by column chromatography over silica gel (DCM/Ace = 9:1) to yield (2*RS*,4*RS*)*-***43a** (120 mg, 52%, *R*_*f*_ = 0.48 (DCM/Ace = 9:1)) and (2*RS*,4*SR*)*-***43a** (30 mg, 13%, *R*_*f*_ = 0.29 (DCM/Ace = 9:1)) as a yellow oil. ds: (2*RS*,4*RS*)*-***43a/**(2*RS*,4*SR*)*-***43a** = 80:20. Formula: C_32_H_36_N_2_O_2_, FW 480.65. Compound (2*RS*,4*RS*)*-***43a**: ^1^H NMR (300 MHz, chloroform-*d*) δ ppm 8.02 (t, *J* = 5.86 Hz, 1H), 7.21–7.46 (m, 5H), 6.99–7.20 (m, 8H), 4.35–4.62 (m, 2H), 3.72–3.87 (m, 1H), 3.28–3.46 (m, 3H), 2.76–2.93 (m, 2H), 2.57–2.69 (m, 2H), 2.39–2.55 (m, 3H), 2.28–2.39 (m, 3H), 1.69–1.87 (m, 2H), 1.28 (d, *J* = 5.86 Hz, 3H). ^13^C NMR (75 MHz, chloroform-*d*) δ ppm 174.3, 150.0, 140.4, 138.1, 138.1, 137.9, 135.7, 132.7, 129.4, 129.3, 128.8, 128.7, 127.8, 127.6, 127.5, 127.0, 126.9, 125.8, 125.5, 67.6, 67.4, 53.9, 51.8, 50.3, 43.5, 33.6, 32.5, 31.3, 31.3, 29.7, 29.3, 24.6. HRMS-ESI^+^*m*/*z* [M + H]^+^ calcd for C_32_H_37_N_2_O_2_, 481.2855; found, 481.2855. Compound (2*RS*,4*SR*)*-***43a**: ^1^H NMR (300 MHz, chloroform-*d*) δ ppm 7.42 (t, *J* = 5.57 Hz, 1H), 7.24–7.39 (m, 5H), 7.06–7.16 (m, 6H), 6.99–7.06 (m, 2H), 4.47 (dd, *J* = 6.15, 14.95 Hz, 2H), 3.99–4.11 (m, 1H), 3.28–3.45 (m, 3H), 2.75–2.89 (m, 2H), 2.67 (dd, *J* = 4.40, 14.95 Hz, 2H), 2.29–2.44 (m, 6H), 1.69–1.82 (m, 2H), 1.18 (d, *J* = 6.45 Hz, 3H). ^13^C NMR (75 MHz, chloroform-*d*) δ ppm 173.0, 140.5, 140.4, 138.2, 137.9, 135.5, 133.0, 129.3, 128.8, 128.7, 127.6, 127.6, 126.9, 125.5, 65.5, 65.3, 51.9, 51.1, 43.3, 34.2, 32.4, 31.2, 22.9.

##### 4.1.1.2.9 2-(4-(10,11-Dihydro-5*H-*dibenzo[*a*,*d*][7]annulen-5-ylidene)piperidin-1-yl)-*N-*(4-fluorobenzyl)-4-hydroxypentanamide ((2*RS*,4*RS*)*-***43b** and (2*RS*,4*SR*)*-***43b**)

Compounds (2*RS*,4*RS*)*-***43b** and (2*RS*,4*SR*)*-***43b** were prepared according to GP2 with 3-(4-(10,11-dihydro-5*H-*dibenzo[*a*,*d*][7]annulen-5-ylidene)piperidin-1-yl)-5-methyldihydrofuran-2(3*H*)-one (**42**) (0.63 mmol, 0.24 g), 1-(4-fluorophenyl)methanamine hydrochloride (0.95 mmol, 0.15 g), and sodium 2-ethylhexanoate (1.60 mmol, 0.27 g) in 4 mL of dry THF. The obtained crude products were purified by column chromatography over silica gel (DCM/Ace = 9:1) to yield (2*RS*,4*RS*)*-***43b** (120 mg, 38%, *R*_*f*_ = 0.54 (DCM/Ace = 9:1)) and (2*RS*,4*SR*)*-***43b** (40 mg, 12%, *R*_*f*_ = 0.33 (DCM/Ace = 9:1)) as a yellow oil. ds: (2*RS*,4*RS*)*-***43b/**(2*RS*,4*SR*)*-***43b** = 75:25. Formula C_32_H_35_FN_2_O_2_, FW 498.64. Compound (2*RS*,4*RS*)*-***43b**: ^1^H NMR (300 MHz, chloroform-*d*) δ ppm 6.90–7.38 (m, 12H), 4.25–4.72 (m, 4H), 3.87–4.09 (m, 1H), 3.70–3.84 (m, 1H), 3.25–3.54 (m, 2H), 2.74–2.89 (m, 1H), 2.65 (d, *J* = 17.58 Hz, 1H), 2.29–2.50 (m, 3H), 1.42–2.20 (m, 4H), 1.02–1.33 (m, 5H). ^13^C NMR (75 MHz, chloroform-*d*) δ ppm 169.2, 163.8, 140.1, 137.9, 133.4, 129.4, 129.3, 129.3, 129.2, 128.5, 127.1, 125.5, 115.8, 115.7, 115.5, 115.4, 65.8, 65.6, 48.1, 47.3, 46.4, 44.8, 44.5, 43.3, 43.2, 42.8, 42.6, 32.4, 31.6, 25.3, 24.3, 23.9, 23.6, 23.4, 22.5, 11.8. HRMS-ESI^+^*m*/*z* [M + H]^+^ calcd for C_32_H_36_N_2_O_2_F, 499.2773; found, 499.2761. Compound (2*RS*,4*SR*)*-***43b**: ^1^H NMR (300 MHz, chloroform-*d*) δ ppm 6.90–7.38 (m, 12H), 4.25–4.72 (m, 4H), 3.87–4.09 (m, 1H), 3.70–3.84 (m, 1H), 3.25–3.54 (m, 2H), 2.74–2.89 (m, 1H), 2.65 (d, *J* = 17.58 Hz, 1H), 2.29–2.50 (m, 3H), 1.42–2.20 (m, 4H), 1.02–1.33 (m, 5H). ^13^C NMR (75 MHz, chloroform-*d*) δ ppm 169.3, 163.9, 140.1, 137.8, 133.4, 129.4, 129.3, 129.3, 129.2, 128.5, 127.1, 125.4, 115.8, 115.7, 115.5, 115.4, 65.8, 65.6, 48.2, 47.3, 46.4, 44.8, 44.5, 43.4, 43.2, 42.8, 42.5, 32.4, 31.6, 25.3, 24.3, 23.9, 23.5, 23.4, 22.5, 11.7.

#### 4.1.1.3 General Procedure for the Synthesis of the 4-Hydroxypentanamide and 4-Hydroxybutanamide Derivatives **45** and **47a**–**c** (GP3)

These reactions were performed under an argon atmosphere. The relevant 3-substituted 5-methyldihydrofuran-2(3*H*)-one or dihydrofuran-2(3*H*)-one derivative (1 equiv) was heated with the corresponding *N-*benzylamine (2 equiv) in dry THF at reflux for 48 h. When the reaction was complete, the mixture was cooled on ice, quenched with a 1 M aqueous solution of HCl (1.5 mL), and extracted with dichloromethane (3 × 10 mL). The combined organic fractions were dried over Na_2_SO_4_ and evaporated under vacuum. The crude product was purified by column chromatography.

##### 4.1.1.3.1 *N-*Benzyl-2-[(2-{[(diphenylmethylidene)amino]oxy}ethyl)(methyl)amino]-4-hydroxypentanamide ((2*RS*,4*RS*)*-***45** and (2*RS*,4*SR*)*-***45**)

Compounds (2*RS*,4*RS*)*-***45** and (2*RS*,4*SR*)*-***45** were prepared according to GP3 with 3-[(2-{[(diphenylmethylidene)amino]oxy}ethyl)(methyl)amino]-5-methyloxolan-2-one (**44**) (1.33 mmol, 0.47 g) and *N-*benzylamine (1.59 mmol, 0.17 g) in 10 mL of dry THF. The obtained crude products were purified by column chromatography over silica gel (DCM/Ace = 9:1) to yield (2*RS*,4*RS*)*-***45** (309 mg, 48%, *R*_*f*_ = 0.48 (S_1_)) and (2*RS*,4*SR*)*-***45** (103 mg, 16%, *R*_*f*_ = 0.25 (S_1_)) as a yellow oil. ds: (2*RS*,4*RS*)*-***45/**(2*RS*,4*SR*)*-***45** = 75:25. Formula C_28_H_33_N_3_O_3_, FW 459.58. Compound (2*RS*,4*RS*)*-***45**: ^1^H NMR (300 MHz, chloroform-*d*) δ ppm 1.20–1.26 (m, 4H (CH*CH*_3_)) 1.71–1.84 (m, 2H (CH*CH*_2_CH)) 2.24–2.30 (m, 3H (N*CH*_3_)) 2.77 (t, *J* = 5.26 Hz, 2H (OCH_2_*CH*_2_N)) 3.33–3.42 (m, 1H (N*CH*CO)) 3.66–3.82 (m, 1H (OH*CH*)) 4.09–4.36 (m, 4H (NH*CH*_2_, O*CH*_2_CH_2_N)) 7.07–7.12 (m, 2H (*Ar*)) 7.20–7.24 (m, 3H (*Ar*)) 7.27–7.47 (m, 10H (*Ar*)) 7.84–7.92 (m, 1H (NH*CH*_2_)). HRMS-ESI^+^*m*/*z* [M + H]^+^ calcd for C_28_H_34_N_3_O_3_, 460.2640; found, 460.2621. Compound (2*RS*,4*SR*)*-***45**^1^H NMR (300 MHz, chloroform-*d*) δ ppm 1.22–1.31 (m, 3H (CH*CH*_3_)) 1.62–1.89 (m, 3H (CH*CH*_2_CH)) 1.97–2.05 (m, 2H (N*CH*_3_)) 2.26–2.31 (m, 1H (N*CH*_3_)) 2.70–2.89 (m, 2H (OCH_2_*CH*_2_N)) 3.45 (dd, *J* = 8.72, 4.36 Hz, 1H (N*CH*CO)) 3.96–4.17 (m, 2H (NH*CH*_2_)) 4.20–4.34 (m, 3H (O*CH*_2_CH_2_N)) 4.38–4.46 (m, 1H (OH*CH*)) 7.08–7.14 (m, 2H (*Ar*)) 7.20–7.47 (m, 13H (*Ar*)). ^13^C NMR (126 MHz, chloroform-*d*) δ ppm 170.0, 157.6, 138.4, 136.1, 133.3, 129.7, 129.1, 129.0, 128.8, 128.7, 128.4, 128.3, 128.0, 127.9, 127.6, 127.6, 127.3, 65.1, 63.9, 53.4, 43.9, 38.6, 31.5, 29.8, 23.4. HRMS-ESI^+^*m*/*z* [M + H]^+^ calcd for C_28_H_34_N_3_O_3_, 460.2595; found, 460.2591.

##### 4.1.1.3.2 *N-*Benzyl-2-[(2-{[(diphenylmethylidene)amino]oxy}ethyl)(methyl)amino]-4-hydroxybutanamide (**47a**)

Compound **47a** was prepared according to GP3 with 3-[(2-{[(diphenylmethylidene)amino]oxy}ethyl)(methyl)amino]oxolan-2-one (**46**) (1.33 mmol, 0.45 g) and 1-phenylmethanamine (1.59 mmol, 0.17 g) in 10 mL of dry THF. The obtained crude product was purified by column chromatography over silica gel (DCM/Ace = 7:3) to yield **47a** (332 mg, 56%, *R*_*f*_ = 0.50 (DCM/Ace = 7:3)). Formula C_27_H_31_N_3_O_3_, FW 445.55. ^1^H NMR (300 MHz, chloroform-*d*) δ ppm 1.83–1.94 (m, 2H *(CH*_2_CH_2_OH*)*) 2.28 (s, 3H *(*Me*)*) 2.79 (t, *J* = 5.39 Hz, 2H *(CH*_2_N*)*) 3.28–3.35 (m, 1H *(*N*CH)*) 3.52–3.62 (m, 1H *(CH*_2_OH*)*) 3.78–3.87 (m, 1H *(CH*_2_OH*)*) 4.19 (dd, *J* = 14.88, 6.41 Hz, 2H *(CH*_2_O*)*) 4.23–4.31 (m, 2H *(*NH*CH*_2_*)*) 7.07–7.13 (m, 2H (Ar*)*) 7.18–7.47 (m, 13H (Ar*)*) 7.78 (t, *J* = 5.90 Hz, 1H (CO*NH)*). ^13^C NMR (300 MHz, chloroform-*d*) δ ppm 14.20, 27.29, 38.49, 43.02, 53.37, 60.40, 61.68, 67.48, 72.43, 127.21, 127.33, 127.83, 128.13, 128.30, 128.54, 128.93, 129.06, 129.49, 133.13, 136.14, 138.26, 157.15, 174.07. HRMS-ESI^+^*m*/*z* [M + H]^+^ calcd for C_27_H_32_N_3_O_3_, 446.2444; found, 446.2447.

##### 4.1.1.3.3 *N-*[(2-Chlorophenyl)methyl]-2-[(2-{[(diphenylmethylidene)amino]oxy}ethyl)(methyl)amino]-4-hydroxybutanamide (**47b**)

Compound **47b** was prepared according to GP3 with 3-[(2-{[(diphenylmethylidene)amino]oxy}ethyl)(methyl)amino]oxolan-2-one (**46**) (1.33 mmol, 0.45 g) and 1-(2-chlorophenyl)methanamine (1.59 mmol, 0.23 g) in 10 mL of dry THF. The obtained crude product was purified by column chromatography over silica gel (DCM/Ace = 7:3) to yield **47b** (223 mg, 35%, *R*_*f*_ = 0.52 (DCM/Ace = 7:3)). Formula C_27_H_30_ClN_3_O_3_, FW 480.01. ^1^H NMR (300 MHz, chloroform-*d*) δ ppm 1.82–1.92 (m, 2H *(CH*_2_CH_2_OH*)*) 2.28 (s, 3H *(*Me*)*) 2.80 (t, *J* = 5.51 Hz, 2H *(CH*_2_N*)*) 3.28–3.36 (m, 1H *(*N*CH)*) 3.50–3.60 (m, 1H *(CH*_2_OH*)*) 3.81 (dt, *J* = 11.09, 4.58 Hz, 1H *(CH*_2_OH*)*) 4.18–4.34 (m, 4H *(*NH*CH*_2_*; CH*_2_O*)*) 7.10–7.49 (m, 14H (Ar*)*) 7.86 (t, *J* = 5.90 Hz, 1H (CO*NH)*). ^13^C NMR (300 MHz, chloroform-*d*) δ ppm 27.22, 29.69, 38.54, 41.17, 53.38, 61.68, 67.31, 72.57, 126.91, 127.81, 128.08, 128.25, 128.57, 128.91, 129.02, 129.05, 129.39, 129.47, 133.09, 133.21, 135.54, 136.12, 157.18, 174.31. HRMS-ESI^+^*m*/*z* [M + H]^+^ calcd for C_27_H_31_N_3_O_3_Cl, 480.2054; found, 480.2056.

##### 4.1.1.3.4 *N-*[(3,4-Dichlorophenyl)methyl]-2-[(2-{[(diphenylmethylidene)amino]oxy}ethyl)(methyl)amino]-4-hydroxybutanamide (**47c**)

Compound **47c** was prepared according to GP3 with 3-[(2-{[(diphenylmethylidene)amino]oxy}ethyl)(methyl)amino]oxolan-2-one (**46**) (1.33 mmol, 0.45 g) and 1-(3,4-dichlorophenyl)methanamine (1.59 mmol, 0.30 g) in 10 mL of dry THF. The obtained crude product was purified by column chromatography over silica gel (DCM/Ace = 7:3) to yield **47c** (369 mg, 54%, *R*_*f*_ = 0.49 (DCM/Ace = 7:3)). Formula C_27_H_29_Cl_2_N_3_O_3_, FW 514.44. ^1^H NMR (300 MHz, chloroform-*d*) δ ppm 1.84–1.92 (m, 2H *(CH*_2_CH_2_OH*)*) 2.32 (s, 3H *(*Me*)*) 2.71–2.81 (m, 2H *(CH*_2_N*)*) 3.29–3.35 (m, 1H *(*N*CH)*) 3.54–3.64 (m, 1H *(CH*_2_OH*)*) 3.82 (dt, *J* = 11.09, 4.58 Hz, 1H *(CH*_2_OH*)*) 3.95 (d, *J* = 6.16 Hz, 1H *(CH*_2_O*)*) 4.01 (d, *J* = 6.67 Hz, 1H *(CH*_2_O*)*) 4.26–4.32 (m, 2H *(*NH*CH*_2_*)*) 6.89 (dd, *J* = 8.21, 2.05 Hz, 1H (Ar*)*) 7.08 (d, *J* = 2.05 Hz, 1H (Ar*)*) 7.23–7.46 (m, 11H (Ar*)*) 7.86 (t, *J* = 6.28 Hz, 1H (CO*NH)*). ^13^C NMR (300 MHz, chloroform-*d*) δ ppm 14.20, 27.23, 38.42, 41.81, 53.33, 61.62, 67.25, 71.99, 126.63, 127.86, 128.17, 128.36, 129.05, 129.15, 129.21, 129.59, 130.36, 131.03, 132.36, 133.08, 136.09, 138.73, 157.13, 174.45. HRMS-ESI^+^*m*/*z* [M + H]^+^ calcd for C_27_H_30_N_3_O_3_Cl_2_, 514.1664; found, 514.1666.

#### 4.1.1.4 General Procedure for the Synthesis of *N*-Benzyl-4-(1,3-dioxoisoindolin-2-yl)-2-(4-(diphenylmethylene)piperidin-1-yl)butanamide derivatives (**49a**,**b**) (GP4)

Anhydrous K_2_CO_3_ (4.40 mmol, 0.61 g, 2.5 equiv) and KI (1.7 mmol, 0.29 g) were added to a solution of amine **F** (diphenylmethanone *O*-(2-(methylamino)ethyl) oxime (**32**)) (1.76 mmol, 0.44 g, 1 equiv) in acetonitrile (10 mL). Then, the relevant *N-*benzyl-2-bromo-4-(1,3-dioxoisoindolin-2-yl)butanamide derivative (**48a**,**b**) (1.76 mmol, 1 equiv) was added, and the reaction mixture was stirred at reflux for 24 h. Then, the precipitate was filtered, the filtrate was concentrated under vacuum, and the product was purified by column chromatography.

##### 4.1.1.4.1 *N*-[(2-Chlorophenyl)methyl]-4-(1,3-dioxo-2,3-dihydro-1*H-*isoindol-2-yl)-2-[(2-{[(diphenylmethylidene)amino]oxy}ethyl)(methyl)amino]butanamide (**49a**)

According to GP4, amide **48a** (1.35 mmol, 0.59 g), amine **F** (diphenylmethanone *O*-(2-(methylamino)ethyl) oxime (**32**)) (1.35 mmol, 0.34 g), KI (1.22 mmol, 0.20 g), and anhydrous K_2_CO_3_ (3.11 mmol, 0.43 g) were combined in acetonitrile (15 mL). The obtained crude product was purified by column chromatography over silica gel (PE/EtOAc = 1:1) to yield **49a** (346 mg, 42%, *R*_*f*_ = 0.49 (PE/EtOAc (1:1)) as a yellow oil. Formula C_35_H_33_ClN_4_O_4_, FW 609.11. ^1^H NMR (chloroform-*d*) δ ppm 2.19 (dtd, *J* = 13.75, 8.06, 8.06, 6.03 Hz, 2H *(CH*_2_CH_2_NH*)*) 2.29 (s, 3H *(Me)*) 2.83 (t, *J* = 5.51 Hz, 2H *(CH*_2_N*)*) 3.26 (dd, *J* = 7.95, 4.87 Hz, 1H *(*N*CH)*) 3.76 (ddd, *J* = 14.04, 8.01, 6.41 Hz, 1H *(*O*CH*_2_*)*) 3.92 (ddd, *J* = 13.85, 8.21, 5.90 Hz, 1H *(*O*CH*_2_*)*) 4.18–4.33 (m, 4H *(*NH*CH*_2_*; CH*_2_phthalimide*)*) 7.13–7.35 (m, 12H (Ar*)*) 7.37–7.47 (m, 3H (Ar; CO*NH)*) 7.65–7.72 (m, 2H *(phthalimide)*) 7.79–7.85 (m, 2H *(phthalimide)*).

##### 4.1.1.4.2 *N-*[(3,4-Dichlorophenyl)methyl]-4-(1,3-dioxo-2,3-dihydro-1*H-*isoindol-2-yl)-2-[(2-{[(diphenylmethylidene)amino]oxy}ethyl)(methyl)amino]butanamide (**49b**)

According to GP4, amide **48b** (1.35 mmol, 0.63 g), amine **F** (diphenylmethanone *O*-(2-(methylamino)ethyl) oxime (**32**)) (1.35 mmol, 0.34 g), KI (1.22 mmol, 0.20 g), and anhydrous K_2_CO_3_ (3.11 mmol, 0.43 g) were combined in acetonitrile (15 mL). The obtained crude product was purified by column chromatography over silica gel (PE/EtOAc = 1:1) to yield **49b** (317 mg, 37%, *R*_*f*_ = 0.47 (PE/EtOAc (1:1)) as a yellow oil. Formula C_35_H_32_Cl_2_N_4_O_4_, FW 643.57. ^1^H NMR (300 MHz, chloroform-*d*) δ ppm 1.84–1.97 (m, 1H *(CH*_2_CH_2_NH*)*) 2.14–2.23 (m, 1H *(CH*_2_CH_2_NH*)*) 2.32 (s, 3H *(Me)*) 2.80 (t, *J* = 5.26 Hz, 2H *(CH*_2_N*)*) 3.77 (ddd, *J* = 13.91, 7.76, 6.54 Hz, 1H *(*N*CH)*) 3.85–3.99 (m, 2H *(*O*CH*_2_*)*) 3.99–4.09 (m, 2H *(CH*_2_phthalimide*)*) 4.25 (t, *J* = 5.26 Hz, 2H *(*NH*CH*_2_*)*) 6.91 (dd, *J* = 8.21, 2.05 Hz, 1H (Ar) 7.10 (d, *J* = 2.05 Hz, 1H (Ar*)*) 7.20–7.44 (m, 11H (Ar*)*) 7.44–7.50 (m, 1H (CO*NH)*) 7.66–7.74 (m, 2H *(phthalimide)*) 7.78–7.86 (m, 2H *(phthalimide)*).

#### 4.1.1.5 General Procedure for the Synthesis of 2-Substituted 4-Aminobutanamide Derivatives (**50a**,**b**) (GP5)

Hydrazine hydrate (2 equiv) was added to a suspension of a 2-substituted 4-phthalimidobutanoic acid derivative (1 equiv) in ethanol (10 mL). The solution was stirred at 60 °C for 2 h and at rt for 5 h. Then, the precipitate was filtered and washed with DCM (5 mL). The filtrate was evaporated, and the product was extracted two times with 8 mL of DCM. The combined organic fractions were dried over Na_2_SO_4_, and the obtained product was purified by column chromatography.

##### 4.1.1.5.1 4-Amino-*N-*[(2-chlorophenyl)methyl]-2-[(2-{[(diphenylmethylidene)amino]oxy}ethyl)(methyl)amino]butanamide (**50a**)

According to GP5, *N*-[(2-chlorophenyl)methyl]-4-(1,3-dioxo-2,3-dihydro-1*H-*isoindol-2-yl)-2-[(2-{[(diphenylmethylidene)amino]oxy}ethyl)(methyl)amino]butanamide (**49a**) (1 mmol, 0.61 g) and hydrazine hydrate (2 mmol, 0.11 g) were combined in ethanol (7 mL). The obtained crude product was purified by column chromatography over silica gel (EtOAc/MeOH = 8:2 → 25% NH_3_/MeOH/DCM/PE = 9:45:120:18) to yield **50a** (325 mg, 68%, *R*_*f*_ = 0.15 (PE/EtOAc (1:1)) as a yellow oil. Formula C_27_H_31_ClN_4_O_2_, FW 479.01. ^1^H NMR (chloroform-*d*) δ ppm 1.67–1.80 (m, 2H *(CH*_2_CH_2_NH_2_*)*) 2.28 (s, 3H *(*Me*)*) 2.67–2.78 (m, 1H *(CH*_2_N*)*) 2.78–2.88 (m, 3H *(CH*_2_NH_2_; *(CH*_2_N*)*) 3.22 (dd, *J* = 7.82, 5.26 Hz, 1H *(*N*CH)*) 4.12–4.35 (m, 4H *(*NH*CH*_2_*; CH*_2_O*)*) 7.09–7.45 (m, 14H (Ar*)*) 7.58 (t, *J* = 6.16 Hz, 1H (CO*NH)*). ^13^C NMR (300 MHz, chloroform-*d*) δ ppm 29.93, 38.57, 40.44, 40.95, 53.82, 65.23, 72.82, 126.86, 127.81, 127.81, 128.05, 128.05, 128.23, 128.23, 128.40, 128.84, 129.07, 129.07, 129.13, 129.31, 129.39, 133.17, 133.20, 136.02, 136.20, 156.96, 173.47.

##### 4.1.1.5.2 4-Amino-*N-*[(3,4-dichlorophenyl)methyl]-2-[(2-{[(diphenylmethylidene)amino]oxy}ethyl)(methyl)amino]butanamide (**50b**)

According to GP5, *N*-(3,4-dichlorobenzyl)-4-(1,3-dioxoisoindolin-2-yl)-2-((2-(((diphenylmethylene)amino)oxy)ethyl)(methyl)amino)butanamide (**49b**) (1 mmol, 0.64 g) and hydrazine hydrate (2 mmol, 0.11 g) were combined in ethanol (7 mL). The obtained crude product was purified by column chromatography over silica gel (EtOAc/MeOH = 8:2 → 25% NH_3_/MeOH/DCM/PE = 9:45:120:18) to yield **50b** (442 mg, 86%, *R*_*f*_ = 0.14 (PE/EtOAc (1:1)) as a yellow oil. Formula C_27_H_30_Cl_2_N_4_O_2_, FW 513.46. ^1^H NMR (chloroform-*d*) δ ppm 1.73–1.97 (m, 2H *(CH*_2_CH_2_NH_2_*)*) 2.31 (s, 3H *(*Me*)*) 2.73–2.81 (m, 2H *(CH*_2_N*)*) 2.82–2.93 (m, 2H *(CH*_2_NH_2_)) 3.26 (dd, *J* = 8.21, 4.87 Hz, 1H *(*N*CH)*) 3.90 (dd, *J* = 15.39, 6.16 Hz, 1H *(CH*_2_O*)*) 4.02 (dd, *J* = 15.26, 6.54 Hz, 1H *(CH*_2_O*)*) 4.27 (t, *J* = 5.13 Hz, 2H *(*NH*CH*_2_*)*) 6.89 (dd, *J* = 8.34, 2.18 Hz, 1H (Ar*)*) 7.09 (d, *J* = 1.80 Hz, 1H (Ar*)*) 7.22–7.45 (m, 11H (Ar*)*) 7.73 (t, *J* = 6.41 Hz, 1H (CO*NH)*). ^13^C NMR (300 MHz, chloroform-*d*) δ ppm 27.70, 38.41, 39.88, 41.73, 53.59, 65.69, 72.16, 126.64, 127.84, 127.84, 128.17, 128.17, 128.33, 128.33, 129.03, 129.15, 129.15, 129.54, 130.34, 130.92, 132.30, 133.13, 136.09, 138.99, 138.99, 157.04, 173.61. HRMS-ESI^+^*m*/*z* [M + H]^+^ calcd for C_29_H_31_N_4_O_2_Cl_2_, 513.1824; found, 513.1818.

#### 4.1.1.6 General Procedure for the Synthesis of 2-Substituted 4-Acetamidobutanamide Derivatives (**51a**,**b**) (GP6)

A mixture of acetic acid (2 equiv) and *N*,*N*′-dicyclohexylcarbodiimide (DCC) (2 equiv) in 5 mL of DCM was stirred at 0 °C for 10 min. Then, a 4-aminobutanamide derivative (1 equiv) and 4-dimethylaminopyridine (DMAP) (2 equiv) were added to the reaction mixture and stirring continued for 20 h at room temperature. The obtained *N*,*N-*dicyclohexylurea (DCU) was filtered, the filtrate was evaporated, and the product was purified by column chromatography over silica gel (S_11_ (Chl/Ace 1:1)).

##### 4.1.1.6.1 *N-*[(2-Chlorophenyl)methyl]-2-[(2-{[(diphenylmethylidene)amino]oxy}ethyl)(methyl)amino]-4-acetamidobutanamide (**51a**)

According to GP6, acetic acid (0.63 mmol, 0.037 g, 36 μL), DCC (0.63 mmol, 0.13 g), 4-aminobutanamide (**50a**) (0.15 g, 0.32 mmol), and DMAP (0.63 mmol, 0.08 g) were combined in 5 mL of DCM. Compound **51a** was obtained as a yellow oil (140 mg, 86%). *R*_*f*_ = 0.45 (Chl/Ace = 1:1). Formula C_29_H_33_Cl_1_N_4_O_3_, FW 521.05. ^1^H NMR (chloroform-*d*) δ ppm 1.78–1.93 (m, 5H *(CH*_2_CH_2_NH; Me*)*) 2.27 (s, 3H *(Me)*) 2.79 (t, *J* = 5.39 Hz, 2H *(CH*_2_N*)*) 3.11–3.23 (m, 2H *(CH*_2_NH*)*) 3.33–3.45 (m, 1H *(*N*CH)*) 4.16–4.34 (m, 4H *(*NH*CH*_2_*; CH*_2_O*)*) 6.94 (br s, 1H (CO*NH)*) 7.12–7.44 (m, 14H (Ar*)*) 7.75 (t, *J* = 6.03 Hz, 1H (CO*NH)*). ^13^C NMR (300 MHz, chloroform-*d*) δ ppm 23.25, 24.34, 38.53, 39.00, 41.10, 53.42, 66.52, 66.52, 72.54, 126.87, 127.80, 127.80, 128.06, 128.06, 128.24, 128.24, 128.54, 128.87, 128.88, 129.03, 129.03, 129.43, 129.45, 133.14, 133.21, 135.68, 136.11, 157.11, 170.30, 173.88. HRMS-ESI^+^*m*/*z* [M + H]^+^ calcd for C_29_H_34_N_4_O_3_Cl, 5521.2298; found, 521.22313.

##### 4.1.1.6.2 *N-*[(3,4-Dichlorophenyl)methyl]-2-[(2-{[(diphenylmethylidene)amino]oxy}ethyl)(methyl)amino]-4-acetamidobutanamide (**51b**)

According to GP6, acetic acid (0.63 mmol, 0.037 g, 36 μL), DCC (0.63 mmol, 0.13 g), 4-aminobutanamide (**50b**) (0.16 g, 0.32 mmol), and DMAP (0.63 mmol, 0.08 g) were combined in 5 mL of DCM. Compound **51b** was obtained as a yellow oil (173 mg, 86%). *R*_*f*_ = 0.47 (Chl/Ace = 1:1). Formula C_29_H_32_Cl_2_N_4_O_3_, FW 555.50 ^1^H NMR (chloroform-*d*) δ ppm 1.77–1.89 (m, 5H *(CH*_2_CH_2_NH; Me*)*) 2.26 (s, 3H *(Me)*) 2.69 (t, *J* = 5.38 Hz, 2H *(CH*_2_N*)*) 3.09–3.26 (m, 2H *(CH*_2_NH*)*) 3.31–3.44 (m, 1H *(*N*CH)*) 4.12–4.31 (m, 4H *(*NH*CH*_2_*; CH*_2_O*)*) 6.67 (br s, 1H (CO*NH)*) 7.09–7.45 (m, 13H (Ar*)*) 7.73 (t, *J* = 6.07 Hz, 1H (CO*NH)*). ^13^C NMR (300 MHz, chloroform-*d*) δ ppm 23.15, 24.44, 38.43, 39.10, 41.15, 53.42, 66.62, 66.68, 72.44, 126.77, 127.86, 127.99, 128.12, 128.12, 128.34, 128.34, 128.44, 128.89, 128.89, 129.03, 129.03, 129.53, 129.59, 133.14, 133.28, 135.78, 136.21, 157.31, 170.36, 173.90. HRMS-ESI^+^*m*/*z* [M + H]^+^ calcd for C_29_H_33_N_4_O_3_Cl_2_, 555.1930; found, 555.1919.

#### 4.1.1.7 General Procedures for the Synthesis of 2-Substituted 3-Hydroxypropanamide Derivatives **54a**–**c**, **55a**–**f**, **56a**–**f**, **57**, **58**, **59a**–**c** (GP7)

The corresponding amine **A**–**F** (1.2 equiv), a relevant amide **53a**–**e** (1 equiv), and TBAB (0.1 equiv) were dissolved in dry DMF (for **55a**–**e**, **56a**–**e** dry DCM was used), and DIPEA (1 equiv) was then added. After stirring at reflux for 16 h, extraction with DCM and deionized water was performed. The organic phase was washed with saturated NaHCO_3_ and dried with anhydrous Na_2_SO_4_. The solvent was evaporated, and the product was purified by column chromatography over silica gel (S_12_ DCM/Ace = 7:3).

##### 4.1.1.7.1 *N-*Benzyl-2-{4-[bis(3-methylthiophen-2-yl)methylidene]piperidin-1-yl}-3-hydroxypropanamide (**54a**)

The synthesis was done according to GP7 with amine **A** (**17**) (1.39 mmol, 400 mg), amide **53a** (1.16 mmol, 300 mg), TBAB (0.12 mmol, 40 mg), DIPEA (1.16 mmol, 201 μL), and DMF (3 mL). The obtained crude product was purified by column chromatography over silica gel (DCM/Ace = 7:3) to yield **54a** (260 mg, 48%, *R*_*f*_ = 0.33 and 0.51 (S_6_) as a yellow oil. Formula C_26_H_30_N_2_O_2_S_2_, FW: 466.66. ^1^H NMR (300 MHz, chloroform-*d*) δ ppm 2.00–2.12 (m, 6 H) 2.25–2.47 (m, 3 H) 2.67–2.82 (m, 2 H) 2.85–2.98 (m, 2 H) 3.44–3.55 (m, 1 H) 3.86–4.06 (m, 2 H) 4.47 (dd, *J* = 16.70, 6.15 Hz, 2 H) 5.30 (s, 1 H) 6.77 (d, *J* = 5.28 Hz, 2 H) 7.14 (d, *J* = 5.27 Hz, 1 H) 7.21–7.39 (m, 5 H) 7.97–8.09 (m, 1 H). ^13^C NMR (75 MHz, chloroform-*d*) δ ppm 171.3, 141.6, 138.0, 137.4, 133.9, 129.7, 129.5, 128.8, 128.7, 127.7, 127.5, 127.5, 124.6, 124.1, 121.0, 68.1, 58.7, 53.6, 51.3, 43.2, 31.4, 14.7. HRMS-ESI^+^*m*/*z* [M + H]^+^ calcd for C_26_H_30_N_2_O_2_S_2_, 467.1827; found, 467.1843.

##### 4.1.1.7.2 2-{4-[Bis(3-methylthiophen-2-yl)methylidene]piperidin-1-yl}-*N-*[(2-chlorophenyl)methyl]-3-hydroxypropanamide (**54b**)

The synthesis was done according to GP7 with amine **A** (**17**) (1.64 mmol, 470 mg), amide **53b** (1.37 mmol, 400 mg), TBAB (0.14 mmol, 44 mg), DIPEA (1.37 mmol, 237 μL), and DMF (5 mL). The obtained crude product was purified by column chromatography over silica gel (S_12_) to yield **54b** (315 mg, 46%, *R*_*f*_ = 0.16 and 0.35 (S_9_)) as a yellow oil. Formula C_26_H_29_ClN_2_O_2_S_2_, FW: 501.10. ^1^H NMR (300 MHz, chloroform-*d*) δ ppm 8.58–8.69 (m, 1H), 7.28–7.38 (m, 2H), 7.10–7.23 (m, 4H), 6.77 (d, *J* = 5.27 Hz, 2H), 4.51 (t, *J* = 5.27 Hz, 2H), 3.93–4.17 (m, 3H), 3.19 (s, 2H), 2.98–3.14 (m, 2H), 2.54 (d, *J* = 4.10 Hz, 4H), 2.07 (s, 6H). ^13^C NMR (75 MHz, chloroform-*d*) δ ppm 168.4, 139.1, 136.8, 134.9, 134.2, 133.4, 129.7, 129.6, 129.0, 127.1, 124.4, 122.3, 67.5, 58.7, 51.1, 41.3, 30.0, 14.6. HRMS-ESI^+^*m*/*z* [M + H]^+^ calcd for C_26_H_29_ClN_2_O_2_S_2_, 501.1441; found, 501.1037.

##### 4.1.1.7.3 2-{4-[Bis(3-methylthiophen-2-yl)methylidene]piperidin-1-yl}-*N-*[(4-fluorophenyl)methyl]-3-hydroxypropanamide (**54c**)

The synthesis was done according to GP7 with amine **A** (**17**) (0.70 mmol, 200 mg), amide **53c** (0.58 mmol, 160 mg), TBAB (0.06 mmol, 19 mg), DIPEA (0.58 mmol, 100 μL), and DMF (3 mL). The obtained crude product was purified by column chromatography over silica gel (DCM/Ace = 7:3) to yield **54c** (100 mg, 36%, *R*_*f*_ = 0.24 and 0.55 (S_12_)) as a yellow oil. Formula C_26_H_29_FN_2_O_2_S_2_, FW: 484.65. ^1^H NMR (300 MHz, chloroform-*d*) δ ppm 8.25–8.40 (m, 1H), 7.19–7.33 (m, 2H), 7.13 (d, *J* = 4.69 Hz, 2H), 6.98 (s, 2H), 6.77 (d, *J* = 5.28 Hz, 2H), 4.39 (dd, *J* = 5.86, 11.14 Hz, 2H), 3.86–4.11 (m, 2H), 3.67–3.81 (m, 1H), 3.00–3.14 (m, 2H), 2.83–2.96 (m, 2H), 2.45 (t, *J* = 5.27 Hz, 4H), 2.07 (s, 6H). ^13^C NMR (75 MHz, chloroform-*d*) δ ppm 169.9, 163.7, 160.5, 140.2, 137.1, 134.0, 133.7, 133.7, 129.5, 129.3, 129.2, 124.3, 121.8, 115.7, 115.5, 67.8, 58.6, 51.2, 42.5, 30.8, 14.6. HRMS-ESI^+^*m*/*z* [M + H]^+^ calcd for C_26_H_29_FN_2_O_2_S_2_, 485.1733; found, 485.1728.

##### 4.1.1.7.4 2-((3-(5*H-*Dibenzo[*a*,*d*][7]annulen-5-ylidene)propyl)(methyl)amino)-*N-*benzyl-3-hydroxypropanamide (**55a**)

The synthesis was done according to GP7 with amine **B** (**20**) **(**1.2 mmol, 313 mg), amide **53a** (1 mmol, 257 mg), TBAB (0.01 mmol, 0.32 mg), DIPEA (1 mmol, 174 μL), DCM 5 mL. The obtained crude product was purified by column chromatography over silica gel (DCM/Ace = 7:3) to yield **55a** (131 mg, 30%, *R*_*f*_ = 0.65 and 0.42 (S_12_)). ^1^H NMR (chloroform-*d*): δ ppm 7.61 (t. 1H, N*H*), 7.40–7.05 (m, 10H, Ar), 6.98–6.89 (m, 1H, ArC*H*=CHAr), 6.88–6.63 (m, 4H, ArCH=C*H*Ar, Ar), 5.42–5.22 (m, 1H, C*H*=), 4.38–4.16 (m, 2H, C*H*_2_NH), 3.89 (ddd, 1H, *J* = 11.1; 7.7; 4.7 Hz, C*H*_2_′OH), 3.74 (ddd, 1H, *J* = 11.1; 5.2; 4.2 Hz, C*H*NCH_3_), 3.12 (dt, 1H, *J* = 8.0; 4.5 Hz, C*H*_2_″OH), 2.71–2.38 (m, 2H, C*H*_2_CH_2_C=), 2.38–2.16 (m, 2H, CH_2_C*H*_2_C=), 1.97 (d, 3H, CH_3_–N). OH proton was not detected. ^13^C NMR (chloroform-*d*): δ ppm 174.02 (*C*ONH), 143.60 (Ph_2_*C*=), 142.30 (*C*_Dche_), 142.20 (*C*_Dche_), 137.01(*C*_Dche_), 136.96 (*C*_Dche_), 134.81 (*C*_Ph_), 133.70 (*C*_Dche_), 133.24 (*C*_Dche_), 131.21 (*C*_Dche_), 131.11 (*C*_Dche_), 130.96 (Ar*C*H=CHAr), 130.88 (ArCH=*C*HAr), 129.50 (*C*_Dche_), 129.42(*C*_Dche_), 129.48 (*C*H=CPh_2_), 128.96 (*C*_Dche_), 128.68 (*C*_Dche_), 128.26 (*C*_Ph_), 128.00 (*C*_Ph_), 127.25 (*C*_Ph_), 127.06(*C*_Ph_), 126.98(*C*_Ph_), 126.84 (*C*_Dche_), 67.55 (*C*HNCH_3_), 66.78 (C*H*_2_OH), 53.26 (*C*H_2_CH_2_CH=), 40.68 (*C*H_2_NH), 38.50 (*C*H_3_N), 27.15 (CH_2_*C*H_2_CH=) HRMS-ESI^+^*m*/*z* [M + H]^+^ calcd for C_29_H_30_N_2_O_2_, 439.2386; found, 439.2374.

##### 4.1.1.7.5 2-((3-(5*H-*Dibenzo[*a*,*d*][7]annulen-5-ylidene)propyl)(methyl)amino)-*N-*(2-chlorobenzyl)-3-hydroxypropanamide **(55b)**

The synthesis was done according to GP7 with amine **B** (**20**) **(**1.2 mmol, 313 mg), amide **53b** (1 mmol, 290 mg), TBAB (0.01 mmol, 0.32 mg), DIPEA (1 mmol, 174 μL), DCM 5 mL. The obtained crude product was purified by column chromatography over silica gel (DCM/Ace = 7:3) to yield **55a** (141 mg, 30%, *R*_*f*_ = 0.65 and 0.42 (S_12_)). ^1^H NMR (chloroform-*d*): δ ppm 7.66 (d, 1H, N*H*), 7.42–7.04 (m, 12H, Ar), 6.89–6.77 (m, 1H, ArC*H*=CHAr), 6.69 (dd, 1H, ArCH=C*H*Ar), 5.46 (m, 1H, C*H*=), 4.46–4.18 (m, 2H, C*H*_2_NH), 4.12 (q, 1H, *J* = 7.1 Hz, C*H*_2_′OH), 3.91 (ddd, 1H, *J* = 10.2; 8.0; 2.0 Hz, C*H*NCH_3_), 3.16 (tt, 1H, C*H*_2_″OH), 2.78–2.44 (m, 2H, C*H*_2_CH_2_C=), 2.39–2.16 (m, 2H, CH_2_C*H*_2_C=), 2.10–1.99 (m, 3H, CH_3_–N) ppm. OH proton was not detected. ^13^C NMR (chloroform-*d*): δ ppm 173.81 (*C*ONH), 143.67 (Ph_2_*C*=), 142.39 (*C*_Ph_), 135.40 (*C*_Ph_), 137.05 (*C*_Dche_), 137.00 (*C*_Dche_), 134.87 (*C*_Dche_), 134.74 (*C*_Dche_), 133.68 (*C*_Dche_), 131.16 (*C*_Dche_), 131.04 (ArCH=*C*HAr), 131.00 (Ar*C*H=CHAr), 128.82 (*C*_Ph_), 128.68 (*C*_Ph_), 128.44 (*C*_Ph_), 128.28 (*C*_Ph_), 128.03 (*C*_Dche_), 127.99 (*C*_Dche_), 127.40 (*C*_Dche_), 127.30 (*C*_Dche_), 127.02 (*C*=CPh_2_), 126.97 (*C*_Dche_), 126.89(*C*_Dche_), 67.81 (*C*HNCH_3_), 58.55(*C*H_2_OH), 57.67 (*C*H_2_CH_2_CH=), 42.73 (*C*H_2_NH), 38.80 (*C*H_3_N), 27.16 (CH_2_*C*H_2_CH=).

##### 4.1.1.7.6 2-((3-(5*H-*Dibenzo[*a*,*d*][7]annulen-5-ylidene)propyl)(methyl)amino)-*N-*(4-chlorobenzyl)-3-hydroxypropanamide (**55c**)

The synthesis was done according to GP7 with amine **B** (**20**) **(**1.2 mmol, 313 mg), amide **53c** (1 mmol, 290 mg), TBAB (0.01 mmol, 0.32 mg), DIPEA (1 mmol, 174 μL), DCM 5 mL. The obtained crude product was purified by column chromatography over silica gel (DCM/Ace = 7:3) to yield **55a** (141 mg, 30%, *R*_*f*_ = 0.65 and 0.41 (S_12_)). ^1^H NMR (chloroform-*d*): δ ppm 7.53 (dt, 1H, N*H*), 7.41–7.07 (m, 9H, Ar), 6.92–6.65 (m, 5H, ArC*H*=C*H*Ar, Ar), 5.49–5.29 (m, 1H, C*H*=), 4.44–4.22 (m, 2H, C*H*_2_NH), 4.06–3.91 (m, 1H, C*H*_2_′OH), 3.92–3.65 (m, 1H, C*H*NCH_3_), 3.10 (m, 1H, C*H*_2_″OH), 2.55–2.38 (m, 2H, C*H*_2_CH_2_C=), 2.31–2.13 (m, 2H, CH_2_C*H*_2_C=), 2.04 (s, 3H, C*H*_3_N) ppm. OH proton was not detected. ^13^C NMR (chloroform-*d*): δ ppm 173.92 (*C*ONH), 143.81 (Ph_2_*C*=), 143.24 (*C*_Dche_), 142.39 (*C*_Dche_), 137.00 (*C*_Dche_), 136.93 (*C*_Dche_), 134.75 (*C*_Ph_), 133.85 (*C*_Dche_), 133.64 (*C*_Dche_), 133.14 (*C*_Dche_), 133.11 (*C*_Dche_), 131.36 (*C*_Ph_-Cl), 131.29 (*C*_Ph_), 130.96 (ArCH=*C*HAr), 130.86 (Ar*C*H=CHAr), 129.00 (*C*H=CPh_2_), 128.68 (*C*_Ph_), 128.51(*C*_Ph_), 128.32 (*C*_Ph_), 128.09 (*C*_Ph_), 127.97 (*C*_Dche_), 127.45 (*C*_Dche_), 127.37 (*C*_Dche_), 126.94 (*C*_Dche_), 67.10 (*C*HNCH_3_), 59.96 (*C*H_2_OH), 57.35 (*C*H_2_CH_2_CH=), 42.07 (CH_2_NH), 41.08 (*C*H_3_N), 26.35 (CH_2_*C*H_2_CH=).

##### 4.1.1.7.7 2-((3-(5*H-*Dibenzo[*a*,*d*][7]annulen-5-ylidene)propyl)(methyl)amino)-*N-*(4-methylbenzyl)-3-hydroxypropanamide (**55d**)

The synthesis was done according to GP7 with amine **B** (**20**) **(**1.2 mmol, 313 mg), amide **53d** (1 mmol, 276 mg), TBAB (0.01 mmol, 0.32 mg), DIPEA (1 mmol, 174 μL), DCM 5 mL. The obtained crude product was purified by column chromatography over silica gel (DCM/Ace = 7:3) to yield **55a** (136 mg, 30%, *R*_*f*_ = 0.66 and 0.43 (S_12_)). ^1^H NMR (chloroform-*d*): δ ppm 7.62 (dt, 1H, N*H*), 7.44–7.12 (m, 8H, Ar), 7.08–6.63 (m, 6H, ArC*H*=C*H*Ar, Ar), 5.41–5.06 (m, 1H, C*H*=), 4.32–4.15 (m, 2H, C*H*_2_NH), 4.06–3.82 (m, 1H, C*H*_2_′OH), 3.79–3.66 (m, 1H, C*H*NCH_3_), 3.18–3.02 (m, 1H, C*H*_2_″OH), 2.54–2.05 (m, 4H, C*H*_2_C*H*_2_C=), 1.93 (s, 3H, NC*H*_3_). OH proton was not detected. ^13^C NMR (chloroform-*d*): δ ppm 173.84 (*C*ONH), 161.89 (d, ^1^*J*_*C–F*_ = 245.2 Hz, *C*_Ar_-F), 143.79 (Ph_2_*C*=), 137.01 (*C*_Dche_), 136.95 (*C*_Dche_), 134.87 (*C*_Dche_), 134.24 (*C*_Ar_-CH_2_), 134.17 (d, ^4^*J*_*C–F*_ = 4.0 Hz, *C*_Ar_), 134.14 (*C*_Dche_), 131.10 (Ar*C*H=CHAr), 130.91 (Ar*C*H=CHAr), 129.16 (*C*_Ar_), 129.05, 128.94 (*C*_Ar_), 128.64 (d, ^3^*J*_*C–F*_ = 7.4 Hz, *C*_Ar_), 128.59(*C*_Dche_), 128.08 (*C*_Dche_), 127,42 (*C*_Ar_), 126.94 (*C*_Ar_), 115.52 (*C*_Ar ortoF_), 115.20 (d, ^2^*J*_*C–F*_ = 21.5 Hz, *C*_Ar_), 67.96 (*C*HNCH_3_), 58.65 (*C*H_2_OH), 55.19 (*C*H_2_CH_2_CH=), 42.00 (*C*H_2_Ph), 36.92 (*C*H_3_N), 26.61 (CH_2_*C*H_2_CH=)

##### 4.1.1.7.8 2-((3-(5*H-*Dibenzo[*a*,*d*][7]annulen-5-ylidene)propyl)(methyl)amino)-*N-*(4-methylbenzyl)-3-hydroxypropanamide (**55e**)

The synthesis was done according to GP7 with amine **B** (**20**) **(**1.2 mmol, 313 mg), amide **53e** (1 mmol, 271 mg), TBAB (0.01 mmol, 0.32 mg), DIPEA (1 mmol, 174 μL), DCM 5 mL. The obtained crude product was purified by column chromatography over silica gel (DCM/Ace = 7:3) to yield **55a** (135 mg, 30%, *R*_*f*_ = 0.66 and 0.42 (S_12_)). ^1^H NMR (chloroform-*d*): δ ppm 7.57 (t, 1H, N*H*), 7,43–7.11 (m, 10H, Ar), 7.07–6.95 (m, 2H, Ar), 6.91–6.79 (m, 1H, ArC*H*=C*H*Ar), 6.77–6.67 (m, 1H, ArC*H*=C*H*Ar), 5.52–5.26 (m, 1H, C*H*=), 4.50–4.03 (m, 2H, C*H*_2_NH), 3.90 (ddd, 1H, *J* = 11.0; 8,0; 5.6 Hz, C*H*_2_′OH), 3.74 (dt, 1H, *J* = 11.1; 4.5 Hz, C*H*NCH_3_), 3.13 (td, 1H, *J* = 8.1; 3.8 Hz, C*H*_2_″OH), 2.74–2.37 (m, 2H, C*H*_2_CH_2_C=), 2.32 (d, 2H, CH_2_C*H*_2_C=), 2.10–1.95 (m, 6H, C*H*_3_-N, C*H*_3_Ar). OH proton was not detected. ^13^C NMR (chloroform-*d*): δ ppm 173,80 (*C*ONH), 143,66 (Ph_2_*C*=), 137.07 (*C*_Dche_), 137.03 (*C*_Dche_), 136.97 (*C*_Dche_), 136.78 (*C*_Dche_), 135.28 (*C*_Dche_), 135.26 (*C*_Ar_-CH_2_), 134.88 (*C*_Ar_-CH_3_), 134.75 (*C*_Dche_), 133,77 (*C*_Dche_), 133,72(*C*_Dche_), 131.19 (Ar*C*H=CHAr), 131.07 (Ar*C*H=CHAr), 129.29 (*C*_Dche_), 128,79 (*C*_Dche_), 128,66 (*C*_Dche_), 128,28 (*C*_Dche_), 128,02 (*C*_Ar_), 127.47(*C*_Ar_), 127.39(*C*_Ar_), 127.03(*C*_Ar_), 66.90 (*C*HNCH_3_), 60.37 (*C*H_2_OH), 58,39 (*C*H_2_CH_2_CH=), 42.40 (*C*H_2_Ar), 38,79 (*C*H_3_N), 26.85 (CH_2_CH_2_*C*H=), 21.09 (*C*H_3_Ar).

##### 4.1.1.7.9 *N-*Benzyl-2-((3-(10,11-dihydro-5*H-*dibenzo[*a*,*d*][7]annulen-5-ylidene)propyl)(methyl)amino)-3-hydroxypropanamide (**56a**)

The synthesis was done according to GP7 with amine **C** (**21**) (1.2 mmol, 315 mg), amide **53a** (1 mmol, 257 mg), TBAB (0.01 mmol, 0.32 mg), DIPEA (1 mmol, 174 μL), DCM 5 mL. The obtained crude product was purified by column chromatography over silica gel (DCM/Ace = 7:3) to yield **56a** (132 mg, 30%, *R*_*f*_ = 0.70 or 0.57 (S_12_)). ^1^H NMR (chloroform-*d*): δ ppm 7.57 (br s, 1H, N*H*CO), 6.98–7.37 (m, 13H, Ar), 5.76 (t, 1H, C*H*=), 4.37–4.48 (m, 3H, C*H*_2_′OH, C*H*_2_Ar), 4.06 (t, 1H, C*H-*NCH_3_), 3,13–3,44 (m, 3H, Ar*CH*_2_-CH_2_Ar, C*H*_2_′OH), 2.83–3,03 (m, 2H, ArCH_2_–C*H*_2_Ar), 2.68–2.81 (m, 2H, C*H*_2_CH_2_C=), 2.21–2.34 (m, 2H, CH_2_C*H*_2_C=), 2.17 (s, 3H, C*H*_3_). OH proton was not detected. ^13^C NMR (chloroform-*d*): δ ppm 172.97 (*C*ONH), 144.42 (*C*_Ar_), 140.95 (*C*H=), 139.75 (*C*_Ar_), 139.25 (*C*_Ar_), 138,09 (*C*_Ar_), 137.02 (*C*_Ar_), 130.04 (*C*_Ar_), 128,64 (*C*_Ar_), 128,47 (*C*_Ar_), 128,33 (*C*_Ar_), 128,11 (*C*_Ar_), 128,04 (*C*_Ar_), 127.57 (*C*_Ar_), 126.07 (*C*_Ar_), 125.76 (*C*_Ar_), 127.19 (*C*_Ar_) 126.07 (*C*_Ar_), 125.76 (*C*_Ar_), 66.92 (*C*HN), 59.98 (*C*H_2_OH), 57.34 (*C*H_2_CH_2_CH=), 42.87 (*C*H_2_NH), 41.25 (*C*H_3_N), 33.70 (ArCH_2_-*C*H_2_Ar), 32.01 (Ar*C*H_2_–CH_2_Ar), 27.19 (CH_2_*C*H_2_CH=). HRMS-ESI^+^*m*/*z* [M + H]^+^ calcd for C_29_H_33_N_2_O_2_, 441.2542; found, 441.2530.

##### 4.1.1.7.10 *N-*(2-Chlorobenzyl)-2-((3-(10.11-dihydro-5*H-*dibenzo[*a*,*d*][7]annulen-5-ylidene)propyl)(methyl)amino)-3-hydroxypropanamide (**56b**)

The synthesis was done according to GP7 with amine **C** (**21**) (1.2 mmol, 315 mg), amide **53b** (1 mmol, 290 mg), TBAB (0.01 mmol, 0.32 mg), DIPEA (1 mmol, 174 μL), DCM 5 mL. The obtained crude product was purified by column chromatography over silica gel (DCM/Ace = 7:3) to yield **56b** (128 mg, 30%, *R*_*f*_ = 0.74 or 0.55 (S_12_)). ^1^H NMR (chloroform-*d*): δ ppm 7.52–7.83 (m, 1H, CON*H*), 6.89–7.37 (m, 12H, Ar), 5.74 (br s, 1H, C*H*=C), 3,75–4.51 (m, 4H, C*H*_2_OH, C*H*_2_Ar), 2.84–3,06 (m, 3H, Ar*CH*_2_-CH_2_Ar, C*H*N), 2.66–2.81 (m, 2H, ArCH_2_C*H*_2_Ar) 2.46–264 (m, 2H, C*H*_2_CH_2_CH=), 2.21–2.37 (m, 2H, CH_2_C*H*_2_CH=), 2.08–2.19 (m, 3H, CH_3_) ppm. OH proton was not detected. ^13^C NMR (chloroform-*d*): δ ppm 173,20 (*C*ONH), 144.43 (*C*_Ar_), 140.92 (CH=*C*), 139.71 (*C*_Ar_), 139.27 (*C*_Ar_), 136.97(*C*_Ar_), 136.68 (*C*_Ar_), 130.06(*C*_Ar_), 129.48 (*C*_Ar_), 128,94 (*C*_Ar_), 128,72(*C*_Ar_), 128,41 (*C*_Ar_), 128,16(*C*_Ar_), 127.63(*C*_Ar_), 127.47 (*C*_Ar_), 126.14 (*C*_Ar_), 125.77 (*C*H=C), 66.94 (*C*HN), 59.99 (*C*H_2_OH), 57.35 (*C*H_2_CH_2_CH=), 42.13 (*C*H_2_NH), 41.21 (*C*H_3_N), 33,71 (ArCH_2_-*C*H_2_Ar), 32.01 (Ar*C*H_2_-CH_2_Ar), 27.18 (CH_2_*C*H_2_CH=) HRMS-ESI^+^*m*/*z* [M + H]^+^ calcd for C_29_H_32_N_2_O_2_Cl, 475.2152; found, 475.2142

##### 4.1.1.7.11 *N-*(4-Chlorobenzyl)-2-((3-(10,11-dihydro-5*H-*dibenzo[*a*,*d*][7]annulen-5-ylidene)propyl)(methyl)amino)-3-hydroxypropanamide (**56c**)

The synthesis was done according to GP7 with amine **C** (**21**) (1.2 mmol, 315 mg), amide **53c** (1 mmol, 290 mg), TBAB (0.01 mmol, 0.32 mg), DIPEA (1 mmol, 174 μL), DCM 5 mL. The obtained crude product was purified by column chromatography over silica gel (DCM/Ace = 7:3) to yield **55c** (132 mg, 30%, *R*_*f*_ = 0.74 or 0.57 (S_12_)). ^1^H NMR (chloroform-*d*): δ ppm 7.73–7.57 (m, 1H, CON*H*) 7.15–7.10 (m, 10H, Ar) 7.01–6.61 (m, 2H, Ar) 5.78 (d, 1H, *J* = 8,2 Hz, C*H*=), 4.49–4.33 (m, 2H, NHC*H*_2_Ar), 3.96 (dd, 1H, *J* = 11.2; 7.6 Hz, C*H*_2_′OH), 3.81 (dd, 1H, *J* = 11.2, 4.2 Hz, C*H*_2_″OH), 3,23 (dd, 1H, *J* = 7.6, 4.1 Hz, C*H*N), 2.96–2.74 (m, 4H, ArC*H*_2_C*H*_2_Ar), 2.62 (dt, 2H, *J* = 13.0, 6.5 Hz, C*H*_2_CH_2_CH=), 2.42–2.26 (m, 2H, CH_2_C*H*_2_CH=), 2.20 (s, 3H, C*H*_3_). Proton was not detected. ^13^C NMR (chloroform-*d*): δ ppm 173.75 (NH*C*O), 144.57(*C*_Ar_), 140.80 (CH=*C*),139.28 (Ar), 136.91(Ar), 135.40 (*C*_Ar_), 133,51 (*C*_Ar_), 128,88 (Ar), 128,47 (Ar), 128.56 (Ar), 128,41 (Ar), 128,12 (Ar), 127.60 (*C*_Ar_), 127.21(*C*_Ar_), 127.23 (*C*_Ar_), 126.09 (*C*_Ar_), 125.79 (*C*_Ar_), 86.21 (*C*H=C), 74.01 (*C*HN), 60.41 (*C*H_2_OH), 58,22 (*C*H_2_CH_2_CH=), 40.96 (NH*C*H_2_Ar), 38.00 (*C*H_3_N), 32.04 (Ar*C*H_2_-*C*H_2_Ar), 28.31 (CH_2_*C*H_2_CH=).

##### 4.1.1.7.12 *N-*(4-Fluorobenzyl)-2-((3-(10,11-dihydro-5*H-*dibenzo[*a*,*d*][7]annulen-5-ylidene)propyl)(methyl)amino)-3-hydroxypropanamide (**56d**)

The synthesis was done according to GP7 with amine **C** (**21**) (1.2 mmol, 315 mg), amide **53d** (1 mmol, 276 mg), TBAB (0.01 mmol, 0.32 mg), DIPEA (1 mmol, 174 μL), DCM 5 mL. The obtained crude product was purified by column chromatography over silica gel (DCM/Ace = 7:3) to yield **55d** (132 mg, 30%, *R*_*f*_ = 0.75 or 0.56 (S_12_)). ^1^H NMR (chloroform-*d*): δ ppm 7.76 (s, 1H, CON*H*), 7.29–6.82 (m, 12H, Ar), 5.71 (s, 1H, C*H*=C), 4.41–4.18 (m, 2H, NHC*H*_2_Ar,), 3.96 (dd, 1H, *J* = 11.2, 7.7 Hz, C*H*_2_′OH), 3,82 (dd, 1H, C*H*_2_′OH, *J* = 11.2, 4.2 Hz), 3.31 (s, 1H, C*H*N), 2,96 (d, 1H, *J* = 13,2 Hz, ArCH_2_C*H*_2_′Ar), 2.84–2.71 (m, 3H, ArC*H*_2_′C*H*_2_″Ar), 2.69–2.50 (m, 2H, C*H*_2_CH_2_CH=), 2.39–2.22 (m, 2H, CH_2_C*H*_2_CH=), 2.15 (s, 3H, CH_3_). OH proton was not detected. ^13^C NMR (chloroform-*d*): δ ppm 173.66 (NHCO), 162.06 (d, ^1^*J*_*C–F*_ = 245.6 Hz), 139.73 (CH=*C*), 136.92 (*C*_Ar_), 136.96 (*C*_Ar_), 134.01 (*C*_Ar_), 133,97 (d, ^4^*J*_*C–F*_ = 3,3 Hz), 130.18 (*C*_Ar_), 129.29 (*C*_Ar_), 129.18 (*C*_Ar_), 128,17 (*C*_Ar_), 128,10 (d, ^3^*J*_*C–F*_ = 8,3 Hz), 127.63 (*C*_Ar_), 126.14 (C=*C*H), 115.45 (d, ^2^*J*_*C–F*_ = 21.6 Hz), 67.48 (*C*HN), 57.90 (*C*H_2_OH), 53,20 (*C*H_2_CH_2_CH=), 42.11 (NH*C*H_2_Ar), 38.94 (*C*H_3_N), 33.70 (Ar*C*H_2_–CH_2_Ar), 32.00 (Ar*C*H_2_–CH_2_Ar), 28.10, (CH_2_*C*H_2_CH=). HRMS-ESI^+^*m*/*z* [M + H]^+^ calcd for C_29_H_32_FN_2_O_2_, 459.2440; found, 459.2448.

##### 4.1.1.7.13 2-((3-(10,11-Dihydro-5*H-*dibenzo[*a*,*d*][7]annulen-5-ylidene)propyl)(methyl)amino)-3-hydroxy-*N-*(4-methylbenzyl)propanamide **(56e**)

The synthesis was done according to GP7 with amine **C** (**21**) (1.2 mmol, 315 mg), amide **53e** (1 mmol, 271 mg), TBAB (0.01 mmol, 0.32 mg), DIPEA (1 mmol, 174 μL), DCM 5 mL. The obtained crude product was purified by column chromatography over silica gel (DCM/Ace = 7:3) to yield **55e** (136 mg, 30%, *R*_*f*_ = 0.70 or 0.57 (S_12_)). ^1^H NMR (chloroform-*d*): δ ppm 7.49 (s, 1H, CON*H*), 7.39–6.84 (m, 12H, Ar), 5.75 (t, 1H, *J* = 7.3 Hz, C*H*=C), 4.35 (d, 2H, *J* = 6.0 Hz, NHC*H*_2_Ar), 4.04–3.72 (m, 2H, C*H*_2_OH), 3,47–3.11 (m, 1H, C*H*N), 2.98–2.91 (m, 2H, ArCH_2_C*H*_2_′Ar), 2.75 (dd, 2H, *J* = 12.6, 6.3 Hz, ArCH_2_C*H*_2_″Ar), 2.56 (ddd, 2H, *J* = 11.0, 7.6, 3.7 Hz, C*H*_2_CH_2_CH=), 2.34–2.24 (m, 5H, CH_2_C*H*_2_CH=, Ar–C*H*_3_), 2.16 (s, 3H, CH_3_). OH proton was not detected. ^13^C NMR (chloroform-*d*): δ ppm 172.82 (NH*C*O), 144.41 (*C*_Ar_), 140.94 (CH=*C*), 139.74(*C*_Ar_), 139.23 (*C*_Ar_), 137.08 (*C*_Ar_), 137.01 (*C*_Ar_-CH_3_), 135.03 (*C*_Ar_), 130.02 (*C*_Ar_), 129.29 (*C*_Ar_), 128.44 (*C*_Ar_), 128.29(*C*_Ar_), 128,09 (*C*_Ar_), 128,01 (*C*_Ar_), 127.65 (*C*_Ar_), 127.55 (*C*_Ar_), 127.17 (*C*_Ar_), 126.05 (*C*_Ar_), 125.75 (*C*H=C), 66.90 (*C*HN), 59.98 (*C*H_2_OH), 57.34 (*C*H_2_CH_2_CH=), 42.64 (NH*C*H_2_Ar), 41.25 (*C*H_3_N), 33.69 (Ar*C*H_2_-CH_2_Ar), 32.00 (Ar*C*H_2_-CH_2_Ar), 27.16 (CH_2_*C*H_2_CH=), 21.08 (C_Ar_-*C*H_3_)

##### 4.1.1.7.14 *N-*Benzyl-2-[4-(9*H-*fluoren-9-ylidene)piperidin-1-yl]-3-hydroxypropanamide (**57**)

The synthesis was done according to GP7 with amine **D** (**25**) (0.49 mmol, 120 mg), amide 53a (0.40 mmol, 103 mg), TBAB (0.04 mmol, 13 mg), DIPEA (0.40 mmol, 70 μL), and DMF (2 mL). The obtained crude product was purified by column chromatography over silica gel (DCM/Ace = 7:3) to yield **57** (55 mg, 33%, *R*_*f*_ = 0.33 and 0.45 (DCM/Ace 7:3)) as a yellow oil. Formula C_28_H_28_N_2_O_2._ FW: 424.54. ^1^H NMR (300 MHz, chloroform-*d*) δ ppm 7.63–7.91 (m, 3H), 7.17–7.42 (m, 11H), 5.88–6.00 (m, 1H), 4.50 (d, *J* = 2.93 Hz, 1H), 4.42 (s, 1H), 3.82–4.05 (m, 2H), 3.67–3.83 (m, 1H), 3.18–3.59 (m, 2H), 2.76–3.04 (m, 2H), 2.57 (s, 1H), 2.38–2.49 (m, 1H), 1.20–1.50 (m, 2H).

##### 4.1.1.7.15 *N-*Benzyl-2-(4-(10,11-dihydro-5*H-*dibenzo[*a*,*d*][7]annulen-5-ylidene)piperidin-1-yl)-3-hydroxypropanamide (**58**)

The synthesis was done according to GP7 with amine **E** (**26**) (0.70 mmol, 290 mg), amide **53a** (0.58 mmol, 150 mg), TBAB (0.06 mmol, 19 mg), DIPEA (0.58 mmol, 100 μL), and DMF (3 mL). The obtained crude product was purified by column chromatography over silica gel (DCM/Ace = 7:3) to yield **58** (105 mg, 40%, *R*_*f*_ = 0.22 and 0.54 (DCM/Ace 7:3)) as a yellow oil. Formula C_30_H_32_N_2_O_2_, FW: 452.60. ^1^H NMR (300 MHz, chloroform-*d*) δ ppm 7.57–7.66 (m, 1H), 7.21–7.33 (m, 5H), 7.07–7.19 (m, 6H), 7.01 (d, *J* = 7.03 Hz, 2H), 4.44 (d, *J* = 5.27 Hz, 3H), 3.21–3.43 (m, 3H), 3.06–3.18 (m, 2H), 2.77–3.02 (m, 3H), 2.49–2.72 (m, 6H).

##### 4.1.1.7.16 *N-*Benzyl-2-((2-(((diphenylmethylene)amino)oxy)ethyl)(methyl)amino)-3-hydroxypropanamide (**59a**)

The synthesis was done according to GP7 with amine **F** (**32**) (1.2 mmol, 305 mg), amide **53a** (1 mmol, 257 mg), TBAB ((0.01 mmol, 0.32 mg), DIPEA (1 mmol, 174 μL), DMF 5 mL. The obtained crude product was purified by column chromatography over silica gel (DCM/Ace = 7:3) to yield **59a** (130 mg, 30%, *R*_*f*_ = 0.58 (S_12_)) ^1^H NMR (chloroform-*d*): δ ppm 7.71 (s, 1H, N*H*), 7.49–7.11 (m, 13H, Ar), 7.12–7.00 (m, 2H, Ar), 4.46–4.28 (m, 1H, CH_2_C*H*_2_′ON=), 4.27–4.13 (m, 2H, C*H*_2_NH), 4.13–4.03 (m, 1H, CH_2_C*H*_2_″ON=), 3,99–3.88 (m, 1H, C*H*_2_′OH), 3,79 (dd, 1H, CHNCH_3_, *J* = 11.4; 4.8 Hz), 3.26 (dd, 1H, C*H*_2_″OH, *J* = 7.0; 4.7 Hz,), 2.89 (td, 2H, C*H*_2_C*H*_2_′ON=), 2.34 (s, 3H, NC*H*_3_). OH proton was not detected. ^13^C NMR (chloroform-*d*): δ ppm 173.46 (*C*ONH), 157.23 (Ph_2_*C*=N), 138.07 (*C*_Ar_), 136.09 (*C*_Ar_), 133.08 (*C*_Ar_), 129.47(*C*_Ar_), 129.03 (*C*_Ar_), 128.99 (*C*_Ar_), 128.90 (*C*_Ar_), 128.67 (*C*_Ar_), 128.58 (*C*_Ar_), 128.49 (*C*_Ar_), 128.25 (*C*_Ar_), 128.11 (*C*_Ar_), 128.08 (*C*_Ar_), 127.80 (*C*_Ar_), 127.77 (*C*_Ar_), 127.55 (*C*_Ar_), 127.48 (*C*_Ar_), 127.18 (*C*_Ar_), 72.24 (*C*HN), 67.38 (CH_2_*C*H_2_ON=), 58.43 (*C*H_2_OH), 53.77 (*C*H_2_CH_2_ON=), 42.67 (*C*H_2_NH), 38.87 (*C*H_3_N) HRMS-ESI^+^*m*/*z* [M + H]^+^ calcd for C_26_H_30_N_3_O_3_, 432.2287; found, 432,2283.

##### 4.1.1.7.17 (2-Chlorobenzyl)-2-((2-(((diphenylmethylene)amino)oxy)ethyl)(methyl)amino)-3-hydroxypropanamide (**59b**)

The synthesis was done according to GP7 with amine **F** (**32**) (1.2 mmol, 305 mg), amide **53b** (1 mmol, 290 mg), TBAB ((0.01 mmol, 0.32 mg), DIPEA (1 mmol, 174 μL), DMF 5 mL. The obtained crude product was purified by column chromatography over silica gel (DCM/Ace = 7:3) to yield **59b** (130 mg, 30%, *R*_*f*_ = 0.60 (S_12_)). ^1^H NMR (300 MHz, chloroform-*d*) δ ppm 2.39 (s, 3 H, NC*H*_3_) 2.89–3.00 (m, 2 H, C*H*_2_CH_2_ON=) 3.33 (dd, *J* = 7.62. 4.10 Hz, 1 H, C*H*NCH_3_) 3.82 (dd, *J* = 11.14, 4.69 Hz, 1 H, C*H*′H″OH) 3.92–4.02 (m, 1 H, CH′*H*″OH) 4.14–4.35 (m, 4 H, CH_2_C*H*_2_ON=, NHC*H*_2_Ar) 7.13–7.50 (m, 14 H, Ar) 7.73 (t, *J* = 5.86 Hz, 1 H, N*H*CO). HRMS-ESI^+^*m*/*z* [M + H]^+^ calcd for C_26_H_29_N_3_O_3_Cl, 466.1897; found, 466.1900.

##### 4.1.1.7.18 2-((2-(((Diphenylmethylene)amino)oxy)ethyl)(methyl)amino)-*N-*(4-fluorobenzyl)-3-hydroxypropanamide (**59c**)

The synthesis was done according to GP7 with amine **F** (**32**) (1.2 mmol, 305 mg), amide **53c** (1 mmol, 276 mg), TBAB ((0.01 mmol, 0.32 mg), DIPEA (1 mmol, 174 μL), DMF 5 mL. The obtained crude product was purified by column chromatography over silica gel (DCM/Ace = 7:3) to yield **59c** (130 mg, 30%, *R*_*f*_ = 0.57 (S_12_)). ^1^H NMR (300 MHz, chloroform-*d*) δ ppm 2.39 (d, *J* = 1.76 Hz, 3 H, NC*H*_3_) 2.84–2.99 (m, 2 H, C*H*_2_CH_2_ON=) 3.32 (dt, *J* = 7.18, 3.74 Hz, 1 H, C*H*NCH_3_) 3.83 (dd, *J* = 11.14, 4.10 Hz, 1 H, C*H*′H″OH) 3.92–4.03 (m, 1 H, CH′*H*″OH) 4.04–4.22 (m, 2 H, CH_2_C*H*_2_ON=) 4.22–4.31 (m, 2 H, NHC*H*_2_Ar) 6.89–7.50 (m, 14 H, Ar) 7.68 (d, *J* = 5.27 Hz, 1 H, N*H*CO)). HRMS-ESI^+^*m*/*z* [M + H]^+^ calcd for C_26_H_29_N_3_O_3_F, 450,2193; found, 450,2189.

##### 4.1.1.7.19 *N-*Benzyl-2-bromopropanamide (**62**)

The synthesis was done according to GP8 *N-*benzylamine (8.8 mmol, 962 μL) with 2-bromopropanoic acid (**60**) (8 mmol, 724 μL), TEA (9.6 mmol, 1.24 mL), T3P (8 mmol, 4.76 mL), and DCM (32 mL) to yield **62** (1.64 g, 85%, *R*_*f*_ = 0.65 (S_1_). Formula: C_10_H_12_BrNO (241.01 g/mol). ^1^H NMR and ^13^C NMR consistent with literature data^59,60^ (300 MHz, chloroform-*d*) δ ppm 7.23–7.41 (m, 5H), 6.71 (br s, 1H), 4.44–4.50 (m, 2H), 4.11 (q, *J* = 7.18 Hz, 1H), 1.91 (d, *J* = 6.92 Hz, 3H).

##### 4.1.1.7.20 *N-*Benzyl-2-bromobutanamide (**63a**)

The synthesis was done according to GP8 *N-*benzylamine (8.8 mmol, 962 μL) with 2-bromopropanoic acid (**61**) (8 mmol, 850 μL), TEA (9.6 mmol, 1.24 mL), T3P (8 mmol, 4.76 mL), and DCM (32 mL) to yield **63** (1.63g, 80%, *R*_*f*_ = 0.60 (S_1_)). Formula: C_11_H_14_BrNO (255.01 g/mol). ^1^H NMR and ^13^C NMR consistent with literature data.^59,60^^1^H NMR (300 MHz, chloroform-*d*) δ ppm 7.30–7.42 (m, 3H), 7.16–7.26 (m, 2H), 6.72 (br s, 1H), 4.40–4.51 (m, 2H), 4.32 (dd, *J* = 5.00, 7.57 Hz, 1H), 1.93–2.28 (m, 2H), 0.98–1.10 (m, 3H).

##### 4.1.1.7.21 2-Bromo-*N-*(2-chlorobenzyl)butanamide (**63b**)

The synthesis was done according to GP8 (2-chlorophenyl)methanamine (8.8 mmol, 1062 μL) with 2-bromopropanoic acid (**61**) (8 mmol, 850 μL), TEA (9.6 mmol, 1.24 mL), T3P (8 mmol, 4.76 mL), and DCM (32 mL) to yield **63** (1.9 g, 82%, *R*_*f*_ = 0.7 (S_1_)). Formula: C_11_H_13_BrClNO (288.98 g/mol). ^1^H NMR (500 MHz, chloroform-*d*) δ ppm 7.35–7.42 (m, 2H), 7.20–7.27 (m, 2H), 6.94 (br s, 1H), 4.55 (d, *J* = 6.30 Hz, 2H), 4.34 (dd, *J* = 5.01, 7.59 Hz, 1H), 2.13–2.22 (m, 1H), 2.02–2.10 (m, 1H), 0.97–1.09 (m, 3H). ^13^C NMR (126 MHz, chloroform-*d*) δ ppm 168.6. 134.94, 133.63, 129.7, 129.14, 129.4, 53.6, 42.1, 29.3, 11.7

#### 4.1.1.8 General Procedure for the Synthesis of 2-Substituted Propanamide Derivatives **64a**,**b**, **65**, **66a**,**b**, **67**, **68**

The general procedure is corresponding to the GP7 method (general procedures for the synthesis of 2-substituted 3-hydroxypropanamide derivatives described in section 4.1.1.7).

##### 4.1.1.8.1 *N-*Benzyl-2-(4-(bis(3-methylthiophen-2-yl)methylene)piperidin-1-yl)butanamide(**64a**)

The synthesis was done according to GP7 with amine **A(17**) **(**0.48 mmol, 135 mg), amide **63a** (0.4 mmol, 102 mg), TBAB (0.04 mmol, 0.12 mg), TEA (0.4 mmol, 55 μL), DCM 0.5 mL. The obtained crude product was purified by column chromatography over silica gel (DCM/Ace = 7:3) to yield **59c** (101 mg, 55%, *R*_*f*_ = 0.15 (S_13_)). ^1^H NMR (300 MHz, chloroform-*d*) δ 7.23–7.43 (m, 9H), 7.12 (d, *J* = 4.69 Hz, 2H), 6.78 (d, *J* = 5.27 Hz, 2H), 5.31–5.31 (m, 1H), 5.32 (t, *J* = 5.57 Hz, 1H), 4.36–4.50 (m, 4H), 2.89 (t, *J* = 6.15 Hz, 1H), 2.46–2.69 (m, 4H), 2.24–2.33 (m, 4H), 2.10 (s, 6H), 1.67–1.81 (m, 2H), 0.89–1.03 (m, 5H). ^13^C NMR (75 MHz, chloroform-*d*) δ 173.0, 168.9, 159.4, 143.6, 138.0, 133.6, 128.1, 124.8, 124.3, 123.8, 119.7, 74.5, 70.1, 51.5, 43.2, 31.9, 25.1, 21.3, 14.5, 11.3, 8.9.

##### 4.1.1.8.2 2-(4-(Bis(3-methylthiophen-2-yl)methylene)piperidin-1-yl)-*N-*(2-chlorobenzyl)butanamide (**64b**)

The synthesis was done according to GP7 with amine **A(17**) (0.48 mmol, 135 mg), amide **63b** (0.4 mmol, 115 mg), TBAB (0.04 mmol, 0.12 mg), TEA (0.4 mmol, 55 μL), DCM 0.5 mL. The obtained crude product was purified by column chromatography over silica gel (DCM/Ace = 7:3) to yield **64b** (119 mg, 60%, *R*_*f*_ = 0.25 (S_13_)). ^1^H NMR (300 MHz, chloroform-*d*) δ ppm 0.89–1.02 (m, 3 H, CH_2_C*H*_3_) 1.67–1.78 (m, 2 H, C*H*_2_CH_3_) 2.10 (s, 6 H, tiophC*H*_3_) 2.25–2.35 (m, 4 H, Ppd) 2.42–2.53 (m, 2 H, Ppd) 2.54–2.64 (m, 2 H, Ppd) 2.87 (t, *J* = 6.15 Hz, 1 H, C*H*NCH_3_) 4.46–4.59 (m, 2 H, NHC*H*_2_Ar) 6.73–6.81 (m, 2 H, Ar) 7.12 (d, *J* = 5.27 Hz, 2 H, Tiof) 7.18–7.27 (m, 3 H, Tiof, Ar) 7.31–7.42 (m, 3 H, Ar) 7.60 (br s, 1 H, N*H*CO).

##### 4.1.1.8.3 *N-*Benzyl-2-((2-(((diphenylmethylene)amino)oxy)ethyl)(methyl)amino)propanamide (**65**)

The synthesis was done according to GP7 with amine **F** (**32**) (0.48 mmol, 122 mg), amide **62** (0.4 mmol, 96 mg), TBAB (0.04 mmol, 0.12 mg), TEA (0.4 mmol, 55 μL), DCM 0.5 mL. The obtained crude product was purified by column chromatography over silica gel (DCM/Ace = 7:3) to yield **65** (191 mg, 55%, *R*_*f*_ = 0.3 (S_13_)). ^1^H NMR (300 MHz, chloroform-*d*) δ ppm 1.22 (d, *J* = 7.03 Hz, 3 H, COCHC*H*_3_) 1.48–1.59 (m, 2 H,) 2.26 (s, 3 H) 2.76 (t, *J* = 5.57 Hz, 2 H) 3.27 (q, *J* = 7.03 Hz, 1 H) 4.09–4.31 (m, 4 H) 4.46 (d, *J* = 5.86 Hz, 1 H) 7.06–7.52 (m, 16 H) 7.57–7.70 (m, 1 H). ^13^C NMR (75 MHz, chloroform-*d*) δ 173.8, 159.2, 138.7, 136.1, 129.3, 129.0, 128.3, 128.1, 127.9, 127.7, 127.2, 72.3, 63.0, 53.3, 42.7, 38.4. HRMS-ESI^+^*m*/*z* [M + H]^+^ calcd for C_26_H_30_N_3_O_2_, 416.2338; found, 416.2327.

##### 4.1.1.8.4 *N-*Benzyl-2-((2-(((diphenylmethylene)amino)oxy)ethyl)(methyl)amino)butanamide (**66a**)

The synthesis was done according to GP7 with amine **F** (**32**) (0.48 mmol, 122 mg), amide **63a** (0.4 mmol, 102 mg), TBAB (0.04 mmol, 0.12 mg), TEA (0.4 mmol, 55 μL), DCM 0.5 mL. The obtained crude product was purified by column chromatography over silica gel (DCM/Ace = 7:3) to yield **66a** (106 mg, 62%, *R*_*f*_ = 0.3 (S_13_)). ^1^H NMR (300 MHz, chloroform-*d*) δ ppm 0.98 (t, *J* = 7.33 Hz, 3 H, CH_2_C*H*_3_) 1.58–1.76 (m, 1 H, C*H*′H″CH_3_) 1.78–1.92 (m, 1 H, CH′*H*″CH_3_) 2.30 (s, 3 H, NC*H*_3_) 2.75–2.92 (m, 2 H, C*H*_2_CH_2_ON=) 3.00 (t, *J* = 6.45 Hz, 1 H, C*H*NCH_3_) 4.05–4.17 (m, 1 H, CH_2_C*H*′H″ON=) 4.21–4.28 (m, 2 H, NHC*H*_2_Ar) 4.30–4.52 (m, 1 H, CH_2_CH′*H*″ON=) 7.07–7.50 (m, 16 H, Ar, N*H*CO). ^13^C NMR (75 MHz, chloroform-*d*) δ 173.33, 156.90, 138.86, 136.26, 133.22, 129.40, 129.11, 128.86, 128.66, 128.44, 128.27, 128.10, 127.83, 127.44, 127.03, 72.69, 69.51, 54.23, 42.80, 38.53, 20.67, 11.87. HRMS-ESI^+^*m*/*z* [M + H]^+^ calcd for C_27_H_32_N_3_O_2_, 430.2495; found, 430.2494.

#### 4.1.1.9 General Procedure for the Synthesis of *N-*Benzyl-2-bromopropanamide and *N-*Benzyl-2-bromobutanamide Derivatives **62**, **63a**,**b** (GP8)

Under an argon atmosphere, 2-bromopropanoic acid (**60**) or 2-bromobutanoic acid (**61**)(1 equiv) was dissolved in anhydrous DCM, and *n*-propanephosphonic anhydride (T_3_P, 50% in EtOAc, 1 equiv) was added. Acid activation was carried out for 30 min at −17° C, and then TEA (1.2 equiv) and the appropriate *N-*benzylamine (1.1 equiv) were added dropwise to the mixture. The mixture was warmed up to room temperature and stirred for 2 h. The solvent was evaporated, maintaining the water bath at a temperature below 30 °C. The residue was purified by column chromatography over silica gel (PE/EtOAc 7:3).

##### 4.1.1.9.1 *N-*(2-Chlorobenzyl)-2-((2-(((diphenylmethylene)amino)oxy)ethyl)(methyl)amino)butanamide (**66b**)

The synthesis was done according to GP7 with amine **F** (**32**) (0.48 mmol, 122 mg), amide **63b** (0.4 mmol, 115 mg), TBAB (0.04 mmol, 0.12 mg), TEA (0.4 mmol, 55 μL), DCM 0.5 mL. The obtained crude product was purified by column chromatography over silica gel (DCM/Ace = 7:3) to yield **66b** (133 mg, 60%, *R*_*f*_ = 0.35 (S_13_)). ^1^H NMR (300 MHz, chloroform-*d*) δ ppm 0.96 (t, *J* = 7.33 Hz, 3 H, CH_2_C*H*_3_) 1.58–1.74 (m, 1 H, C*H*′H″CH_3_) 1.78–1.93 (m, 1 H, CH′*H*″CH_3_) 2.30 (s, 3 H, NC*H*_3_) 2.78–2.94 (m, 2 H, C*H*_2_CH_2_ON=) 3.01 (t, *J* = 6.45 Hz, 1 H, C*H*NCH_3_) 4.07–4.23 (m, 1 H, CH_2_C*H*′H″ON=) 4.23–4.33 (m, 2 H, NHC*H*_2_Ar) 4.33–4.62 (m, 1 H, CH_2_CH′*H*″ON=) 7.08–7.51 (m, 15 H, Ar, N*H*CO). ^13^C NMR (75 MHz, chloroform-*d*) δ 173.53, 156.93, 140.43, 136.24, 136.12, 133.15, 130.22, 129.42, 129.38, 129.27, 129.11, 129.08, 128.84, 128.34, 128.23, 128.04, 127.81, 126.82, 72.74, 69.36, 54.29, 40.87, 38.48, 20.50, 12.02. HRMS-ESI^+^*m*/*z* [M + H]^+^ calcd for C_27_H_31_N_3_O_2_Cl, 464.2105; found, 464.2104.

##### 4.1.1.9.2 *N-*Benzyl-2-((4,4-bis(3-methylthiophen-2-yl)but-3-en-1-yl)(methyl)amino)propanamide (**67**)

The synthesis was done according to GP7 with amine **G** (**33**) (0.48 mmol, 133 mg), amide **62** (0.4 mmol, 96 mg), TBAB (0.04 mmol, 0.12 mg), TEA (0.4 mmol, 55 μL), DCM 0.5 mL. The obtained crude product was purified by column chromatography over silica gel (DCM/Ace = 7:3) to yield **67** (105 mg, 60%, *R*_*f*_ = 0.35 (S_13_)). ^1^H NMR (300 MHz, chloroform-*d*) δ ppm 1.17–1.26 (m, 3 H), 1.90 (s, 3 H), 2.00 (s, 3 H), 2.12 (s, 3 H), 2.23–2.31 (m, 2 H), 2.47–2.59 (m, 2 H), 3.22 (q, *J* = 6.45 Hz, 1 H), 4.29–4.52 (m, 2 H), 5.99 (t, *J* = 7.33 Hz, 1 H), 6.70–6.77 (m, 1 H), 6.83 (d, *J* = 4.69 Hz, 1 H), 7.02 (d, *J* = 5.28 Hz, 1 H), 7.18–7.32 (m, 6 H), 7.62 (br s, 1 H). ^13^C NMR (75 MHz, chloroform-*d*) δ 183.1, 131.3, 129.6, 128.5, 127.7, 127.2, 124.4, 122.6, 62.9, 54.1, 43.1, 37.8, 28.2, 14.7, 14.4, 9.6. HRMS-ESI^+^*m*/*z* [M + H]^+^ calcd for C_25_H_30_N_2_OS_2_, 439.1872; found, 439.1877.

##### 4.1.1.9.3 *N-*Benzyl-2-((4,4-bis(3-methylthiophen-2-yl)but-3-en-1-yl)(methyl)amino)butanamide (**68**)

The synthesis was done according to GP7 with amine **G** (**33**) (0.48 mmol, 133 mg), amide **63a** (0.4 mmol, 105 mg), TBAB (0.04 mmol, 0.12 mg), TEA (0.4 mmol, 55 μL), DCM 0.5 mL. The obtained crude product was purified by column chromatography over silica gel (DCM/Ace = 7:3) to yield **68** (90 mg, 50%, *R*_*f*_ = 0.35 (S_13_)). ^1^H NMR (300 MHz, chloroform-*d*) δ ppm 0.97 (t, *J* = 7.33 Hz, 3 H, CH_2_C*H_3_*) 1.56–1.87 (m, 3 H, thiopC*H*_3_) 1.88–1.92 (m, 3 H, thiopC*H*_3_) 1.98–2.04 (m, 3 H, NC*H*_3_) 2.18 (s, 2 H, C*H*_2_CH=) 2.28 (q, *J* = 7.03 Hz, 2 H) 2.54–2.68 (m, 2 H, C*H*_2_CH_2_CH=) 2.95 (d, *J* = 18.17 Hz, 1 H, C*H*NCH_3_) 4.33–4.55 (m, 2 H, NHC*H*_2_Ar) 5.98 (t, *J* = 7.33 Hz, 1 H, C*H*=) 6.74 (d, *J* = 5.27 Hz, 1 H, thiop) 6.83 (d, *J* = 5.27 Hz, 1 H, thiop) 6.98–7.08 (m, 1 H, thiop) 7.15–7.38 (m, 6 H, Ar, thiop) 7.44 (br s, 1 H, N*H*CO). ^13^C NMR HRMS-ESI^+^*m*/*z* [M + H]^+^ calcd for C_26_H_32_N_2_OS_2_, 453.2029; found, 453.2015

#### 4.1.1.10 General Procedure for the Synthesis of Ethyl 2-Substituted Propanoate **71**, **73** and Benzyl 2-Substituted Butanoate **72** (GP11)

The corresponding amine **B** or **G** (1.2 equiv), a relevant ester **69** or **70** (1 equiv), and K_2_CO_3_ (3.5 equiv) in DCM was stirred at 40 °C (oil bath) for 12 h. The mixture was extracted with 2 M HCl and DCM. Organic phases were dried with anhydrous Na_2_SO_4_ and concentrated, and the residue was purified by column chromatography over silica gel (S_1_ PE/EtOAc 7:3, v/v).

##### 4.1.1.10.1 Ethyl *N-*(3-(5*H-*Dibenzo[*a*,*d*][7]annulen-5-ylidene)propyl)-*N-*methylalaninate (**71**)

The synthesis was done according to GP11 with amine **B** (**20**) (0.48 mmol, 125 mg), ethyl 2-bromopropanoate (0.4 mmol, 52 μL), K_2_CO_3_ (194 mg, 1,4 mmol), DCM 0.5 mL. The obtained crude product was purified by column chromatography over silica gel (DCM/Ace = 7:3) to yield **71** (101 mg, 70%, *R*_*f*_ = 0.5 (S_1_). ^1^H NMR (300 MHz, chloroform-*d*) δ ppm 7.21–7.41 (m, 8H), 6.86 (d, *J* = 1.17 Hz, 2H), 5.57 (ddd, *J* = 3.52, 6.74, 7.91 Hz, 1H), 4.07–4.21 (m, 2H), 3.26–3.43 (m, 1H), 2.45–2.77 (m, 2H), 2.14–2.43 (m, 6H), 1.15–1.37 (m, 9H). ^13^C NMR (75 MHz, chloroform-*d*) δ 173.7, 142.7, 137.7, 134.3, 132.1, 131.5, 129.0, 128.3, 128.0, 127.2, 60.5, 54.4, 38.4, 27.8. HRMS-ESI^+^*m*/*z* [M + H]^+^ calcd for C_24_H_28_NO_2_, 362.2120; found, 362.2117.

##### 4.1.1.10.2 Benzyl *N-*(3-(5*H-*Dibenzo[*a*,*d*][7]annulen-5-ylidene)propyl)-*N-*methylalaninate (**72**)

The synthesis was done according to GP11 with amine **B** (**20**) (0.48 mmol, 125 mg), benzyl 2-bromopropanoate (0.4 mmol, 67 μL), K_2_CO_3_ (194 mg, 1,4 mmol), DCM 0.5 mL. The obtained crude product was purified by column chromatography over silica gel (DCM/Ace = 7:3) to yield **72** (1735 mg, 80%, *R*_*f*_ = 0.6 (S_1_). ^1^H NMR (300 MHz, chloroform-*d*) δ 7.19–7.42 (m, 13H), 6.86 (s, 2H), 5.49–5.62 (m, 1H), 5.09–5.16 (m, 2H), 3.33–3.49 (m, 1H), 2.46–2.74 (m, 2H), 2.13–2.41 (m, 6H), 1.27 (dd, *J* = 7.03, 12.89 Hz, 3H). HRMS-ESI^+^*m*/*z* [M + H]^+^ calcd for C_24_H_30_NO_2_, 424.2277; found, 424.2274

##### 4.1.1.10.3 Ethyl 2-((4,4-Bis(3-methylthiophen-2-yl)but-3-en-1-yl)(methyl)amino)propanoate (**73**)

The synthesis was done according to GP11 with amine **G** (**23**) (0.48 mmol, 133 mg), ethyl 2-bromopropanoate (0.4 mmol, 52 μL), K_2_CO_3_ (194 mg, 1,4 mmol), DCM 0.5 mL. The obtained crude product was purified by column chromatography over silica gel (DCM/Ace = 7:3) to yield **73** (106 mg, 70%, *R*_*f*_ = 0.5 (S_1_). ^1^H NMR (300 MHz, chloroform-*d*) δ ppm 1.20–1.34 (m, 6 H) 2.03 (d, *J* = 8.79 Hz, 6 H) 2.26–2.36 (m, 5 H) 2.48–2.77 (m, 2 H) 3.37 (q, *J* = 7.03 Hz, 1 H) 4.15 (q, *J* = 7.03 Hz, 2 H) 6.06 (t, *J* = 7.33 Hz, 1 H) 6.75 (d, *J* = 5.28 Hz, 1 H) 6.84 (d, *J* = 5.27 Hz, 1 H) 7.04 (d, *J* = 5.27 Hz, 1 H) 7.20 (d, *J* = 5.27 Hz, 1 H). HRMS-ESI^+^*m*/*z* [M + H]^+^ calcd for C_20_H_27_NO_2_S_2_, 378.1556; found, 378.1560.

#### 4.1.1.11 General Procedure for the Hydrolysis of Ethyl and Benzyl 2-Substituted Butanoate **71** and **73** to **74** and **75** (GP12)

An aqueous 10 wt % NaOH (0.15 g/1.5 mL) solution was added to compounds **71** and **73** (0.3 mmol) and stirred for 4.5 h at 35 °C. The aqueous phase was acidified to pH = 3 with aq HC1 (1 M) and extracted with DCM twice. The combined organic phases are dried over anhydrous Na_2_S0_4_, filtered, and then concentrated to give product.

##### 4.1.1.11.1 *N-*(3-(5*H-*Dibenzo[*a*,*d*][7]annulen-5-ylidene)propyl)-*N-*methylalanine (**74**)

The synthesis was done according to GP12 with ethyl *N-*(3-(5*H-*dibenzo[*a*,*d*][7]annulen-5-ylidene)propyl)-*N-*methylalaninate (**71**) (108 mg, 0.3 mmol) in 2 mL of 10 wt % NaOH to give the oil (quantitative) (100 mg). ^1^H NMR (300 MHz, chloroform-*d*) δ 7.20 (d, *J* = 2.34 Hz, 2H), 7.00–7.20 (m, 8H), 6.99–7.21 (m, 8H), 6.73–6.77 (m, 2H), 5.37 (t, *J* = 5.28 Hz, 1H), 3.14–3.35 (m, 1H), 2.53–3.03 (m, 3H), 2.33 (br s, 4H), 1.10–1.22 (m, 3H). COO*H* proton was not detected. HRMS-ESI^+^*m*/*z* [M + H]^+^ calcd for C_22_H_24_N_2_O, 334.1870; found, 334.1805

##### 4.1.1.11.2 2-((4,4-Bis(3-methylthiophen-2-yl)but-3-en-1-yl)(methyl)amino)propanoic Acid (**75**)

Ethyl 2-((4,4-bis(3-methylthiophen-2-yl)but-3-en-1-yl)(methyl)amino)propanoate (**73**) (113 mg, 0.3 mmol) in 2 mL 10 wt % NaOH was used, giving the oil (quantitative) (105 mg). ^1^H NMR (300 MHz, chloroform-*d*) δ 7.23 (d, *J* = 5.28 Hz, 1H), 7.07 (d, *J* = 5.27 Hz, 1H), 6.86 (d, *J* = 5.28 Hz, 1H), 6.76 (d, *J* = 5.28 Hz, 1H), 5.98 (t, *J* = 6.74 Hz, 1H), 3.50 (br s, 1H), 3.11 (br s, 1H), 2.95 (d, *J* = 6.45 Hz, 1H), 2.49–2.68 (m, 5H), 2.00–2.05 (m, 3H), 1.97 (s, 3H), 1.39 (d, *J* = 4.10 Hz, 3H). COO*H* proton was not detected.

### 4.2 *In Vitro* Activity

#### 4.2.1 [^3^H]GABA Uptake Assay

The inhibitory activities of the synthesized compounds were determined from [^3^H]GABA uptake assays with mGAT1, mGAT2, mGAT3, and mGAT4 as described previously,^19,61^ and all compounds were tested at a screening concentration of 100 μM.

#### 4.2.2 MS Binding Assays

MS binding assays for mGAT1 were performed as described earlier.^50^ Inhibition of mGAT1 binding by the synthesized compounds was determined in MS binding assays at a screening concentration of 100 μM and analyzed by LC–ESI-MS/MS.

### 4.3 Molecular Modeling

#### 4.3.1 Docking Studies

For the docking studies, we used models of human GAT-1, GAT-2, and GAT-3 that were selected in our previous work.^53^ They were built with the SWISS-MODEL server based on the 4XP9 template from the Protein Data Bank (PDB). For BGT-1 we decided to apply the extra model built on the same template, according to previously described procedure.^53^ In this model, the nonhelical fragment of TM10 (residues 455–459) was additionally optimized using the MyLoop class in the Modeller program. From among 100 refined models, the best one was selected according to the QMEAN score. For each type of GABA transporter we used sequence alignment generated automatically by SWISS-MODEL. The N- and C-termini were omitted because of their low homology. Sodium and chloride ions were transferred directly from the templates.

The ligand 3D structures were created in the Maestro program. Ionization states were predicted under physiological conditions (pH 7.4) using the Epik and Marvin programs. Ligands were optimized in the LigPrep module. All possible stereoisomers for each ligand were generated. Models were prepared with Protein Preparation Wizard using the default settings.

The most active compound representatives were initially docked into the models of each type of GABA transporter using the induced-fit docking protocol available in the Schrödinger Suite. The box center was defined by residues PHE294, TYR140, TYR452, and ARG69 in GAT-1 and by the corresponding amino acids in BGT-1, GAT-2, and GAT-3. The box size was 10 Å  ×  10 Å ×  10 Å. The obtained complexes were then visually inspected in terms of the created interactions, frequency, and score of the poses as well as their coherency between the particular types of GATs. After selection of the best optimized models, all studied compounds were docked into the models using the GLIDE program and the final conformations of the models were selected based on ligand pose coherency. The grid center in GLIDE was set as the centroid of the ligand from the complex, and the inner box size was 15 Å ×  15 Å  ×  15 Å. The OPLS2005 force field was applied during grid generation as well as GLIDE and IFD docking.

#### 4.3.2 Molecular Dynamics

MD simulations were performed with NAMD using the CHARMM36m force field. Before simulations, all models were positioned in the membrane using the OPM server, and input files for NAMD were prepared with the CHARMM-GUI online server. The protein–ligand complexes were embedded in a 1-palmitoyl-2-oleoylphosphatidylcholine (POPC) membrane and solvated with TIP3P water molecules. The system size was 100 Å  ×  100 Å. A water pore for each complex was generated. Sodium and chloride ions (0.15 M NaCl) were added to provide standard physiological ionic strength. The system was equilibrated via a six step protocol recommended by CHARMM-GUI for the NAMD program. MD simulations were run at 303.15 K with a time step of 2 fs and a total duration of 10 ns. The intervals for both the energy and trajectory recordings were 10 ps. The results were analyzed with the VMD program.

### 4.4 Hepatotoxicity and Cytotoxicity

Hepatotoxicity and cytotoxicity were estimated according to previously described protocols^62^ using the hepatoma HepG2 (ATCC HB-8065) and human embryonic kidney HEK-293 (ATCC CRL1573) cell lines, respectively. In brief, cells were seeded in 96-well plates at a density of 0.7 × 10^4^ and cultured at 37 °C in an atmosphere containing 5% CO_2_. Next, the compounds were added and investigated in quadruplicate at concentrations ranging from 0.1 to 100 μM for 72 h. The antiproliferative drug DX was used as the reference. The CellTiter 96 AQueous nonradioactive cell proliferation assay (MTS) purchased from Promega (Madison, WI, USA) was used for the determination of cell viability. The absorbance at 492 nm was measured using an EnSpire microplate reader (PerkinElmer, Waltham, MA, USA).

### 4.5 *In Vivo* Evaluation

#### 4.5.1 Materials and Methods

##### 4.5.1.1 Animals and Housing Conditions

Behavioral experiments were carried out at the Department of Pharmacodynamics, Faculty of Pharmacy, Jagiellonian University Medical College, Krakow. Tests were performed between 9 a.m. and 2 p.m. All experimental *in vivo* procedures were approved by the second Local Ethics Committee in Krakow (Approval Numbers 32/2018 and 508/2021). The treatment of animals was in full accordance with the ethical standards laid down by both Polish and EU regulations (Directive 2010/63/EU). To avoid potential bias in data recording, the investigators who were involved in behavioral assays were blinded to the experimental groups. Adult male albino Swiss (CD-1) mice weighing 18–22 g were supplied by the Animal Breeding Farm of the Jagiellonian University Faculty of Pharmacy. Before the *in vivo* tests, the mice were kept in groups of 10 in standard plastic cages. Bedding material (Transwiór, Poland) was at least 2 cm deep to allow the mice to dig, and animals were housed under controlled laboratory conditions (room temperature of 22 ± 2 °C, light/dark (12:12) cycle, lights on at 8 a.m., humidity 50 ± 10%, and free access to food (Murigran, Agropol, Poland) and tap water). Experimental groups consisted of 8–10 animals/dose. For behavioral tests, the mice were selected randomly After completion of the assays, the mice were euthanized by cervical dislocation.

##### 4.5.1.2 Chemicals Used in the *in Vivo* Tests

Before the *in vivo* tests, the test compounds were suspended in 1% Tween 80 (Baxter, Poland). The compounds were then administered intraperitoneally. The dose of 30 mg/kg of each compound was the starting dose, and if activity was observed in the pain tests, a dose of 10 mg/kg was also tested. The test compounds were administered only once daily on days 1 and 7 of oxaliplatin- or paclitaxel-induced neuropathy. In STZ-treated mice, test compounds were administered 21 days after STZ injection. Control mice used in oxaliplatin and paclitaxel NP models were injected with an appropriate amount of vehicle (0.9% saline). Oxaliplatin was purchased from Activate Scientific GmbH (Germany). Paclitaxel and STZ were purchased from Sigma-Aldrich (Poland).

##### 4.5.1.3 Induction of Neuropathy and NP

For pain studies, oxaliplatin was dissolved in 5% glucose solution (Polfa Kutno, Poland). The dose of oxaliplatin used to induce peripheral neuropathy (10 mg/kg, intraperitoneal injection) was chosen on the basis of previous research^63,64^ and available literature data.^65^

Doses of both paclitaxel and STZ used for the induction of neuropathy were selected based on our previous research.^66,67^ To induce neuropathy, paclitaxel was used at a dose of 18 mg/kg. It was prepared by dissolving in ethanol (100% (v/v); Polskie Odczynniki Chemiczne, Gliwice, Poland) at 10% of the final desired volume and vortexed for 2 min. An equal volume of Cremophor EL (10% of the final volume) was then added, and the mixture was vortexed for the next 10 min. Prior to injection, ice-cold physiological saline (80% of the final volume) was added to make up a final volume and the solution was maintained on ice during dosing.^66^ To induce type I diabetes, mice were intraperitoneally injected with STZ (a single injection of STZ, 200 mg/kg) dissolved in 0.1 N citrate buffer. Age-matched control mice received an equal volume of citrate buffer. Blood glucose level was measured 1 day before (referred to as “day 0”) and repeatedly 1, 2, and 3 weeks after STZ injection using a blood glucose monitoring system (Accu-Chek Active, Roche, France). Blood samples for measurement of glucose concentration were obtained from the tail vein of the mice. The animals were considered as diabetic when their blood glucose concentration exceeded 300 mg/dL,^68^ and only these mice (diabetic mice) were used in subsequent pain tests.^67^

##### 4.5.1.4 Assessment of Tactile (Mechanical) Allodynia (von Frey Test)

The ability of the test compounds to attenuate tactile allodynia caused by oxaliplatin, paclitaxel, and STZ was assessed using the von Frey test. For this purpose, 3 h after oxaliplatin or paclitaxel and 21 days after STZ injection, the predrug paw withdrawal threshold was measured for each mouse. Then, the test compounds were administered, and 1 h later, the postdrug paw withdrawal threshold was collected for each animal. This part of the experiment aimed to establish the effect of treatment on early phase (acute) pain hypersensitivity induced by cytotoxic drugs. Additionally, in the oxaliplatin model and the paclitaxel model, to assess the effect of test compounds on late phase tactile allodynia, 7 days later, measurements of the predrug and postdrug paw withdrawal thresholds were made in a similar manner to the measurements performed during early phase neuropathy. At this stage of the experiment, there was no additional oxaliplatin/paclitaxel administration.

An electronic von Frey unit (Bioseb, France) was used to assess the mechanical nociceptive threshold (tactile allodynia) in mice. This device has a single flexible filament that applies increasing force (from 0 to 10 g) against the plantar surface of the hind paw of each mouse. In the von Frey test, the paw withdrawal response of the animals automatically turns off the stimulus, and the mechanical pressure that evokes this response is recorded. On the day of the experiment, the mice were placed individually in test compartments with a wire mesh bottom and left there for 1 h of habituation. Subsequently, to obtain baseline values, each mouse was tested 3 times alternately in each hind paw. Then, the test compounds were administered, and 1 h later, 3 additional measurements were taken and averaged to obtain the mean postdrug values for each mouse.^69^

##### 4.5.1.5 Assessment of Cold Nociceptive Threshold (Cold Plate Test)

A cold plate apparatus (hot/cold plate, Bioseb, France) set at 2.5 °C was used to assess the effect of treatment on cold hyperalgesia in oxaliplatin-treated mice. The cold plate test was conducted immediately after the von Frey test. Three hours after oxaliplatin administration, the animals were placed on a cold plate apparatus, and predrug latencies to pain reaction (i.e., lifting, biting, shaking of hind paws, jumping, movement deficits, or writhing response) were collected. Finally, the test compounds were injected, and 1 h later, the postdrug latencies to pain reaction were measured. In this assay, a cutoff time of 60 s was established to avoid potential thermally induced damage to the paw tissues, and animals not responding within 60 s were removed from the apparatus and assigned a score of 60 s.^70,71^

##### 4.5.1.6 Assessment of Heat Nociceptive Threshold (Hot Plate Test)

Thermal (heat) nociceptive threshold was assessed in the hot plate test as previously described.^67^ First, baseline (predrug) latencies to pain reaction were established for each mouse. Then, the mice were treated intraperitoneally with either the test compound or vehicle. Sixty minutes later the animals were placed on the hot plate apparatus again (hot/cold plate, Bioseb, France). This apparatus has an electrically heated surface and is supplied with a temperature-controller that maintains the temperature at 55 °C. The time until the animal licked its hind paws or jumped was recorded by means of a stop-watch. In this assay a cutoff time was established (60 s) to avoid tissue damage, and the mice not responding within 60 s were removed from the apparatus and assigned a score of 60 s.

##### 4.5.1.7 Rotarod Test

Before the rotarod test, the experimental animals underwent 3 d of training on the rotarod apparatus (totarod apparatus, May Commat RR0711, Turkey; rod diameter 2 cm) that rotated at a fixed speed of 18 rotations per minute (rpm). During this training session, the mice were placed on the rotating rod for 3 min with an unlimited number of trials. The proper test was performed 24 h after the last training session. Sixty minutes after administration of the test compounds or vehicle, the mice were tested on rods that revolved at 6, 18, and 24 rpm. Motor deficits in the mice were defined as their inability to remain on the rotarod apparatus for 1 min. The results are expressed as the mean time spent on the rotarod.^70^

##### 4.5.1.8 Data Analysis

Data analysis of the *in vivo* results was performed using GraphPad Prism software (version 8.0, CA, USA). Numerical results obtained in behavioral tests are expressed as the mean ± SEM. Statistical analysis was performed using the Shapiro–Wilk normality test, followed by one-way analysis of variance (ANOVA) and Tukey’s post hoc comparison (NP models). One-way ANOVA and Dunnett’s post hoc comparison were used to test differences between the drug-treated groups and the control group in the rotarod test. A value of *p* < 0.05 was considered significant.

## Supporting Information Available

Synthesis of building blocks **17** (**A**), **20** (**B**), **21** (**C**), **25** (**D**), **26** (**E**), **32** (**F**), and **33** (**G**); RMSD changes over the course of the molecular dynamics simulations; ^1^H and ^13^C NMR spectra of synthesized compounds (PDF)

## Abbreviations

GABA: γ-aminobutyric acid
CNS: central nervous system
GABA_A_: γ-aminobutyric acid type A receptor
GABA_B_: γ-aminobutyric acid type B receptor
GABA_C_: γ-aminobutyric acid type C receptor
GAT: GABA transporter protein
SLC6: solute carrier 6 family
BGT1: betaine/GABA transporter 1
IUPHAR: International Union of Basic and Clinical Pharmacology
GAT1: GABA transporter 1
GAT2: GABA transporter 2
RMSD: root-mean-square deviation
GAT3: GABA transporter 3
NP: neuropathic pain
FDA: U.S. Food and Drug Administration
BOC: *tert*-butyloxycarbonyl protecting group
TEA: triethylamine
THF: tetrahydrofuran
DCM: dichloromethane
STZ: streptozotocin
