# Supplementary material for: Novel Functionalized Amino Acids as Inhibitors of GABA Transporters with Analgesic Activity
Source: ACS Chem Neurosci. 2021 Aug 4;12(16):3073–100. doi: 10.1021/acschemneuro.1c00351 (PMC8397297; doi:10.1021/acschemneuro.1c00351)
Supplement: Supplementary file 1 — cn1c00351_si_001.pdf [file cn1c00351_si_001.pdf]

Supporting information

Novel functionalized amino acids as inhibitors of GABA transporters with analgesic activity

Beata Gryzło[1], Paula Zaręba[1*], Katarzyna Malawska[1], Gabriela Mazur[1], Anna Rapacz[1], Kamil Łątka[1], Georg C. Höfner[2], Gniewomir Latacz[1], Marek Bajda[1], Kinga Sałat[1], Klaus T. Wanner[2], Barbara Malawska[1], Katarzyna Kulig[1](†)

[1]Faculty of Pharmacy, Jagiellonian University Medical College, 9 Medyczna St, 30–688 Kraków, Poland
[2]Department of Pharmacy, Center for Drug Research, Ludwig-Maximilians-Universität München Butenandtstr, 5-13, 81377 Munich, Germany.

(†) Author is deceased, †26.06.2016

[*]Corresponding author:

Paula Zaręba
9 Medyczna Street, 30–688 Kraków, Poland
E-Mail address: paula.zareba@uj.edu.pl

Contents of the supporting information:

1. Synthesis of building blocks **17** (A), **20** (B), **21** (C), **25** (D), **26** (E) and **32** (F); (Scheme S1) S2
2. RMSD changes over the course of the molecular dynamics simulations for the compounds (Panel A) and protein residues within 8Å of ligand (Panel B); (Figure S1) S4
3. ^{1}H and ^{13}C NMR spectra of synthesized compounds ((2*RS*,4*SR*)-**37b**, (2*RS*,4*SR*)-**37c**, (2*RS*,4*RS*)-**39b**, (2*RS*,4*RS*)-**39c**, (2*RS*,4*SR*)-**39c**, (2*RS*,4*RS*)-**43a**, (2*RS*,4*SR*)-**43a**, **47a**, **47b**, **47c**, **50a**, **50b**, **51a**, **54a**, **54b**, **54c**, **56a**, **58**, **59b**, **67**, **73** S8 – S27

1. RESULTS

1.1 Chemistry

1.1.1 Synthesis of building blocks 17 (A), 20 (B), 21 (C), 25 (D), and 26 (E)

To obtain building block **17**, we used the Grignard reaction between (3-methylthiophen-2-yl)magnesium bromide (**15**) and 1-(tert-butyl) 4-ethyl piperidine-1,4-dicarboxylate (**14**) [1]. A previously described strategy gave the corresponding compound **16** [2]. Cleavage and dehydration of the *N*-tert-butoxycarbonyl (Boc) group to yield target compound (**17**) was performed using p-toluenesulfonic acid based on literature data [3]. Building blocks with a tricyclic system (**20** [4] and **21**) were obtained using the corresponding alkyl halides (**18** and **19**) as described previously [2], and a methylamine solution [5]. Building blocks **25** and **26** were prepared via McMurry coupling using the appropriate tricyclic ketones (**22** or **23**) and tert-butyl 4-oxopiperidine-1-carboxylate (**24**) providing a convenient synthetic approach [6,7].

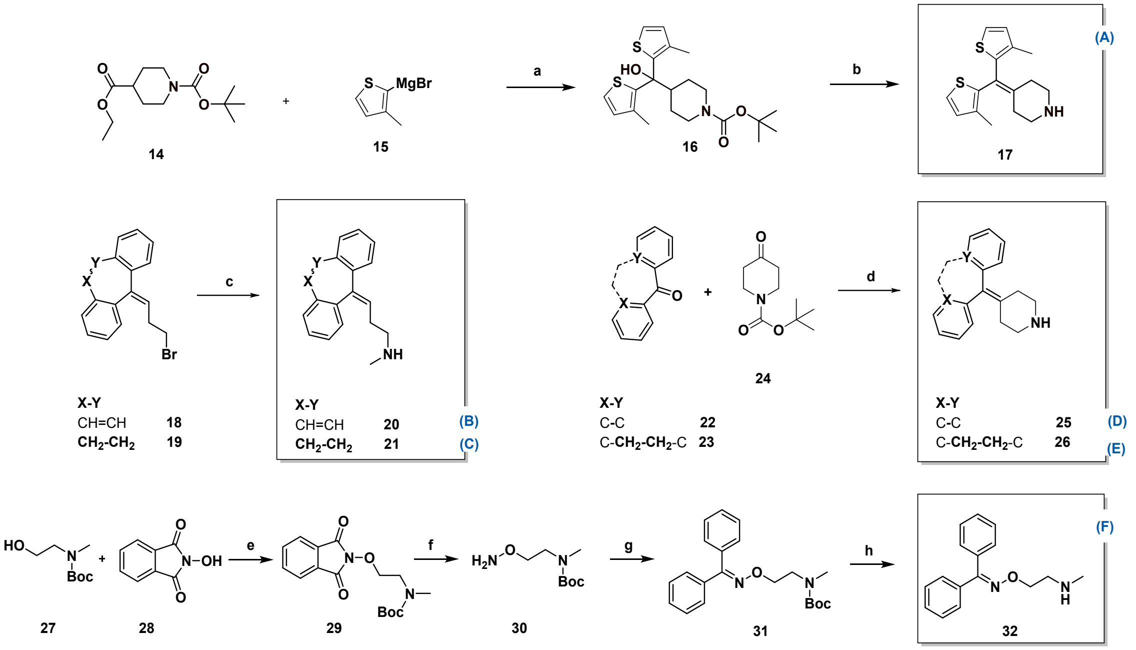

Scheme S1. Synthesis of the building blocks (**17** (A), **20** (B), **21** (C), **25** (D), **26** (E) and **32** (F)). Reagents and conditions: (a) dry THF, argon, room temperature (rt), 24 h; (b) p-toluenesulfonic acid, toluene, reflux, 12 h; (c) methylamine ($MeNH_2$) solution (33 wt. % in absolute ethanol), rt, 48 h; (d) $TiCl_4$, Zn, dry THF, argon, 0°C (10 min)/reflux (1.5 h); then 2 M HCl, toluene, rt, 5 min; (e) DIAD, PPh_3, dry THF, 0°C (1 h)/rt (1 h); (f) NH_2NH_2 (50%–60%), ethanol, rt, 1 h; and (g) NaOH, benzophenone, ethanol, 70°C, 30 min; (h) TFA, DCM, rt, 1 h.

1.1.2 Synthesis of building blocks 32 (F) and 33 (G)

Oxime formation proceeded in three steps. First, the Mitsunobu reaction between *N*-Boc-protected *N*-methylamino ethanol (**27**) and *N*-hydroxyphthalimide (**30**) was performed. Second, after hydrazine-promoted deprotection of compound **29**, an oxime moiety was formed (**30**). Compound **31** was formed from hydroxylamine (**30**) and benzophenone under strongly alkaline conditions [8,9]. Desired compound **32** was achieved after the deprotection of a BOC-protected amine **31**. The synthesis of *N*-methyl-4,4-bis(3-methylthiophen-2-yl)but-3-en-1-amine (**33** (**G**)) was carried out based on the literature data [10–13].

1.2 Molecular modeling

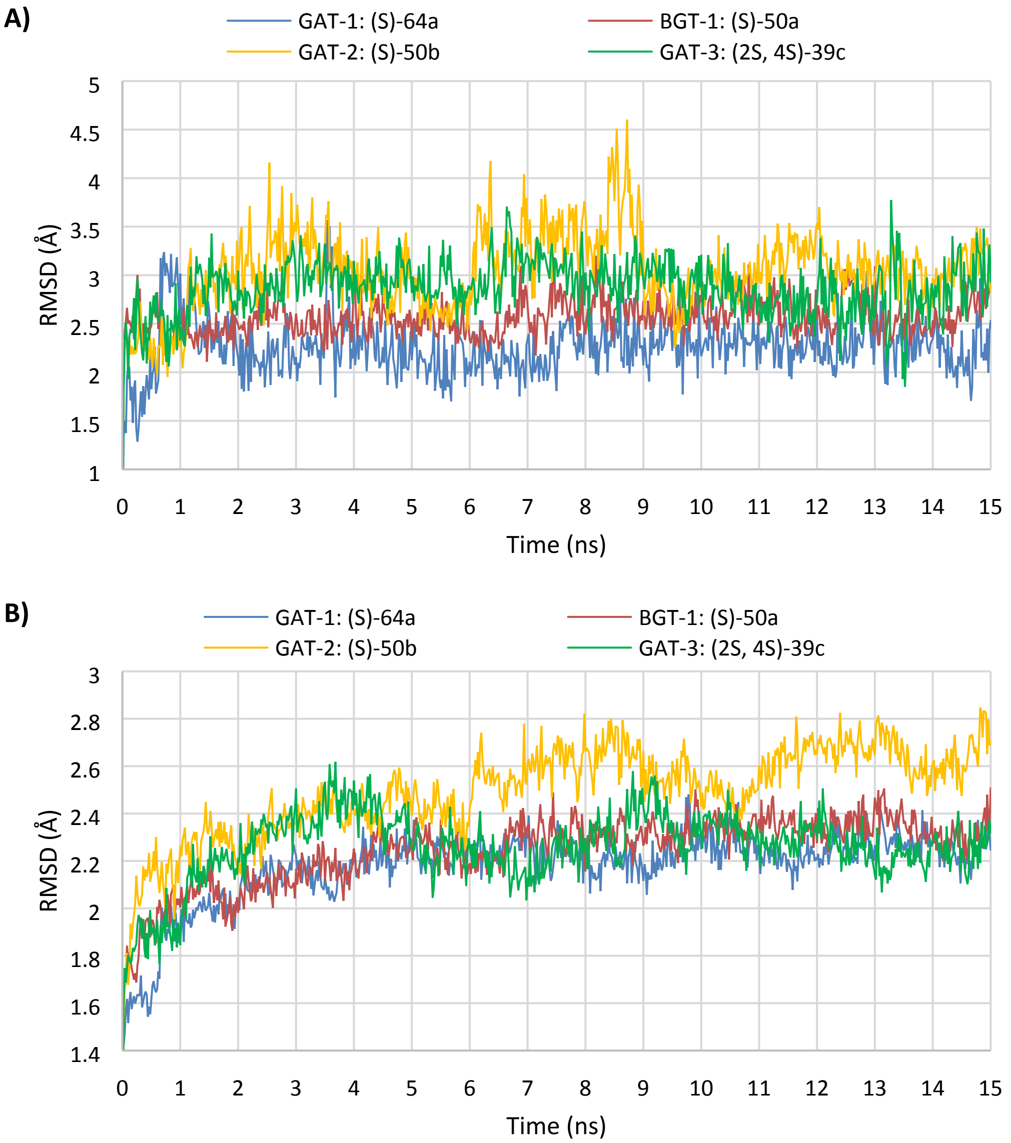

Figure S1. RMSD changes over the course of the molecular dynamics simulations for the compounds (Panel A) and protein residues within 8Å of ligand (Panel B).

2. METHODS

2.1 Chemistry

2.1.1 Synthesis of amine intermediates A-F

2.1.1.1 Synthesis of amine A

Step 1: tert-Butyl 4-[hydroxybis(3-methylthiophen-2-yl)methyl]piperidine-1-carboxylate (**16**)

Under Ar, 3-bromo-2-methylthiophene (5 g, 27 mmol) was added to a suspension of magnesium (0.69 g, 27 mmol) and several iodine crystals in dry THF (25 mL). The mixture stirred for 3 h, and then 1-*tert*-butyl 4-ethyl piperidine-1,4-dicarboxylate (3.14 g, 12 mmol) in dry THF (5 mL) was added dropwise to the above mixture at 0°C. Then, the reaction stirred overnight at rt, was quenched with a 16% NH_4Cl water solution, and extracted with EtOAc (2 × 25 mL). The organic layer was washed with saturated NaCl aqueous solution and dried over anhydrous Na_2SO_4. The crude product was purified via crystallization with PE to yield **16** (2.12 g, 43%, R_f = 0.50 (PE/EtOAc 7:3; S_1)) as a white powdery crystal. Formula $C_{21}H_{29}NO_3S_2$, FW 407.59. ^{1}H NMR (300 MHz, chloroform-d) δ ppm 7.11 (d, *J* = 5.27 Hz, 2H), 6.74 (d, *J* = 5.27 Hz, 2H), 4.07 - 4.27 (m, 2H), 2.74 (t, *J* = 12.60 Hz, 2H), 2.41 - 2.53 (m, 1H), 2.38 (s, 1H), 1.94 (s, 8H), 1.36 - 1.48 (m, 11H).

Step 2: 4-[Bis(3-methylthiophen-2-yl)methylidene]piperidine (**17**)

para-Toluenesulfonic acid (0.79 g, 4.6 mmol) was added to a solution of alcohol **16** (1.5 g, 3.83 mmol) in toluene (25 mL). The reaction mixture stirred at reflux for 6 h. Then, the reaction was quenched with 1 M NaOH and extracted with DCM (2 × 15 mL). The organic layer was washed with saturated NaCl aqueous solution and dried over anhydrous Na_2SO_4. The crude product was purified by column chromatography over silica gel (PE/EtOAc = 1:1) to yield **17** (1.03 g, 96%, R_f = 0.21 (PE/EtOAc = 1:1; S_2) as a yellow oil. Formula $C_{16}H_{19}NS_2$, FW 289.46. ^{1}H NMR (300 MHz, chloroform-d) δ ppm 7.12 (d, *J* = 5.27 Hz, 2H), 6.78 (d, *J* = 4.69 Hz, 2H), 2.88 - 2.96 (m, 4H), 2.26 - 2.33 (m, 4H), 2.11 - 2.17 (m, 6H), 1.91 (br. s, 1H)

2.1.1.2 Synthesis of amine B: 3-(5*H*-Dibenzo[a,d][7]annulen-5-ylidene)-*N*-methylpropan-1-amine (**20**)

A solution of **18** (30 mmol, 9.34 g) and 41 mL of methylamine (33 wt. % in absolute ethanol) was stirred for 48 h at room temperature. Then, the mixture was extracted with DCM (3 × 50 mL). The combined organic extracts were washed with H_2O, dried ($MgSO_4$) and concentrated under reduced pressure. The crude product was purified by column chromatography over silica gel (DCM/Ace = 9:1) to yield **20** (6.5 g, 83%, R_f = 0.81 (*n*-hexane:EtOH:TEA 7:2:1, S_3)) as a yellow oil. ^{1}H NMR (chloroform-d): δ ppm 7.21 -

7.37 (m, 8H, Ar), 6.86 (d, *J*=1.03 Hz, 2H ArCHCHAr), 5.53 (dd, *J*=8,03, 6.91 Hz, 1H, C=CH), 2.61 (td, *J*=6.86, 4.49 Hz, 2H, -CH_2-NH-), 2.30 (m, 5H, H_3C-NH-CH_2-CH_2-)

2.1.1.3 Synthesis of amine C: 3-(10,11-Dihydro-5*H*-dibenzo[a,d][7]annulen-5-ylidene)-*N*-methylpropan-1-amin (**21**)

A solution of **19** (25.6 mmol, 8.02 g) and 35 mL of methylamine (33 wt. % in absolute ethanol) was stirred for 48 h at room temperature. Then, the mixture was extracted with DCM (3 × 40 mL). The combined organic extracts were washed with H_2O, dried ($MgSO_4$) and concentrated under reduced pressure. The crude product was purified by column chromatography over silica gel (DCM/Ace = 7:3) to yield **21** (4.18 g, 62%, R_f = 0.52 (DCM/MeOH/NH_3 9.5:0.5:0.1, S_4) as a yellow oil. ^{1}H NMR (chloroform-d): δ ppm 7.26 - 7.31 (m, 1H, Ar) 7.08 - 7.23 (m, 6H, Ar), 7.01 - 7.07 (m, 1H, Ar), 5.85 (t, *J*=7.44 Hz, 1H, C=*CH*) 3,47 (s, 4H, ArCH_2CH_2Ar), 2.26 - 2.63 - 2.71 (m, 2H, NHCH_2), 2.37 (s, 3H, Me), 2.36 (m, 2H, =CH-CH_2).

2.1.1.4 Synthesis of amine D: 4-(9*H*-Fluoren-9-ylidene)piperidine (**25**)

Under Ar, zinc (1.34 g, 20.52 mmol) was added to a mixture of tert-butyl 4-oxopiperidine-1-carboxylate (1.0 g, 5.02 mmol) and 9*H*-fluoren-9-one (0.82 g, 4.56 mmol) in dry THF (10 mL). The mixture was cooled to 0°C, and $TiCl_4$ (1.13 mL, 10.26 mmol) was added over 15 min. The mixture was heated to reflux for 1.5 h. Then, the reaction was quenched with 1 M HCl and extracted with toluene (2 × 20 mL). The organic layer was washed with 10% potassium carbonate aqueous solution and dried over anhydrous Na_2SO_4. The crude product was purified by column chromatography over silica gel (PE/EtOAc = 7:3 → 25% NH_3/MeOH/DCM/PE = 9:45:120:18, S_5) to yield **25** (290 mg, 26%, R_f = 0.15 (S_1)) as a yellow oil. Formula $C_{18}H_{17}N$, FW 247.34. ^{1}H NMR (300 MHz, chloroform-d) δ ppm 7.86 - 7.92 (m, 2H), 7.74 - 7.81 (m, 2H), 7.23 - 7.36 (m, 4H), 3.11 - 3.23 (m, 8H), 1.71 (s, 1H).

2.1.1.5 Synthesis of amine E:

Under Ar, zinc (2.50 g, 38.57 mmol) was added to a mixture of tert-butyl 4-oxopiperidine-1-carboxylate (1.88 g, 9.43 mmol) and 10,11-dihydro-5*H*-dibenzo[a,d][7]annulen-5-one (1.78 g, 8.57 mmol) in dry THF (20 mL). The mixture was cooled to 0°C, and $TiCl_4$ (2.11 mL, 19.26 mmol) was added over 15 min. The mixture was heated to reflux for 1.5 h. Then, the reaction was quenched with 1 M HCl and extracted with toluene (2 × 20 mL). The organic layer was washed with 10% potassium carbonate aqueous solution and dried over anhydrous Na_2SO_4. The crude product was purified by column chromatography over silica gel (PE/EtOAc = 7:3 → 25% NH_3/MeOH/DCM/PE = 640:140:100:25, S_{13}) to yield **26** (450 mg, 20%, R_f =

0.68 (S_{13})) as a yellow oil. Formula $C_{20}H_{21}N$, FW 275.39. ^{1}H NMR and consistent with literature data ^{1}H NMR (300 MHz, chloroform-d) δ ppm 7.20 - 6.97 (m, 8 H), 3.47 - 3.34 (m, 2 H), 3.09 - 2.98 (m, 2 H), 2.97 - 2.90 (m, 1 H), 2.89 - 2.70 (m, 4 H), 2.43 - 2.34 (m, 4 H).

2.1.1.6 Synthesis of amine F: tert-Butyl (2-((1,3-dioxoisoindolin-2-yl)oxy)ethyl)(methyl)carbamate (**29**)

A solution of 2-hydroxyisoindoline-1,3-dione To an ice-cold solution of tert-butyl (2- hydroxyethyl) methylcarbamate (3.0 g, 17 mmol), *N*- hydroxyphthalimide (2.8 g, 17 mmol) and triphenylphosphine (4.5 g, 17 mmol) in THF (90 mL) was added a solution of diethyl azodicarboxylate (3.0 mL, 19 mmol) in THF (15 mL) dropwise. The stirred reaction mixture was allowed to warm to ambient temperature. After warming to room temperature and concentrating under vacuum, the product was obtained and purified by column chromatography over silica gel (DCM/Ace 9:1, S_6) to yield **29** (100%, R_f = 0.38 (S_7)) as a yellow oil. ^{1}H NMR (300 MHz, chloroform-d): δ ppm 7.52 - 7.28 (m, 4H, Ar), 4.27 (d, 2H, *J* = 4.87 Hz, $OC\mathit{H_2}CH_2NCH_3$), 3.52 (d, 2H, *J* = 5.39 Hz, $OCH_2C\mathit{H_2}NCH_3$), 2.81 (s, 3H, NCH_3) 1.38 – 1.46 (m, 9H, $C(CH_3)_3$).

2.1.1.7 tert-Butyl (2-(aminooxy)ethyl)(methyl)carbamate (**30**)

To a solution of tert-butyl (2-((1,3-dioxoisoindolin-2-yl)oxy)ethyl)(methyl)carbamate (**29**) (3.3 mmol, 1.1 g) in 15 mL of ethanol was added hydrazine. The reaction was monitored by TLC and quenched after the substrate was consumed. The slurry was extracted with diethyl ether (3 × 40 mL). The combined organic extracts were washed with H_2O and brine, dried ($MgSO_4$) and concentrated under reduced pressure. The crude product was used directly in the next step. ^{1}H NMR (300 MHz, chloroform-d): δ ppm 3.68 (d, 2H, *J* = 4.62 Hz, $OC\mathit{H_2}CH_2NCH_3$), 3.38 – 3.30 (m, 2H, $OCH_2C\mathit{H_2}NCH_3$), 2.87 (s, 3H, NCH_3), 1.41 (s, 9H, $C(CH_3)_3$). *NH_2 protons were not detected.*

2.1.1.8 tert-Butyl (2-(((diphenylmethylene)amino)oxy)ethyl)(methyl)carbamate (**31**)

To a solution of crude product **30** (1.15 mmol, 219 mg) and benzophenone (1.4 mmol, 255 mg) in MeOH (3 mL), NaOH (240 mg) was added. The reaction mixture stirred for 30 h at 65°C. The cooled solution was poured into H_2O (10 mL) and concentrated under reduced pressure. The obtained crude product was purified using column chromatography to yield **31** (50%, R_f = 0.23 (S_7)) as a yellow oil. ^{1}H NMR (300 MHz, chloroform-d): δ ppm 7.52 - 7.28 (m, 10H, Ar), 4.27 (d, *J* =4.87 Hz, 2H, $OC\mathit{H_2}CH_2NCH_3$) 3.52 (d, *J* = 5.39 Hz, 2H, $OCH_2C\mathit{H_2}NCH_3$) 2.81 (br. s, 3H, NCH_3) 1.38 – 1.46 (m, 9 H, $C(CH_3)_3$).

2.1.1.9 Amine F: Diphenylmethanone O-(2-(methylamino)ethyl) oxime (**32**)

In a dry 25 mL round bottom flask equipped with a stirring bar, *tert*-butyl (2-(((diphenylmethylene)amino)oxy)ethyl)(methyl)carbamate (50 mg) was dissolved in DCM (5 mL), and then 1 mL of trifluoroacetic acid (TFA) was added to the solution. The reaction was monitored via TLC. Upon complete conversion of the *N*-Boc-protected amine, deionized water (5 mL) was slowly added to the flask followed by quenching with saturated aqueous $NaHCO_3$ and extraction with EtOAc (3 × 10 mL). The combined organic extracts were washed with H_2O, dried ($MgSO_4$) and concentrated under reduced pressure. The obtained crude product was purified using column chromatography (DCM/MeOH, 95:5) to yield **32** (98%, R_f = 0.23 (S_8)) as a yellow oil. ^1H NMR (300 MHz, chloroform-d): δ ppm 7.56 – 7.21 (m, 10H, Ar), 4.35 – 4.25 (m, 2H, OCH_2CH$_2$NCH$_3$), 2.95 – 2.84 (m, 2H, OCH$_2$CH_2NCH$_3$), 2.43 (s, 3H, CH_3), 1.60 (s, 1H, N*H*).

3. ^1H AND ^{13}C NMR SPECTRA OF SYNTHESIZED COMPOUNDS

^1H NMR spectrum of 2-{4-[Bis(3-methylthiophen-2-yl)methylidene]piperidin-1-yl}-*N*-[(2-chlorophenyl)methyl]-4-hydroxypentanamide (2*RS*,4*SR*)-**37b**

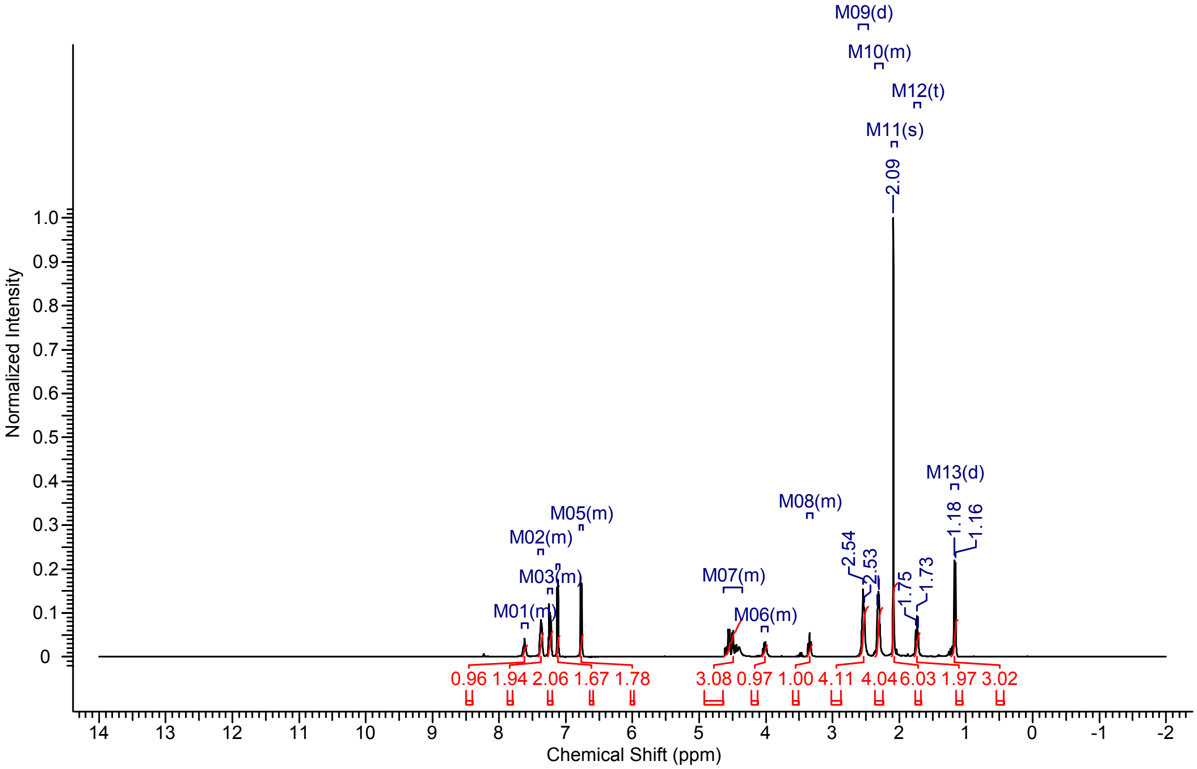

^{13}C NMR spectrum of 2-{4-[Bis(3-methylthiophen-2-yl)methylidene]piperidin-1-yl}-*N*-[(2-chlorophenyl)methyl]-4-hydroxypentanamide (2*RS*,4*SR*)-**37b**

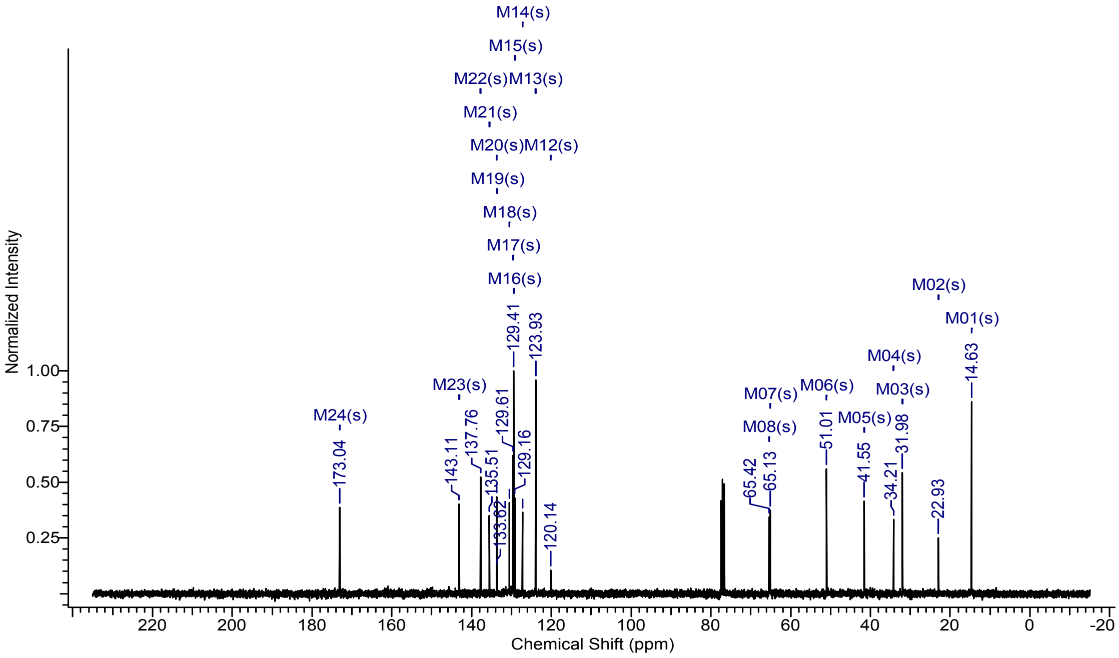

^{1}H NMR spectrum of 2-{4-[Bis(3-methylthiophen-2-yl)methylidene]piperidin-1-yl}-*N*-[(4-fluorophenyl)methyl]-4-hydroxypentanamide (2*RS*,4*SR*)-**37c**

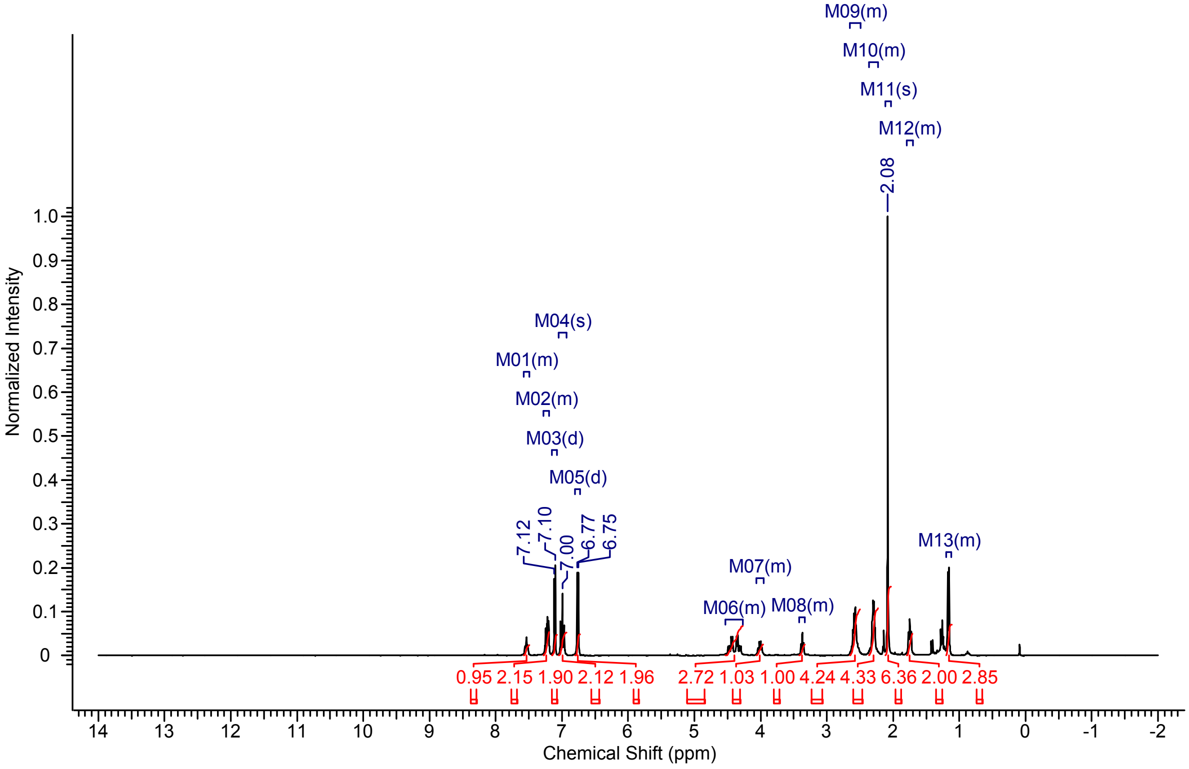

^{13}C NMR spectrum of 2-{4-[Bis(3-methylthiophen-2-yl)methylidene]piperidin-1-yl}-*N*-[(4-fluorophenyl)methyl]-4-hydroxypentanamide (2*RS*,4*SR*)-**37c**

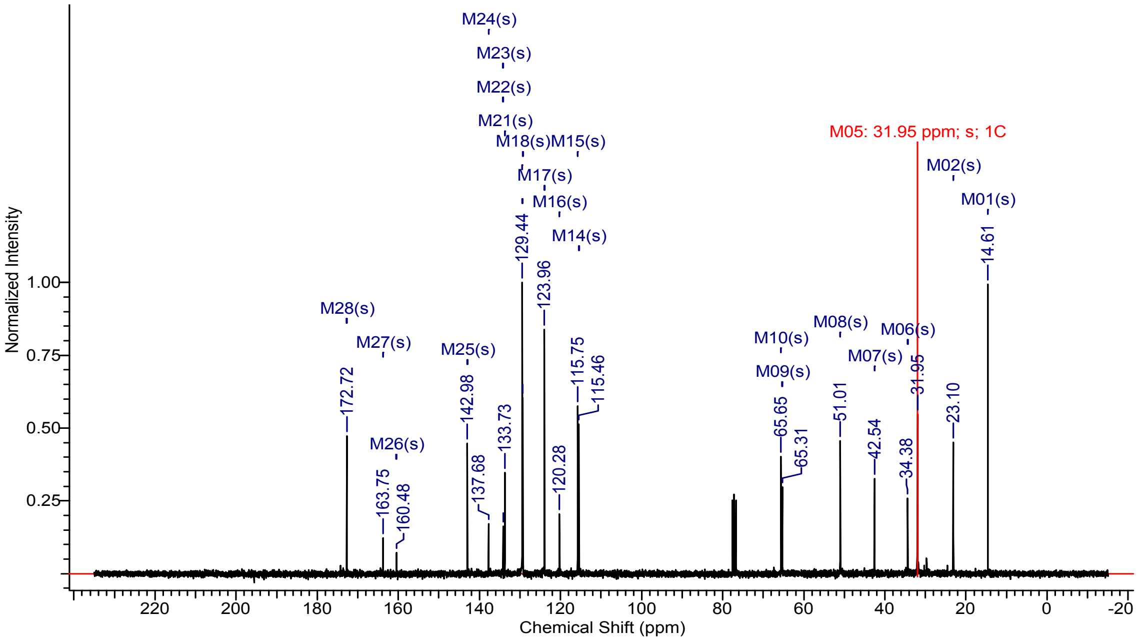

^{1}H NMR spectrum of *N*-(4-Chlorobenzyl)-2-((3-(10,11-dihydro-5*H*-dibenzo[a,d][7]annulen-5-ylidene)propyl)(methyl)amino)-4-hydroxypentanamide (2*RS*,4*SR*)-**39b**

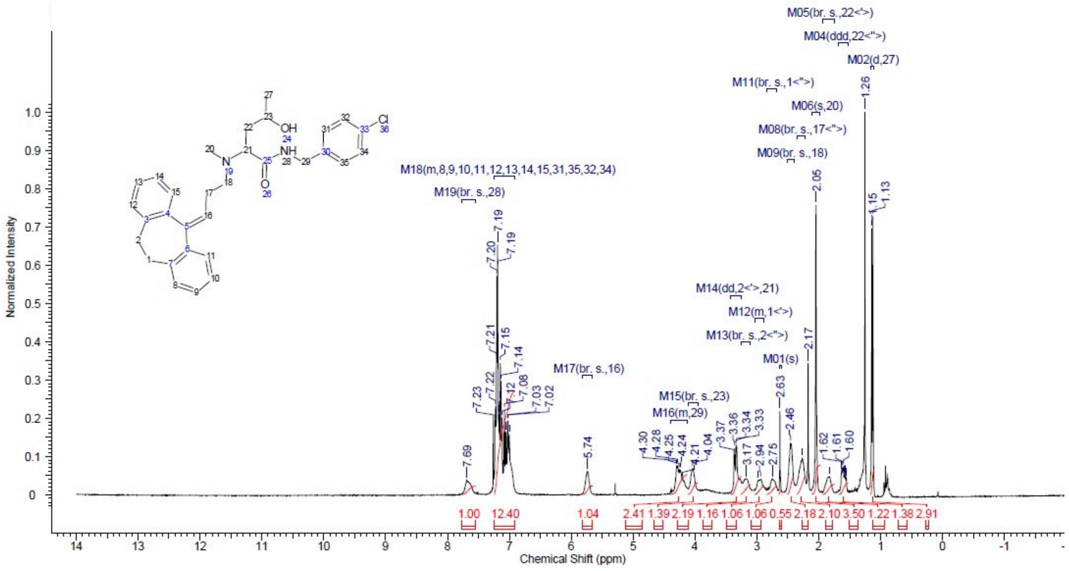

^{13}C NMR spectrum of *N*-(4-Chlorobenzyl)-2-((3-(10,11-dihydro-5*H*-dibenzo[a,d][7]annulen-5-ylidene)propyl)(methyl)amino)-4-hydroxypentanamide (2*RS*,4*SR*)-**39b**

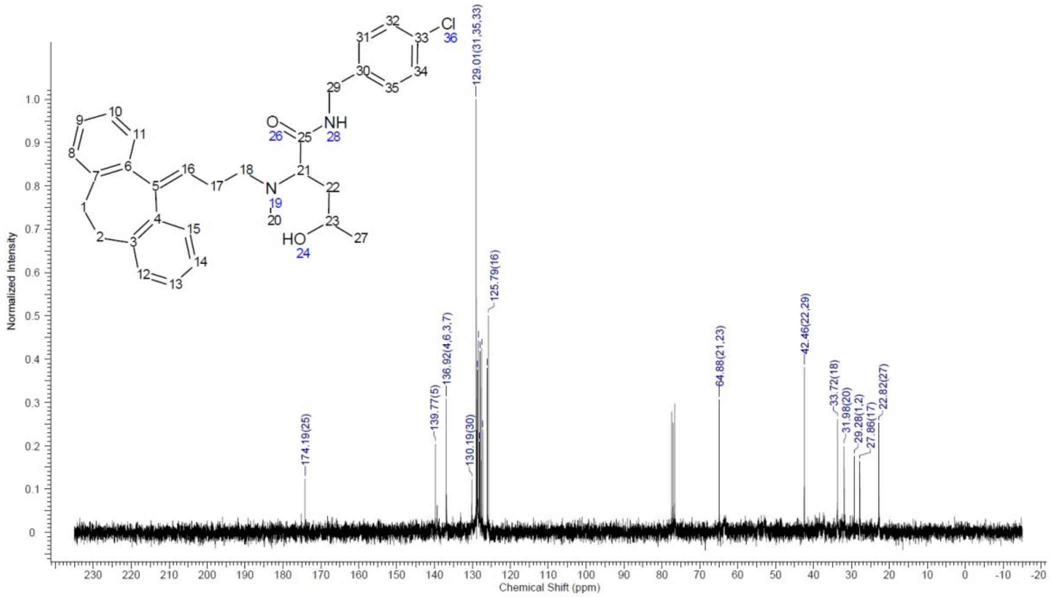

^{1}H NMR spectrum of 2-((3-(10,11-Dihydro-5*H*-dibenzo[a,d][7]annulen-5-ylidene)propyl)(methyl)amino)-4-hydroxy-*N*-(4-methylbenzyl)pentanamide (2*RS*,4*RS*)-**39c**

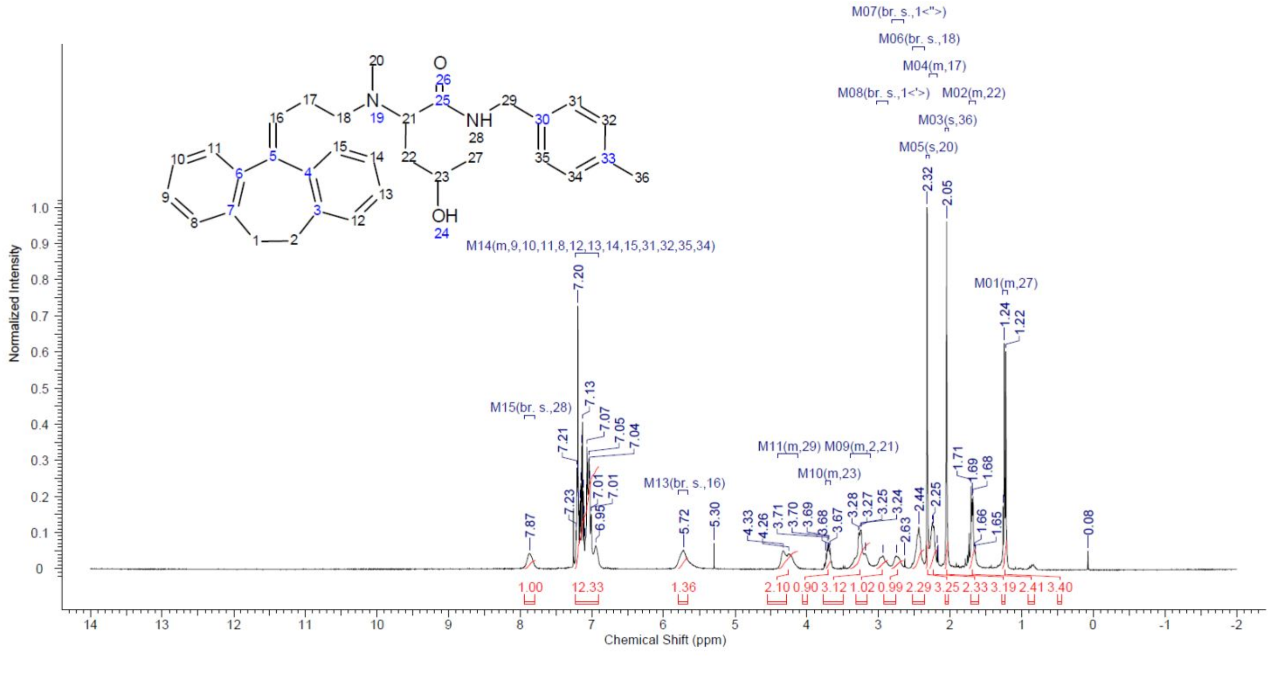

^{1}H NMR spectrum of 2-((3-(10,11-Dihydro-5*H*-dibenzo[a,d][7]annulen-5-ylidene)propyl)(methyl)amino)-4-hydroxy-*N*-(4-methylbenzyl)pentanamide (2*RS*,4*SR*)-**39c**

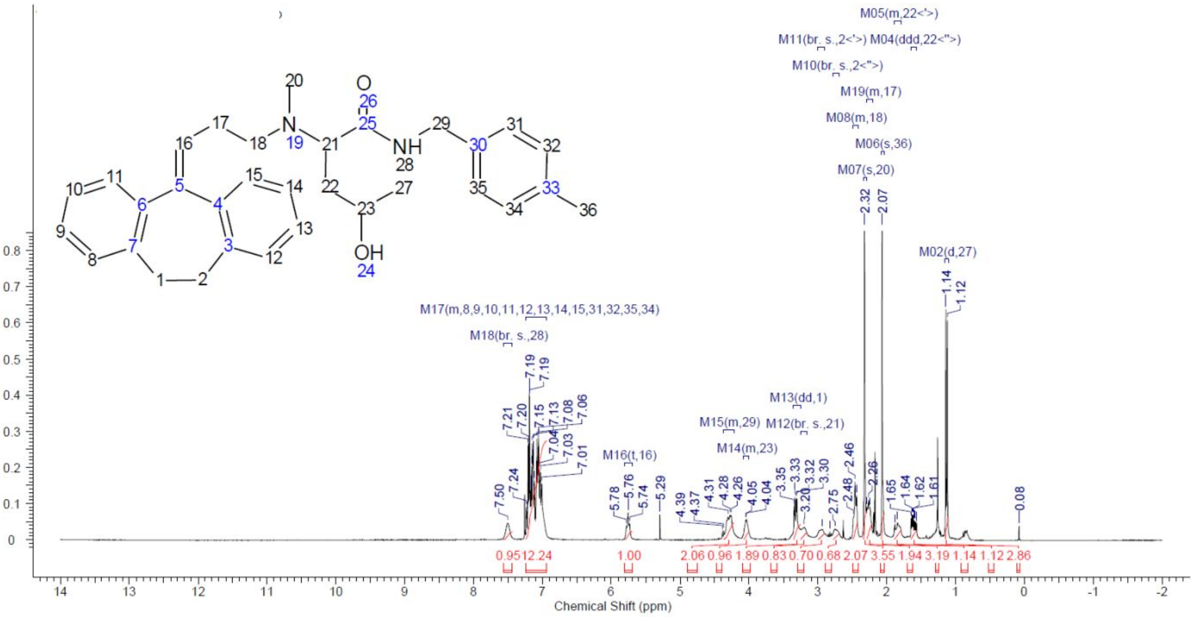

^{13}C NMR spectrum of 2-((3-(10,11-Dihydro-5*H*-dibenzo[a,d][7]annulen-5-ylidene)propyl)(methyl)-amino)-4-hydroxy-*N*-(4-methylbenzyl)pentanamide (2*RS*,4*SR*)-**39c**

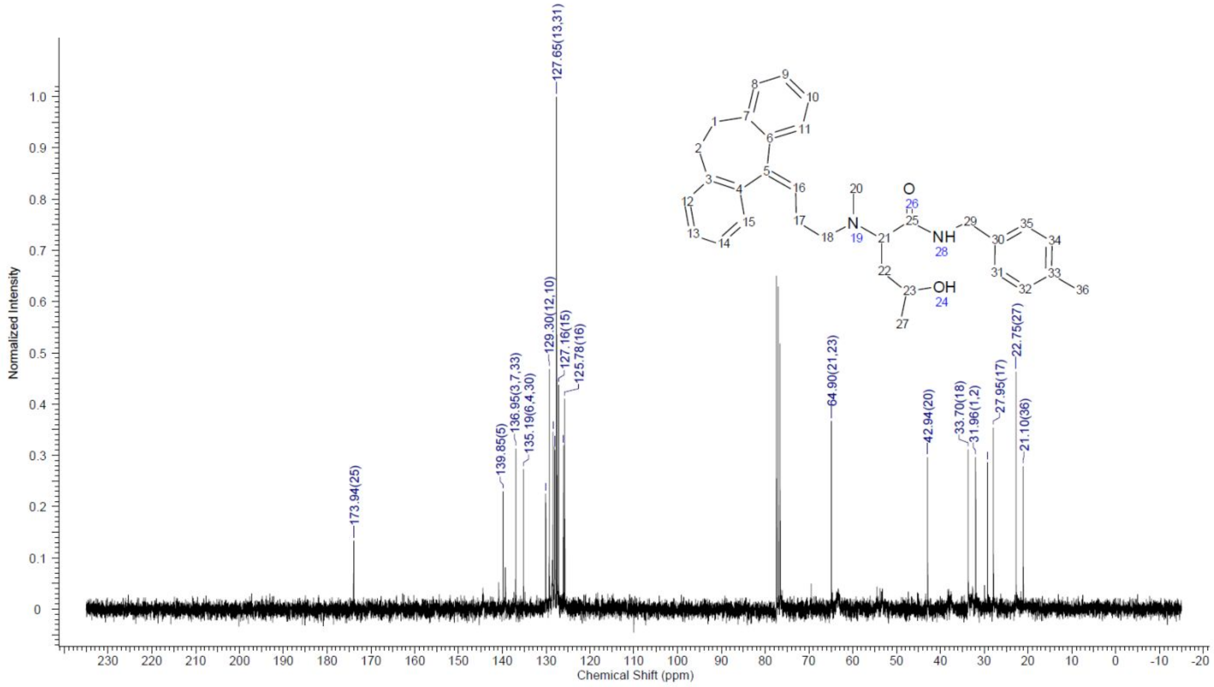

^{1}H NMR spectrum of *N*-benzyl-2-(4-(10,11-dihydro-5H-dibenzo[a,d][7]annulen-5-ylidene)piperidin-1-yl)-4-hydroxypentanamide (2*RS*,4*RS*)-**43a**

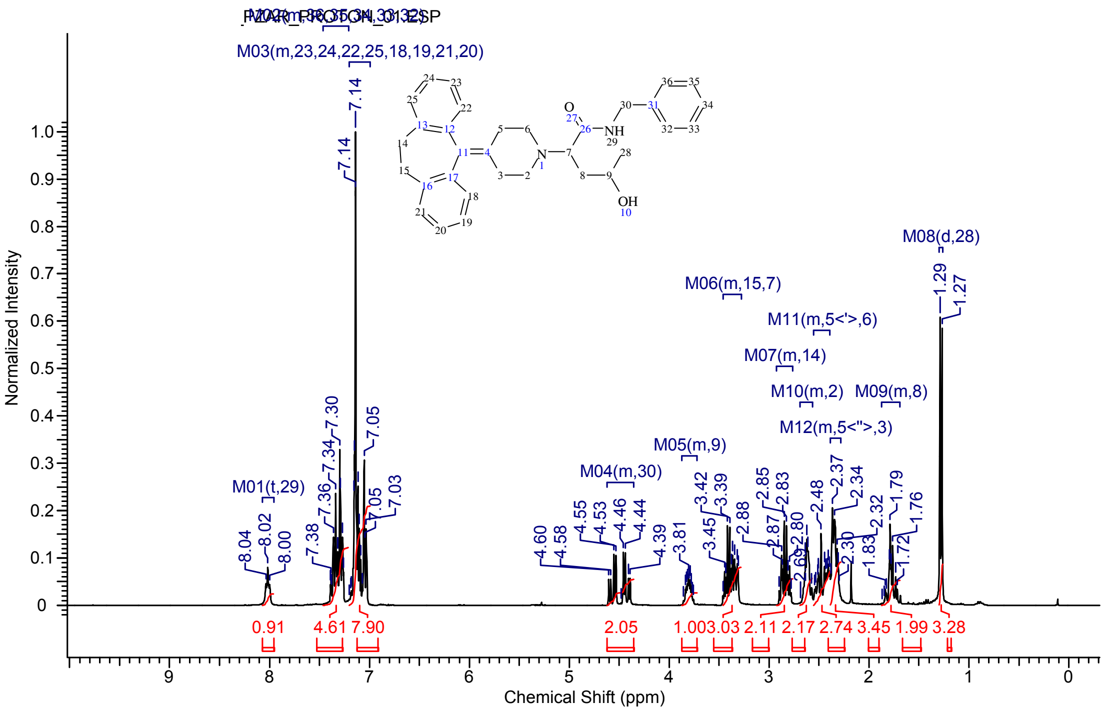

^{13}C NMR spectrum of *N*-benzyl-2-(4-(10,11-dihydro-5H-dibenzo[a,d][7]annulen-5-ylidene)piperidin-1-yl)-4-hydroxypentanamide (2*RS*,4*RS*)-**43a**

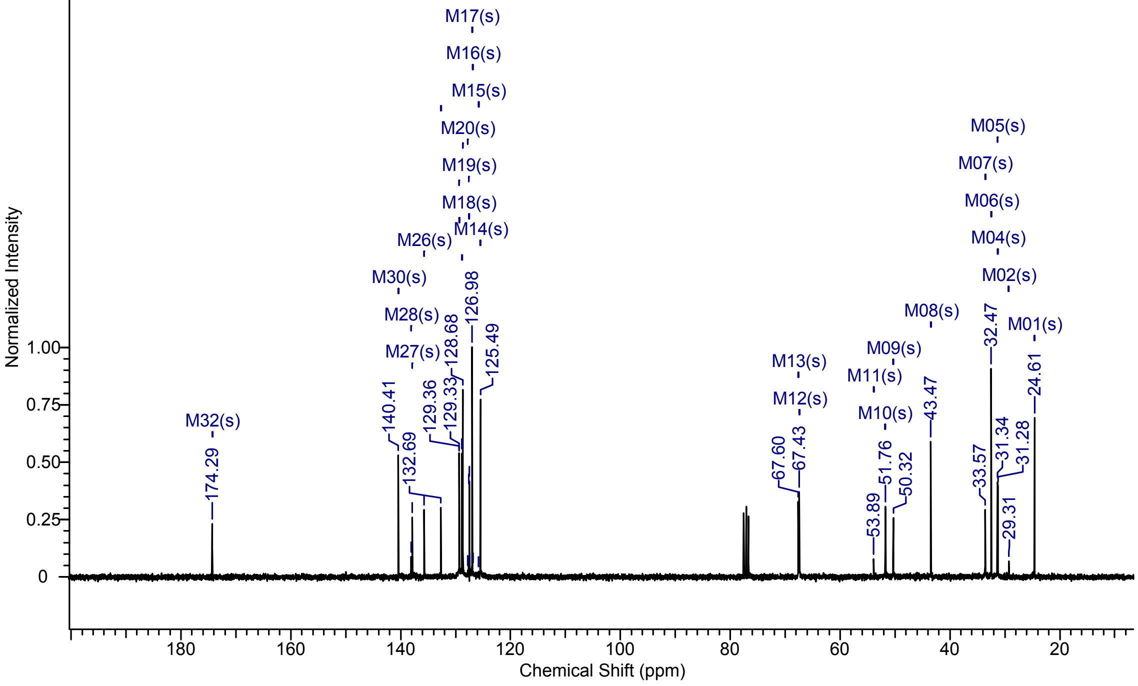

^{1}H NMR spectrum of *N*-benzyl-2-(4-(10,11-dihydro-5H-dibenzo[a,d][7]annulen-5-ylidene)piperidin-1-yl)-4-hydroxypentanamide (2*RS*,4*SR*)-**43a**

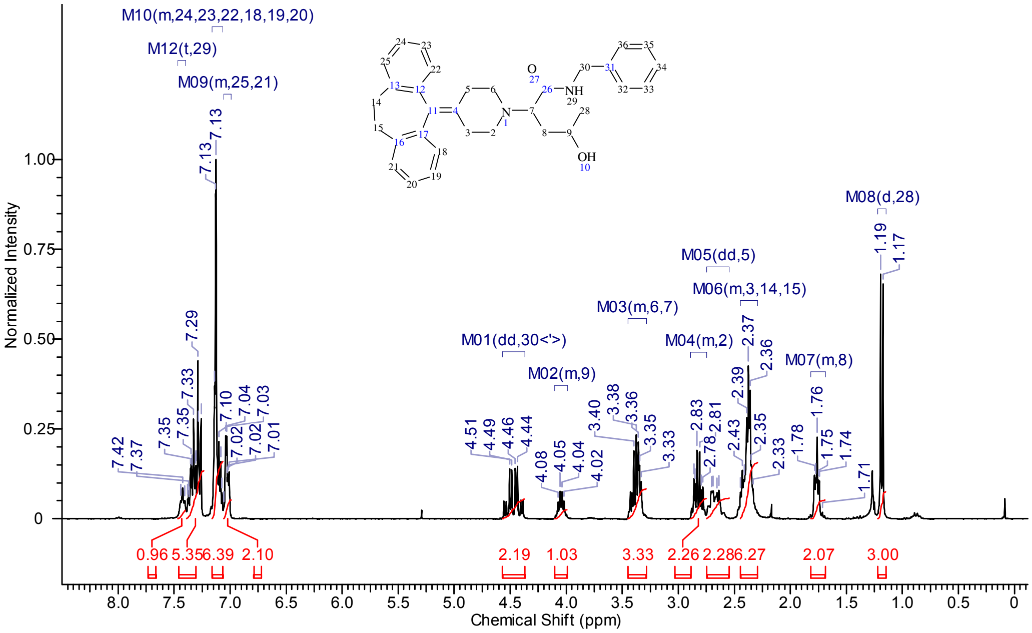

^{13}C NMR spectrum of *N*-benzyl-2-(4-(10,11-dihydro-5H-dibenzo[a,d][7]annulen-5-ylidene)piperidin-1-yl)-4-hydroxypentanamide (2*RS*,4*SR*)-**43a**

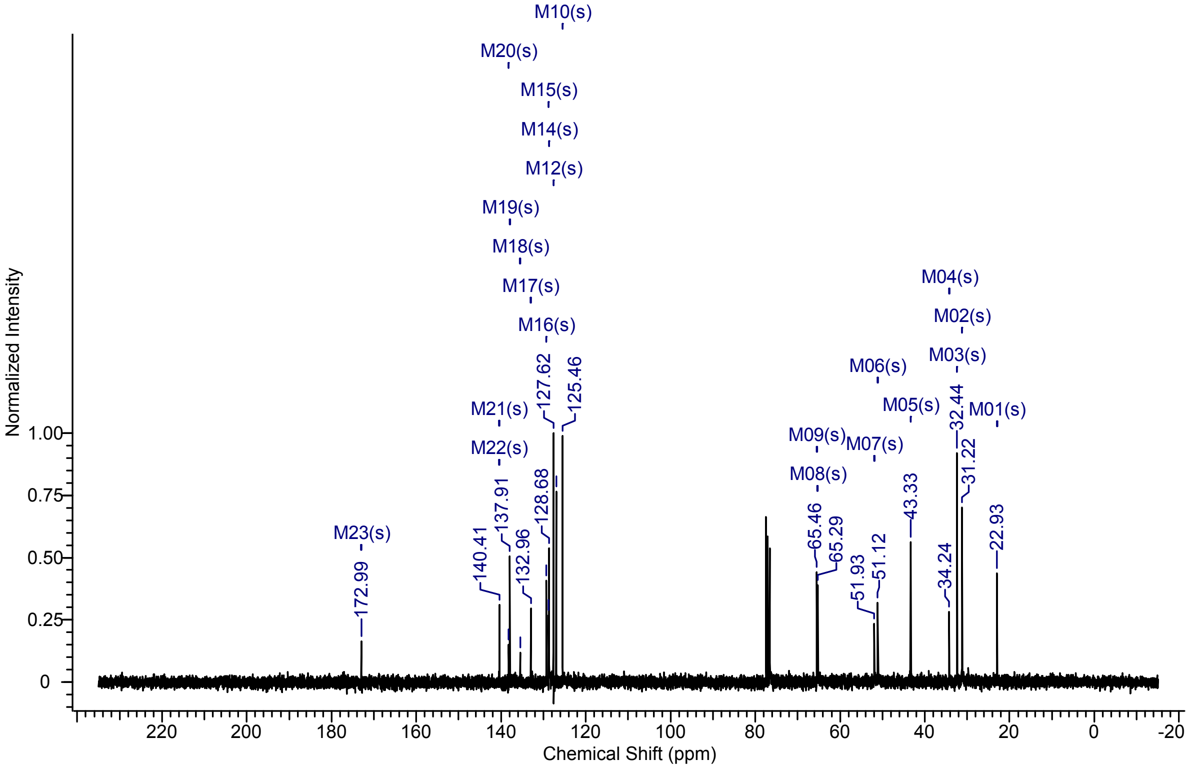

^{1}H NMR spectrum of *N*-Benzyl-2-[(2-{[(diphenylmethylidene)amino]oxy}ethyl)(methyl)amino]-4-hydroxybutanamide (**47a**)

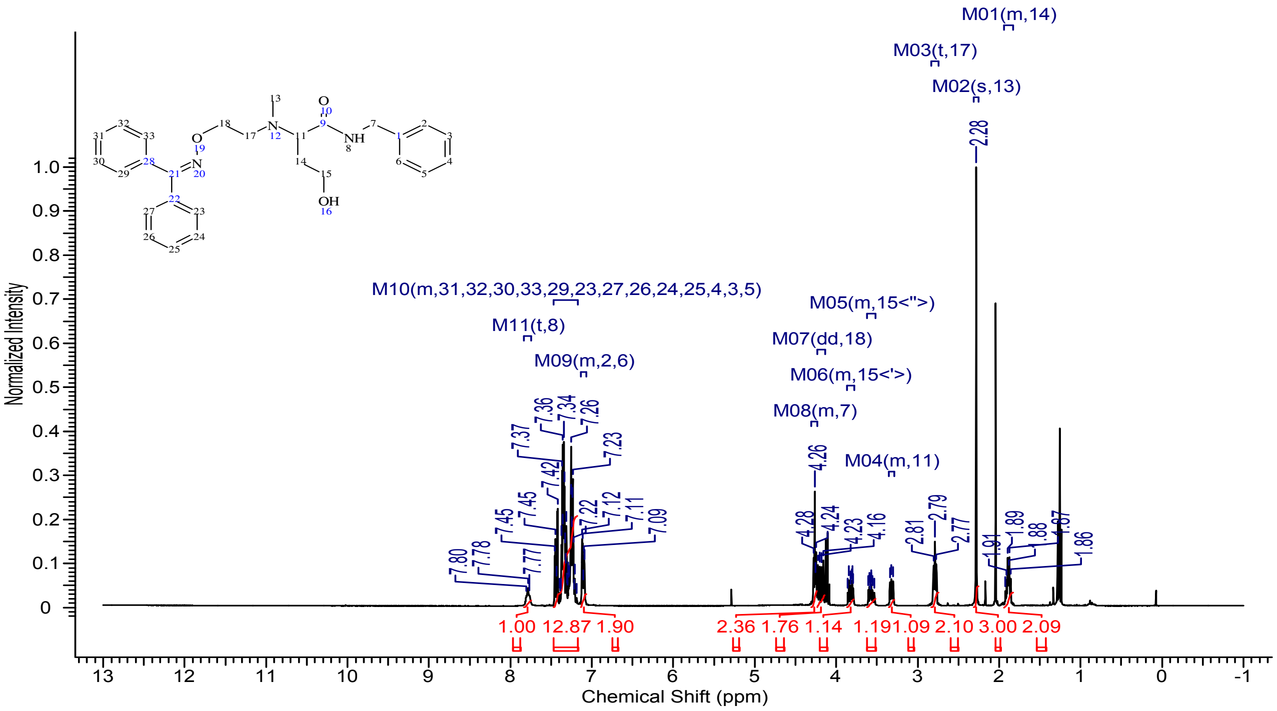

^{13}C NMR spectrum of *N*-Benzyl-2-[(2-{[(diphenylmethylidene)amino]oxy}ethyl)(methyl)amino]-4-hydroxybutanamide (**47a**)

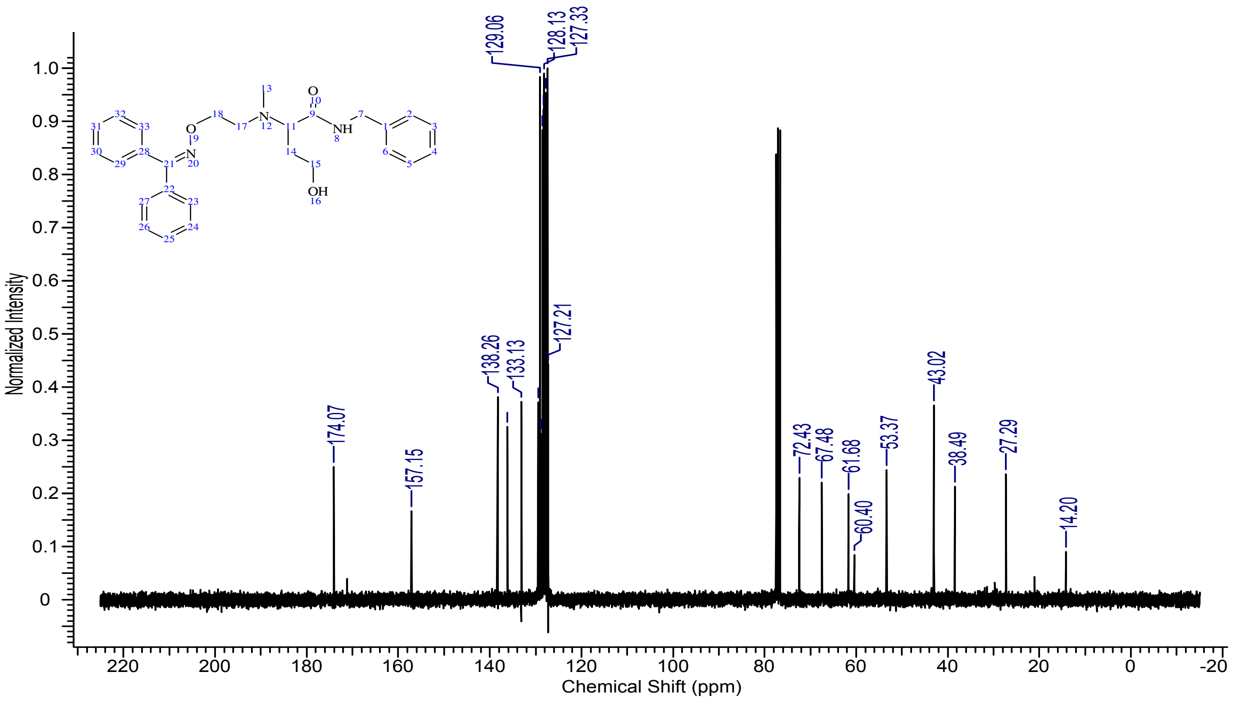

^{1}H NMR spectrum of *N*-[(2-Chlorophenyl)methyl]-2-[(2-{[(diphenylmethylidene)amino]oxy}-ethyl)(methyl)amino]-4-hydroxybutanamide (**47b**)

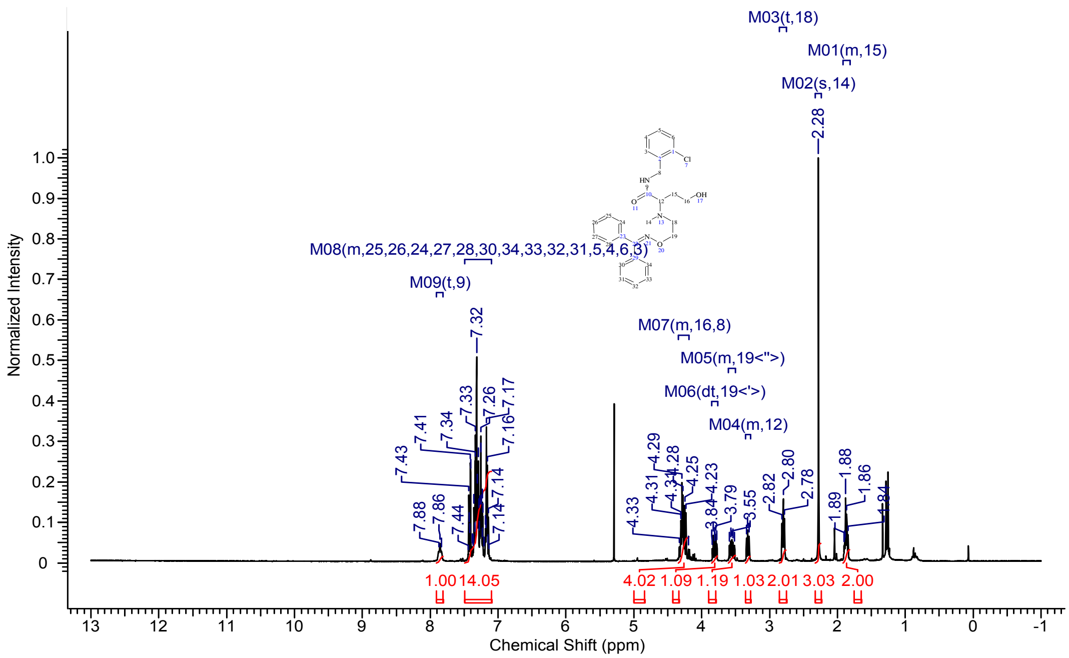

^{13}C NMR spectrum of *N*-[(2-Chlorophenyl)methyl]-2-[(2-{[(diphenylmethylidene)amino]oxy}-ethyl)(methyl)amino]-4-hydroxybutanamide (**47b**)

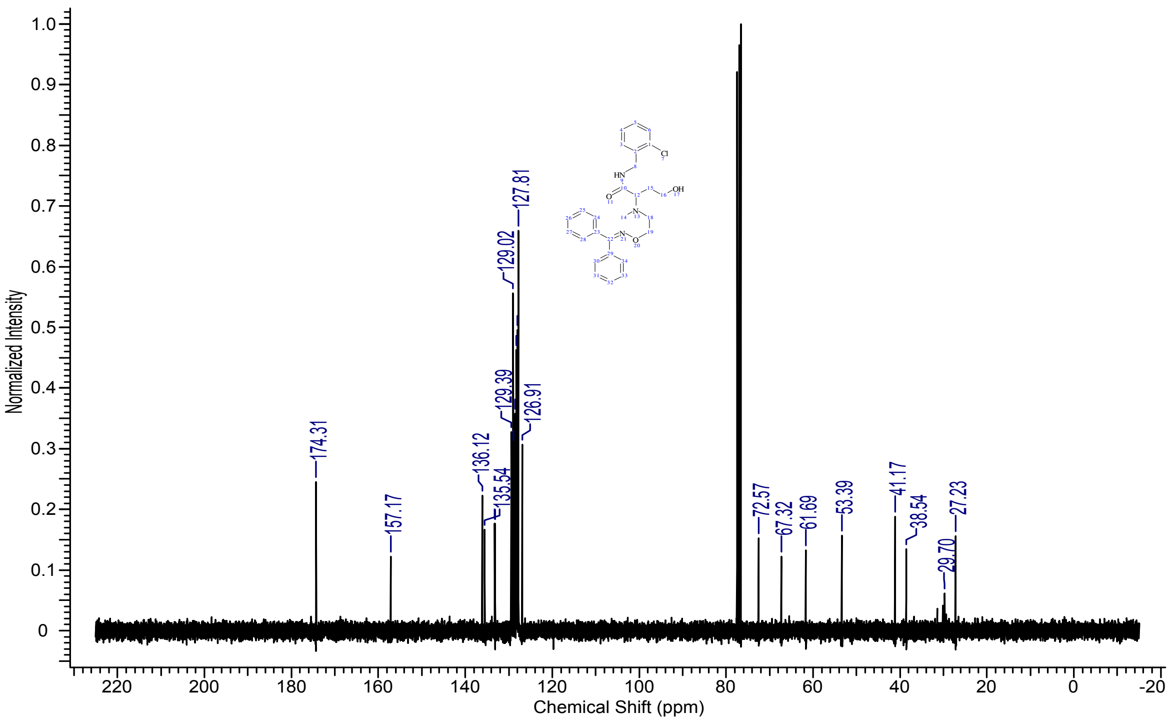

^{1}H NMR spectrum of *N*-[(3,4-Dichlorophenyl)methyl]-2-[(2-{[(diphenylmethylidene)amino]oxy}-ethyl)(methyl)amino]-4-hydroxybutanamide (**47c**)

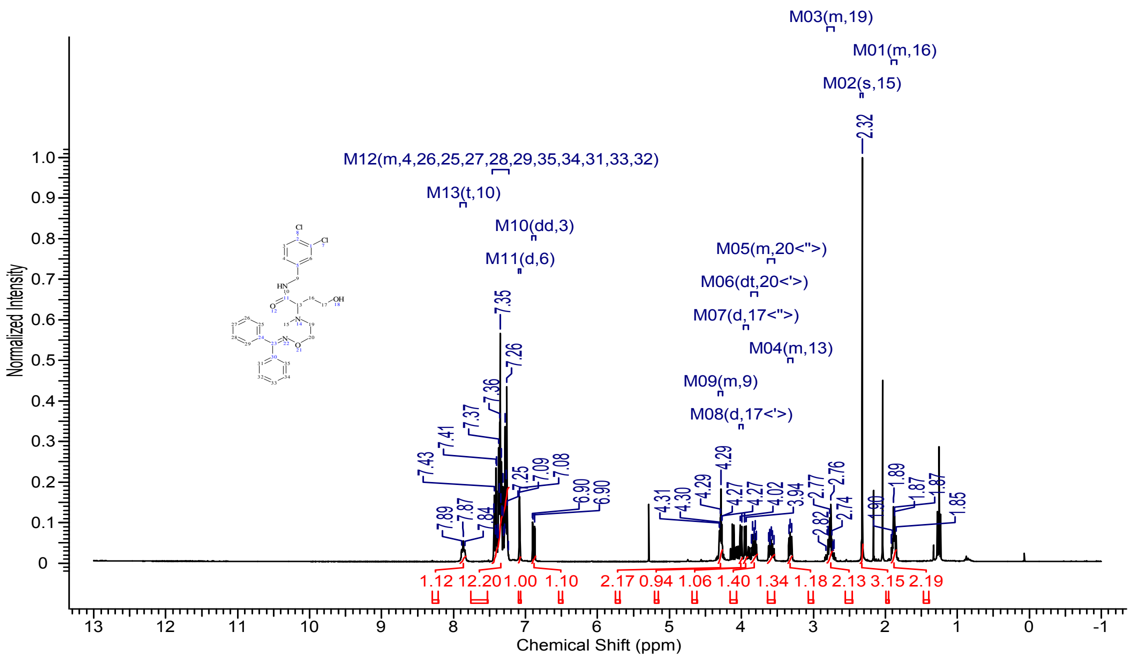

^{13}C NMR spectrum of *N*-[(3,4-Dichlorophenyl)methyl]-2-[(2-{[(diphenylmethylidene)amino]oxy}-ethyl)(methyl)amino]-4-hydroxybutanamide (**47c**)

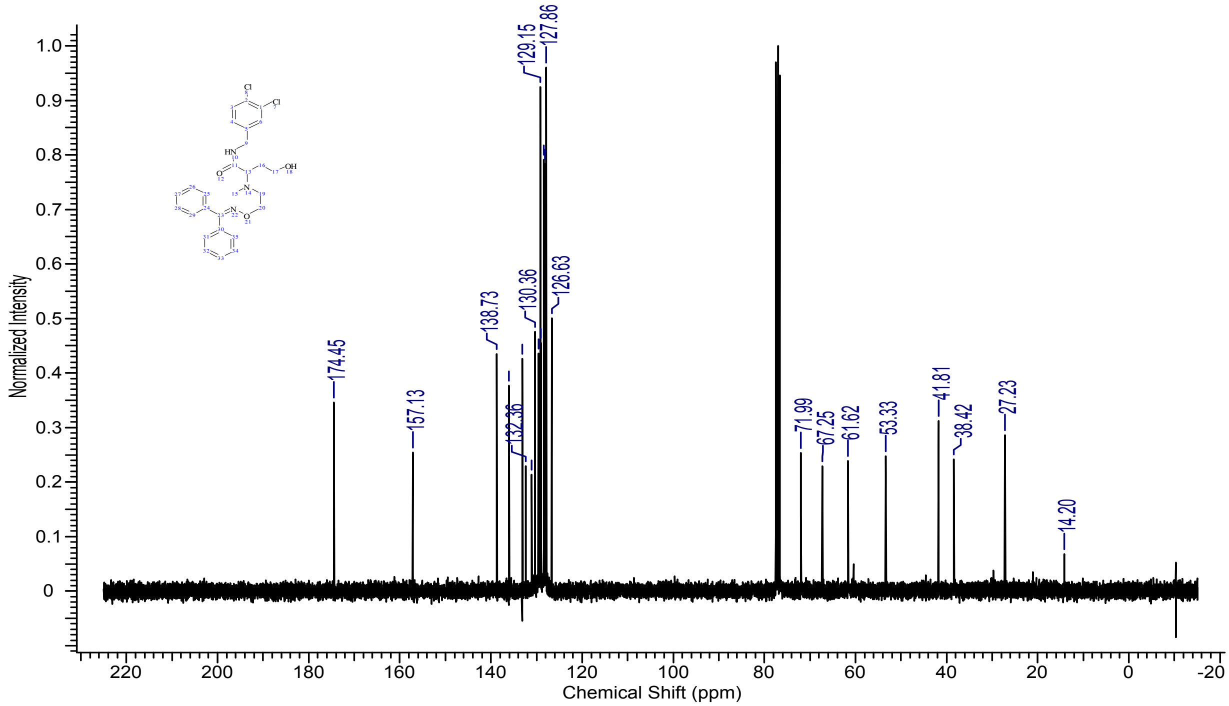

^{1}H NMR spectrum of 4-Amino-*N*-[(2-chlorophenyl)methyl]-2-[(2-{[(diphenylmethylidene)amino]oxy}-ethyl)(methyl)amino]butanamide (**50a**)

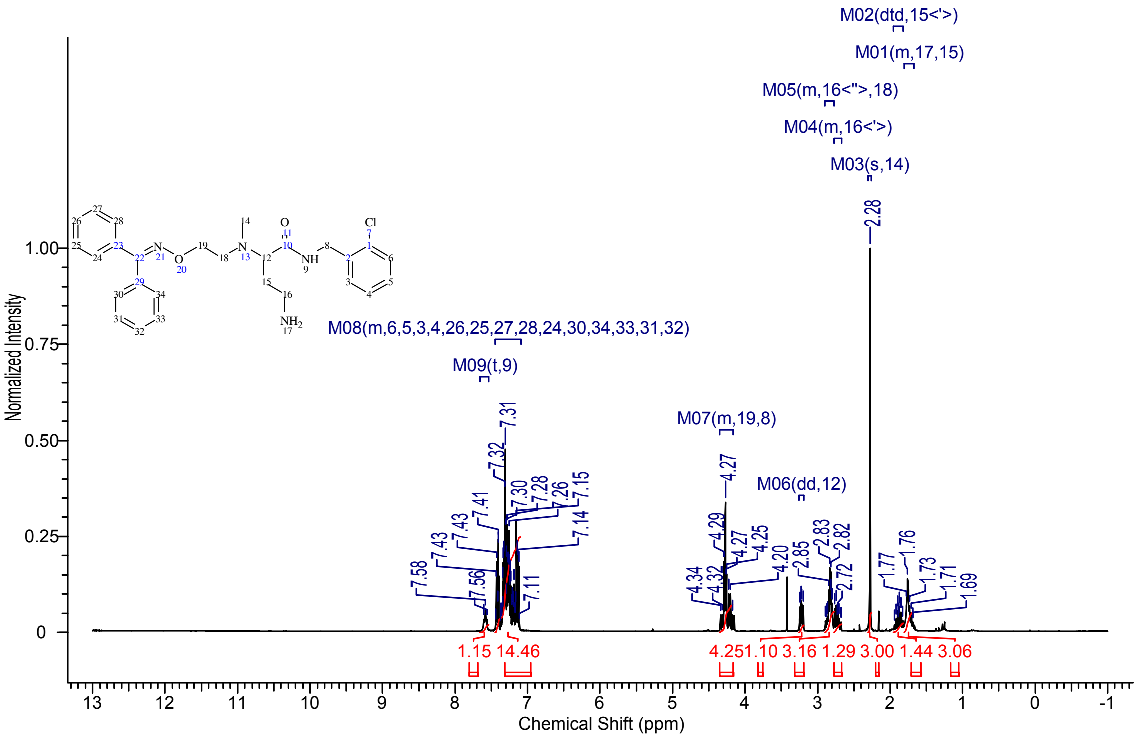

^{13}C NMR spectrum of 4-Amino-*N*-[(2-chlorophenyl)methyl]-2-[(2-{[(diphenylmethylidene)amino]oxy}-ethyl)(methyl)amino]butanamide (**50a**)

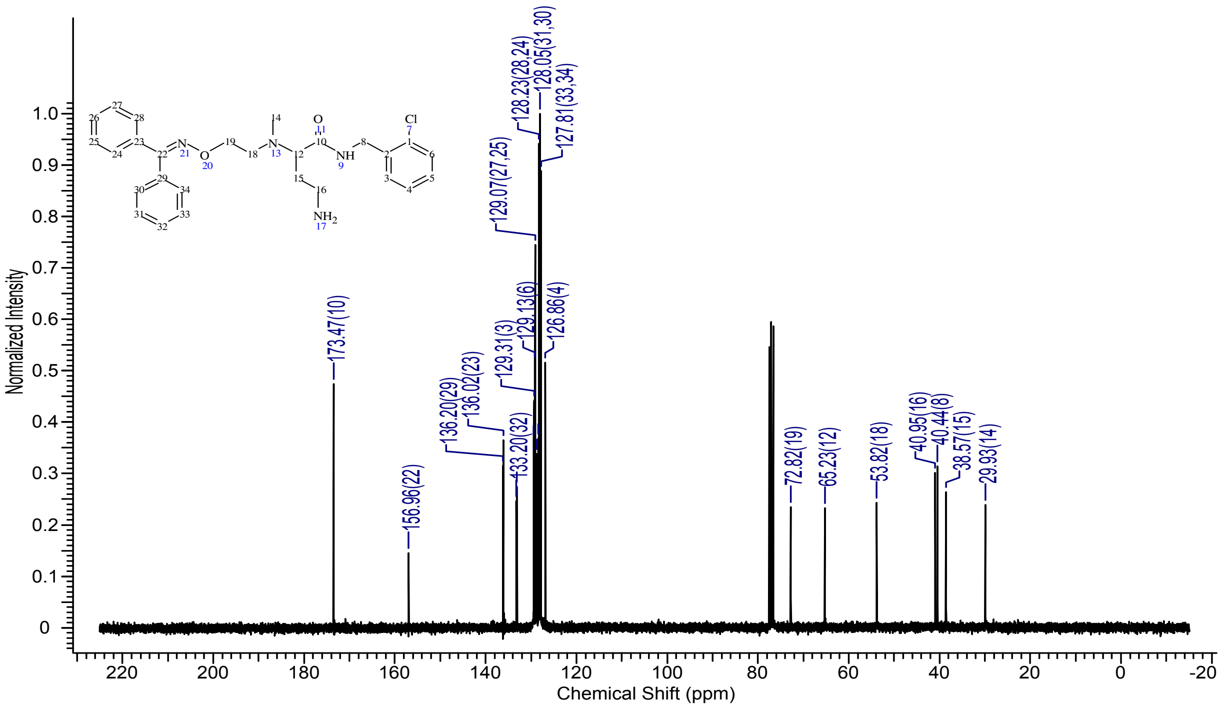

^{1}H NMR spectrum of 4-Amino-*N*-[(3,4-dichlorophenyl)methyl]-2-[(2-{[(diphenylmethylidene)amino]-oxy}ethyl)(methyl)amino] butanamide (**50b**)

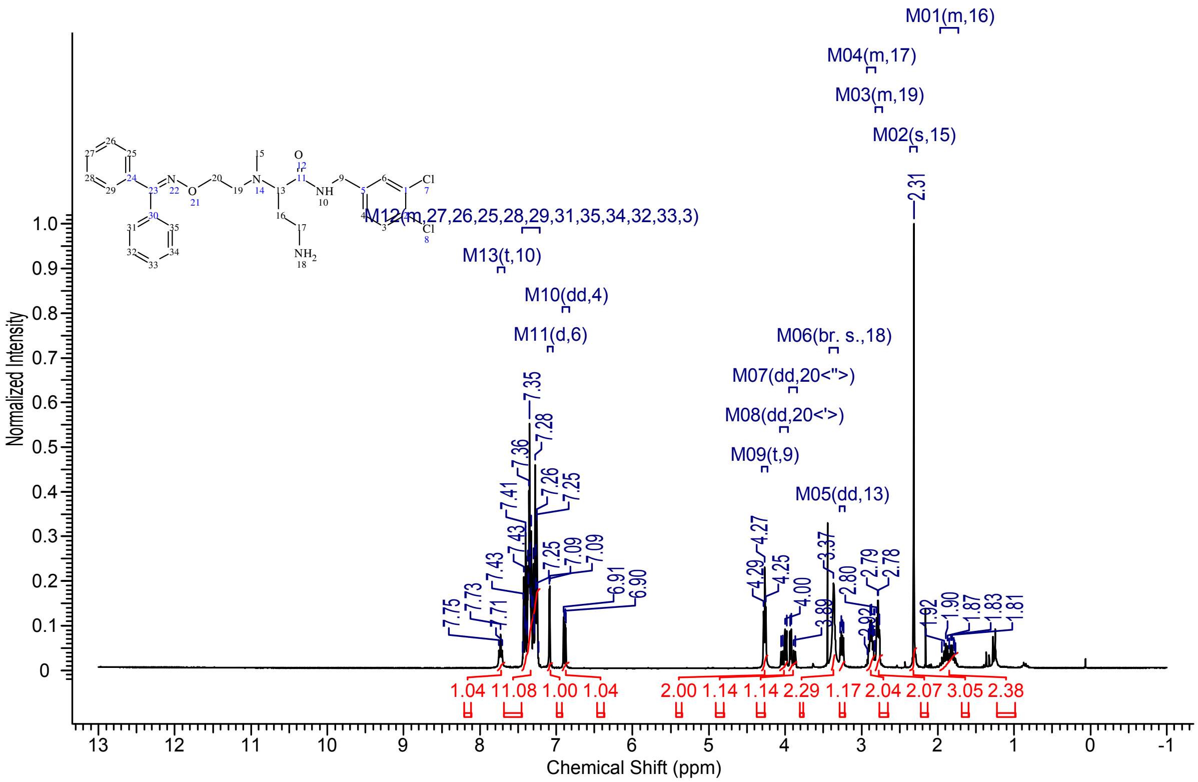

^{13}C NMR spectrum of 4-Amino-*N*-[(3,4-dichlorophenyl)methyl]-2-[(2-{[(diphenylmethylidene)amino]-oxy}ethyl)(methyl)amino] butanamide (**50b**)

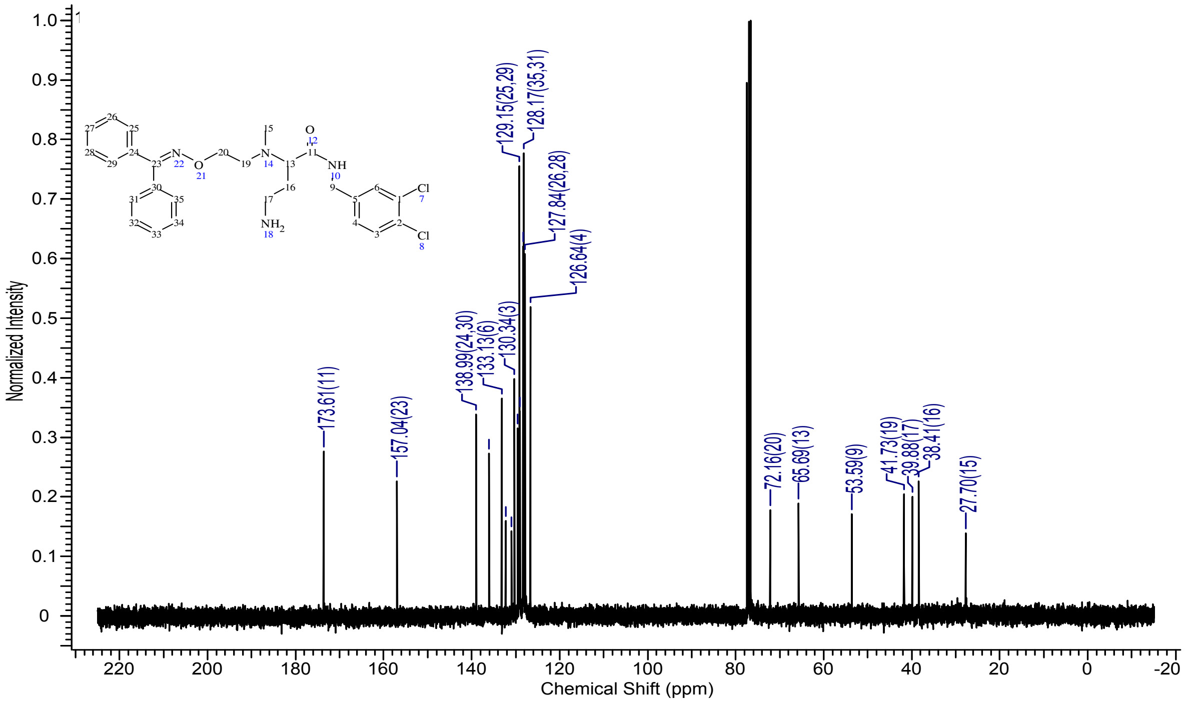

^{1}H NMR spectrum of *N*-[(2-Chlorophenyl)methyl]-2-[(2-{[(diphenylmethylidene)amino]-oxy}ethyl)(methyl)amino]-4-acetamidobutanamide (**51a**)

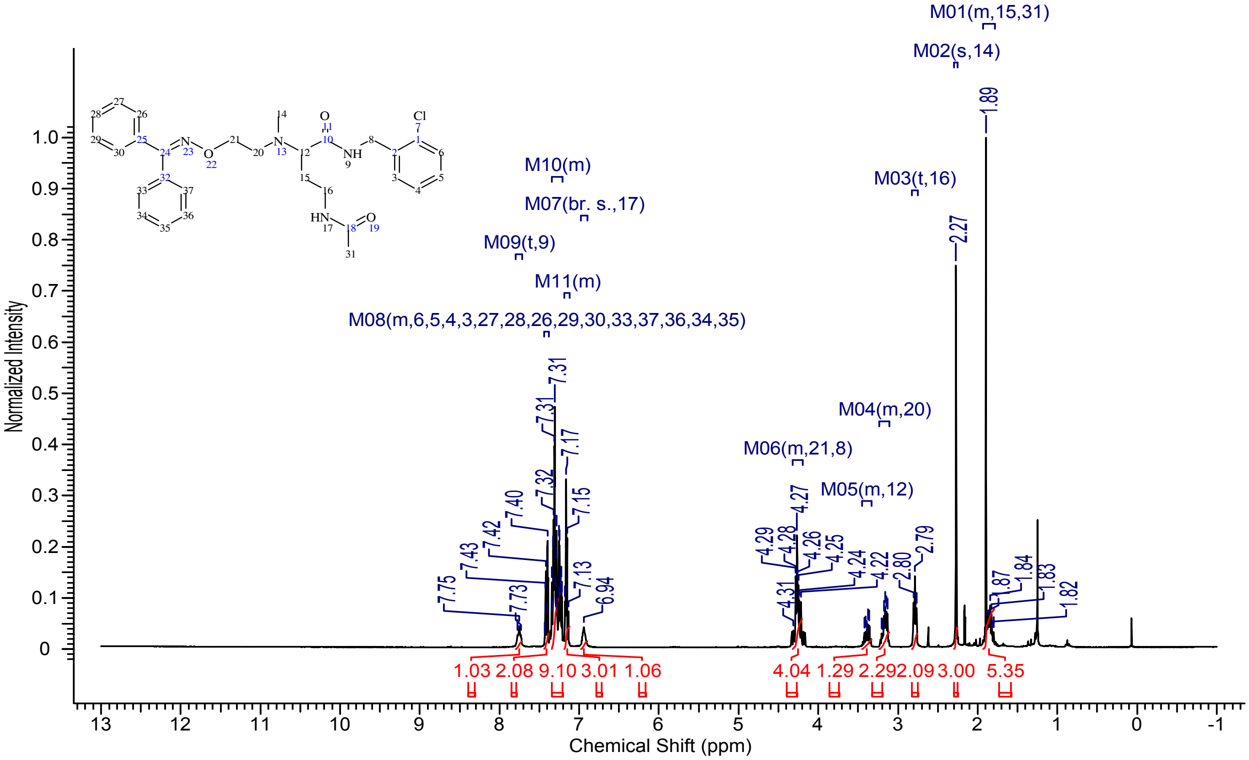

^{13}C NMR spectrum of *N*-[(2-Chlorophenyl)methyl]-2-[(2-{[(diphenylmethylidene)amino]-oxy}ethyl)(methyl)amino]-4-acetamidobutanamide (**51a**)

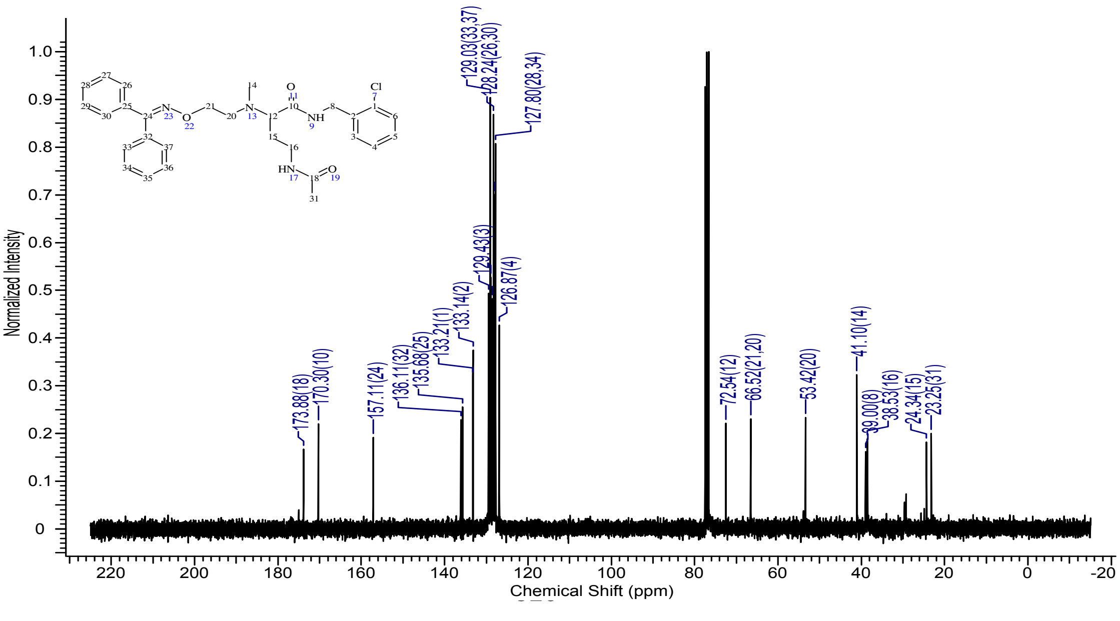

^{1}H NMR spectrum of *N*-Benzyl-2-{4-[bis(3-methylthiophen-2-yl)methylidene]piperidin-1-yl}-3-hydroxypropanamide (**54a**)

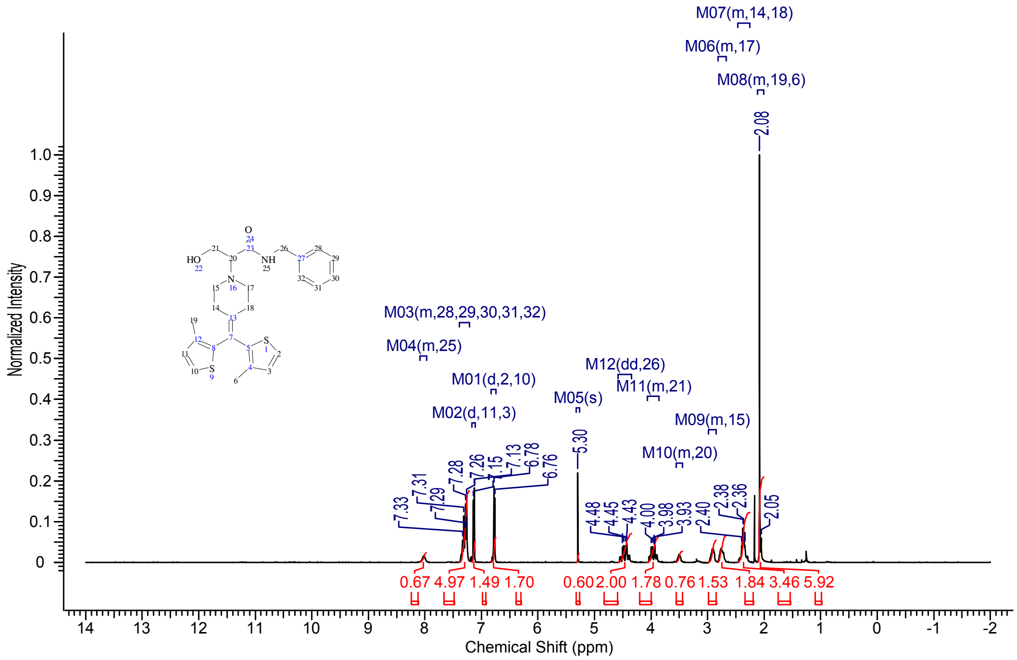

^{13}C NMR spectrum of *N*-Benzyl-2-{4-[bis(3-methylthiophen-2-yl)methylidene]piperidin-1-yl}-3-hydroxypropanamide (**54a**)

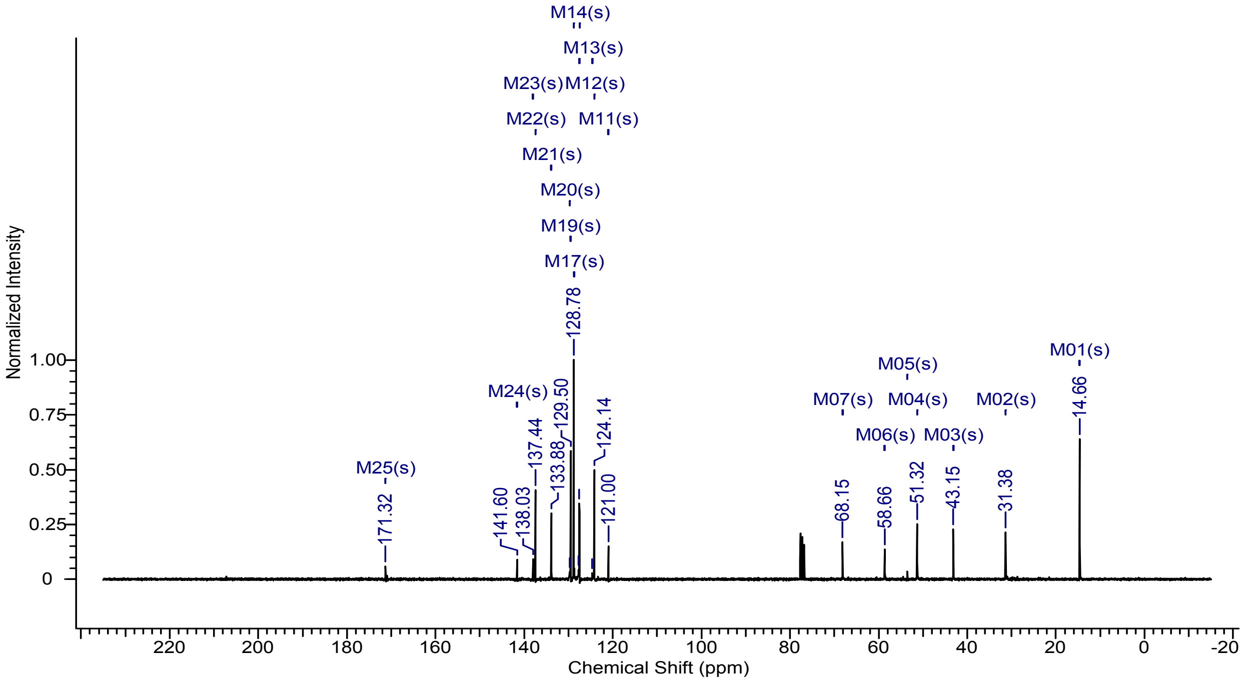

^{1}H NMR spectrum of 2-{4-[Bis(3-methylthiophen-2-yl)methylidene]piperidin-1-yl}-*N*-[(2-chlorophenyl)methyl]-3-hydroxypropanamide (**54b**)

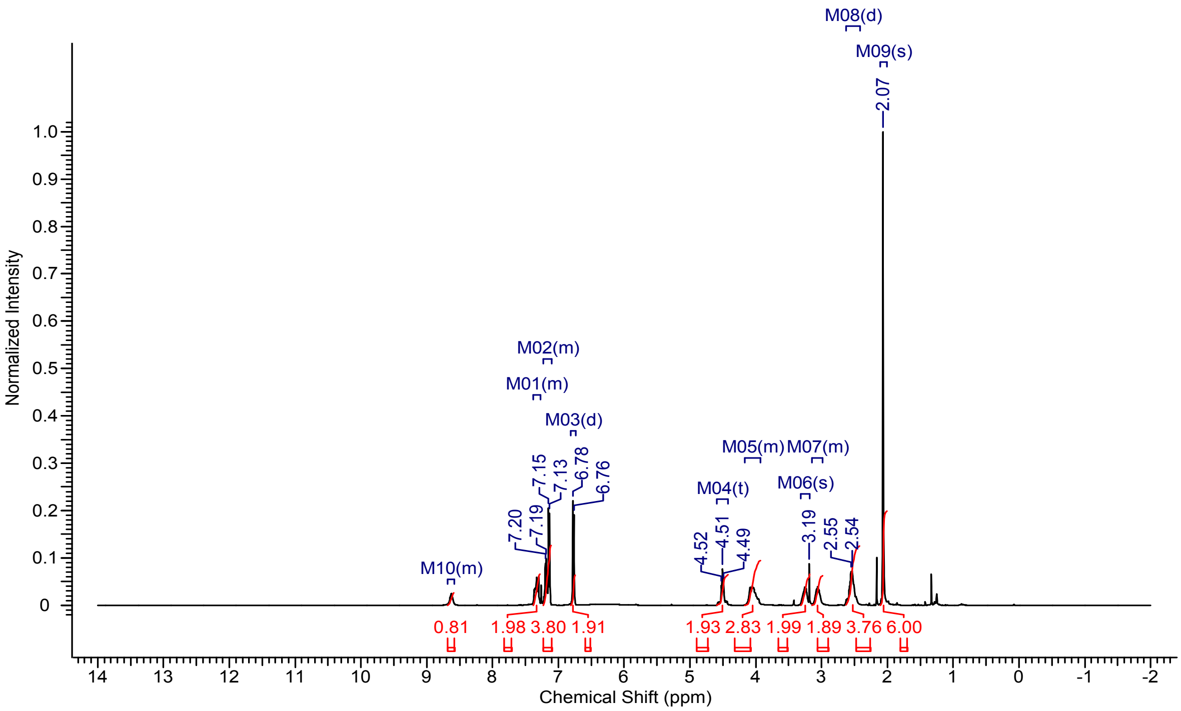

^{13}C NMR spectrum of 2-{4-[Bis(3-methylthiophen-2-yl)methylidene]piperidin-1-yl}-*N*-[(2-chlorophenyl)methyl]-3-hydroxypropanamide (**54b**)

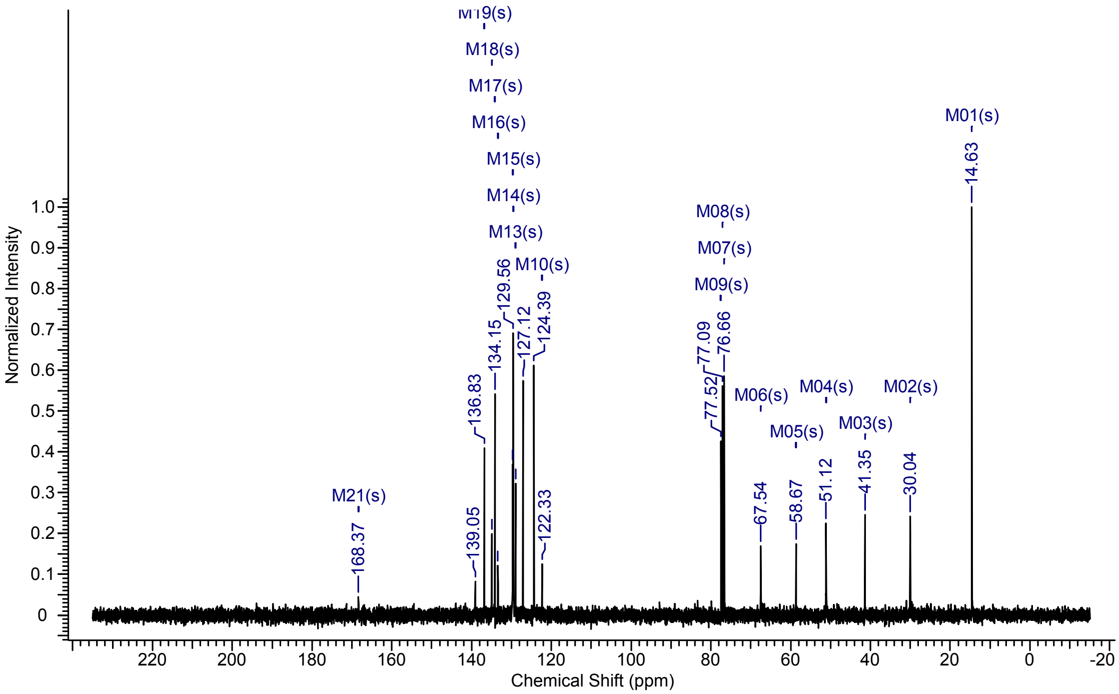

^{1}H NMR spectrum of 2-{4-[Bis(3-methylthiophen-2-yl)methylidene]piperidin-1-yl}-*N*-[(4-fluorophenyl)methyl]-3-hydroxypropanamide (**54c**)

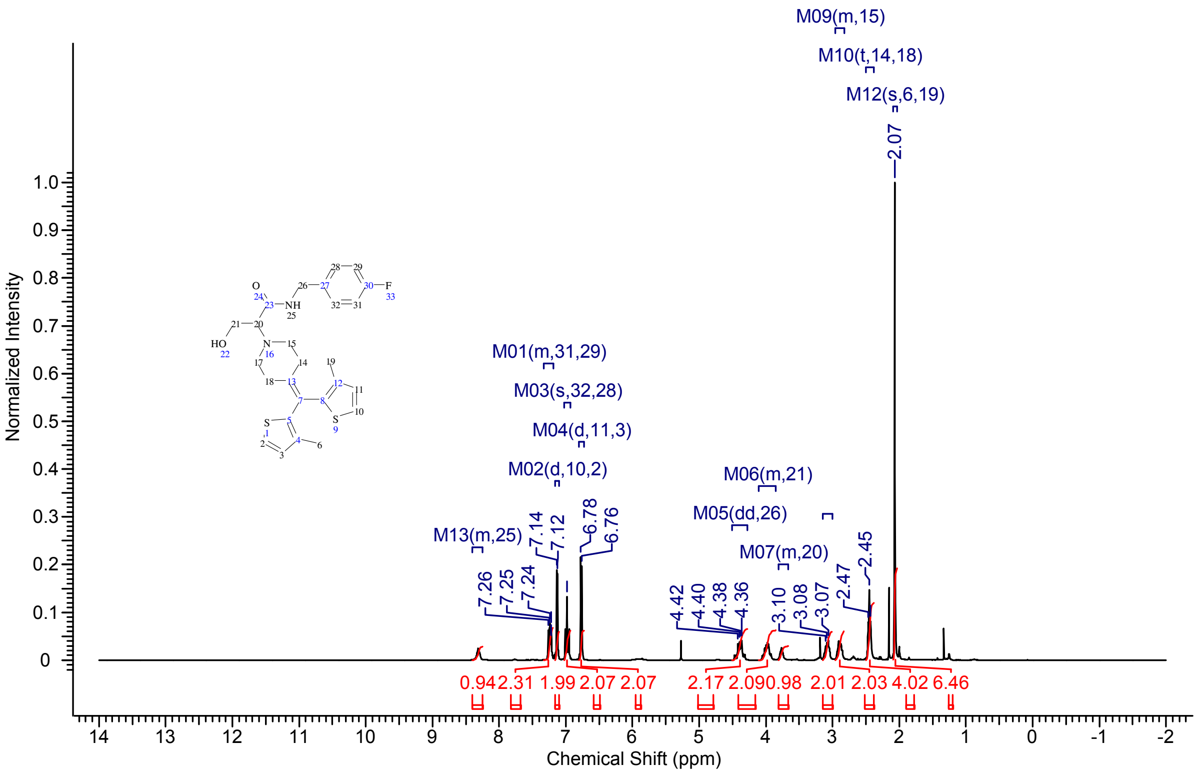

^{13}C NMR spectrum of 2-{4-[Bis(3-methylthiophen-2-yl)methylidene]piperidin-1-yl}-*N*-[(4-fluorophenyl)methyl]-3-hydroxypropanamide (**54c**)

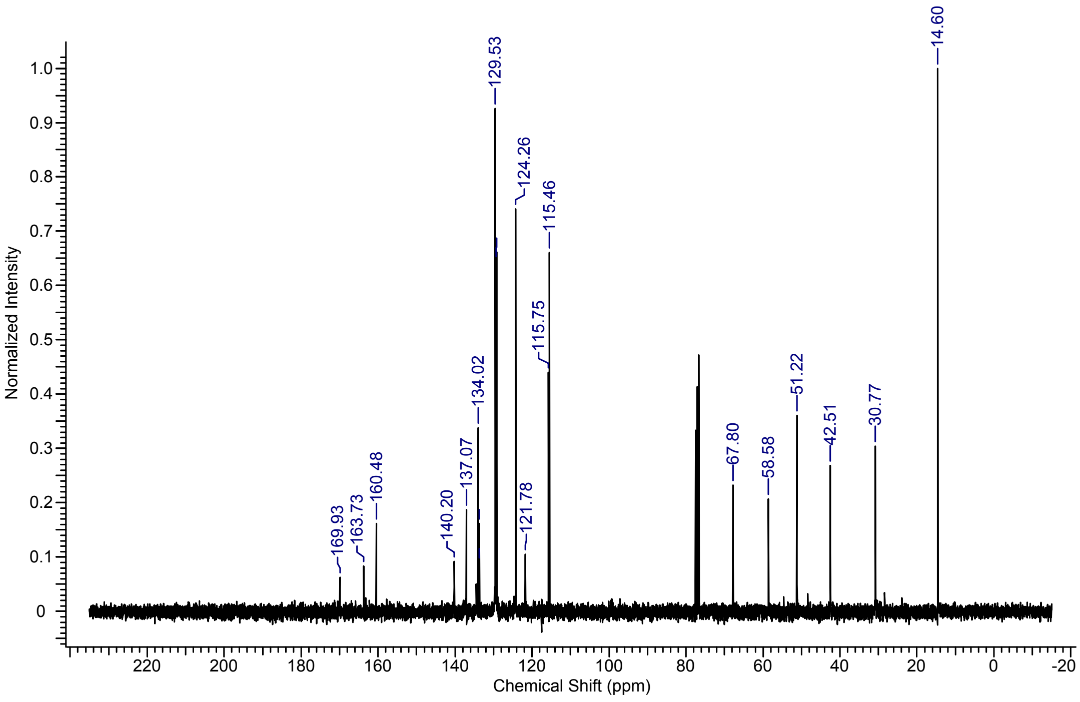

^{1}H NMR spectrum of *N*-Benzyl-2-((3-(10,11-dihydro-5*H*-dibenzo[a,d][7]annulen-5-ylidene)propyl)(methyl)amino)-3-hydroxypropanamide (**56a**)

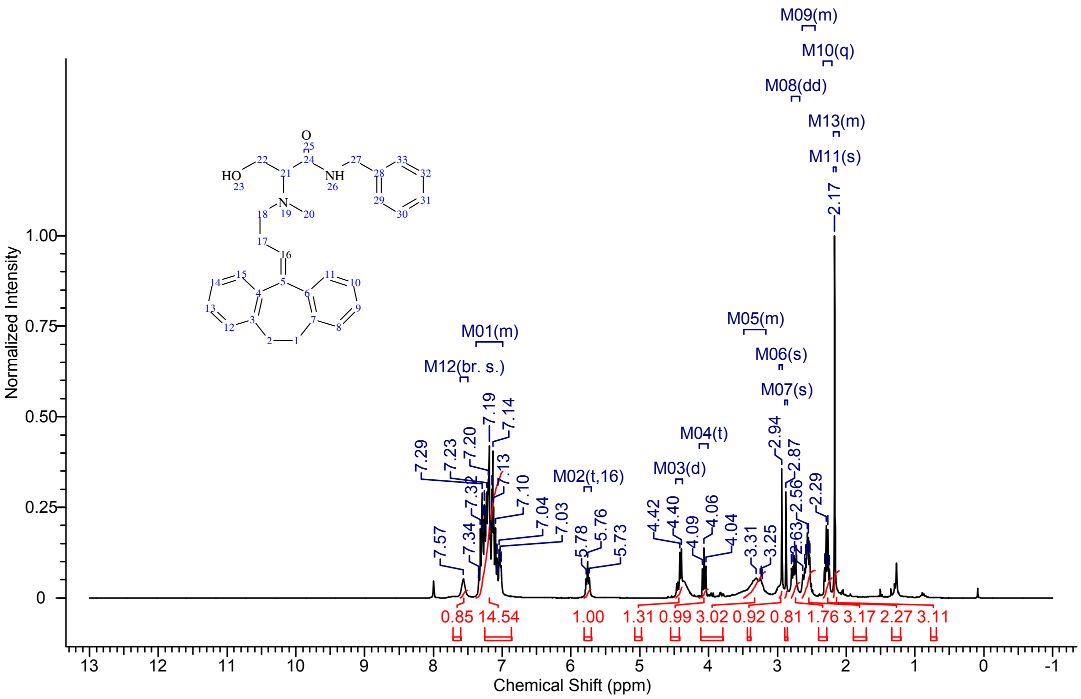

^{13}C NMR spectrum of *N*-Benzyl-2-((3-(10,11-dihydro-5*H*-dibenzo[a,d][7]annulen-5-ylidene)propyl)(methyl)amino)-3-hydroxypropanamide (**56a**)

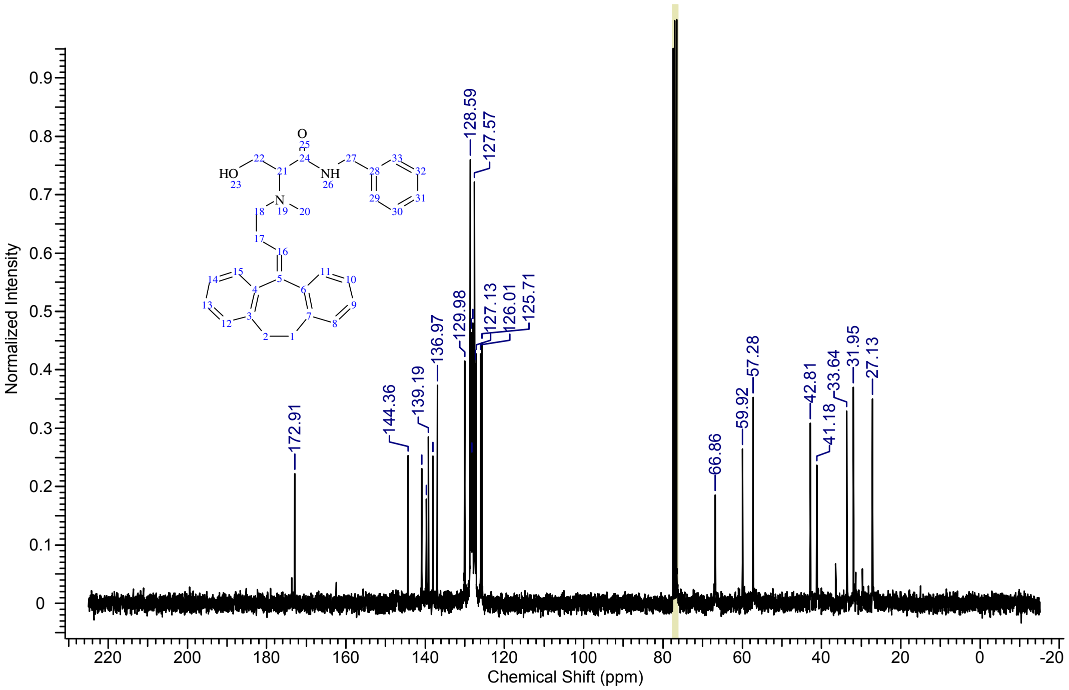

^{1}H NMR spectrum of *N*-Benzyl-2-(4-(10,11-dihydro-5*H*-dibenzo[a,d][7]annulen-5-ylidene)piperidin-1-yl)-3-hydroxypropanamide (**58**)

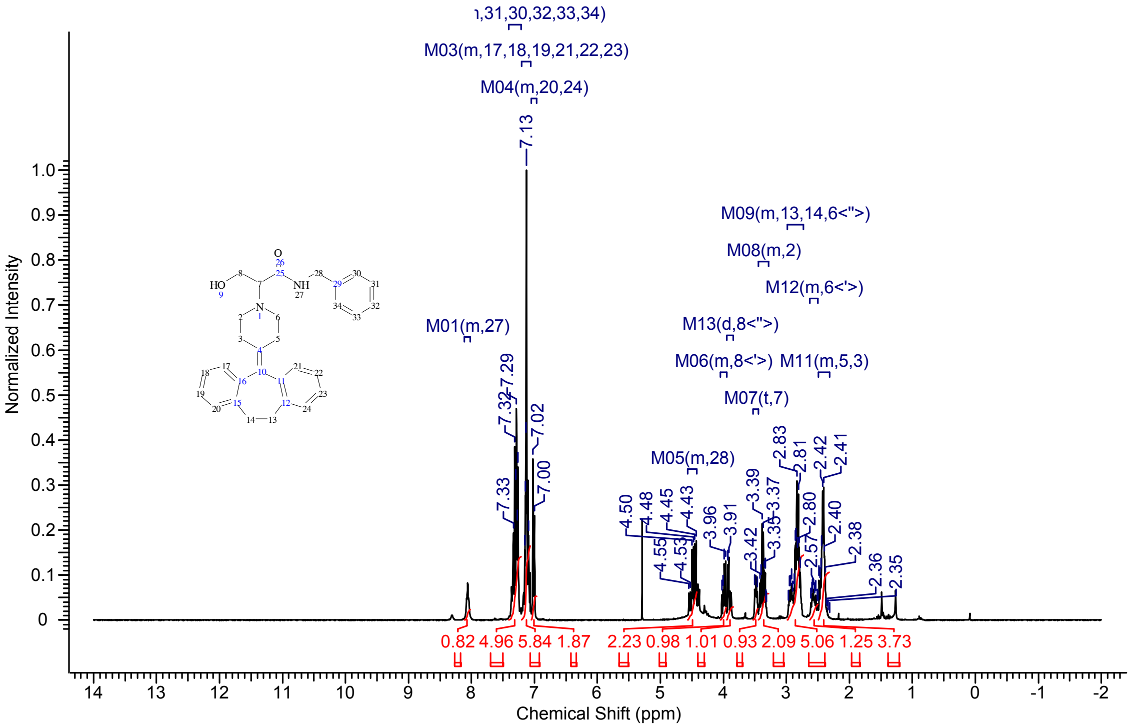

^{13}C NMR spectrum of (2-Chlorobenzyl)-2-((2-(((diphenylmethylene)amino)oxy)ethyl)(methyl)amino)-3-hydroxypropanamide (**59b**)

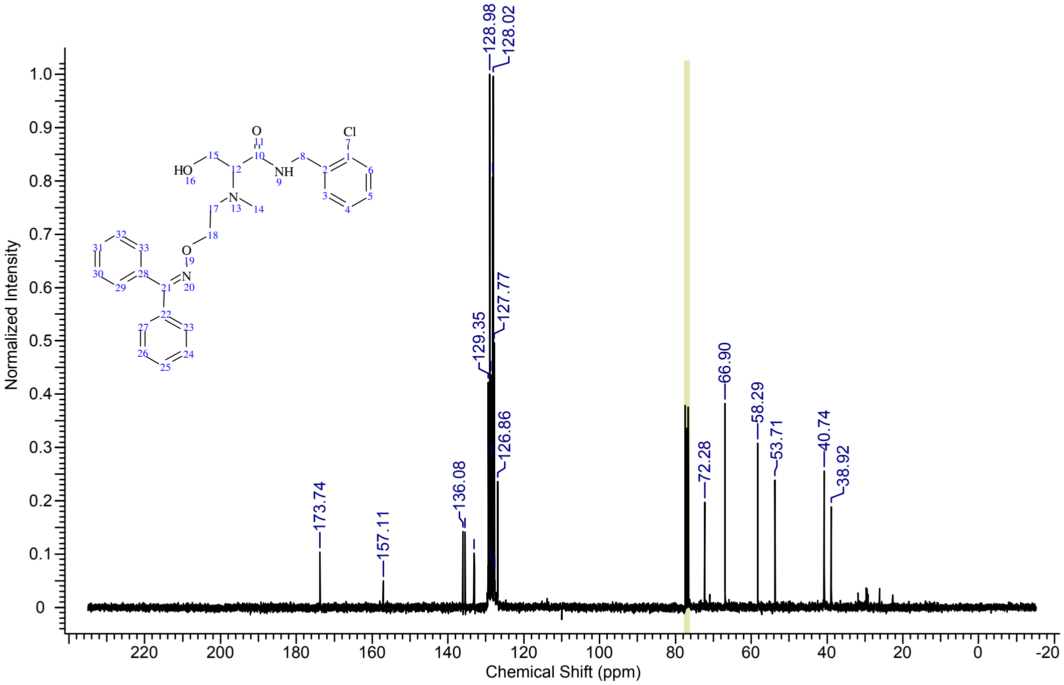

^{1}H NMR spectrum of *N*-Benzyl-2-((4,4-bis(3-methylthiophen-2-yl)but-3-en-1-yl)(methyl)amino)propanamide (**67**)

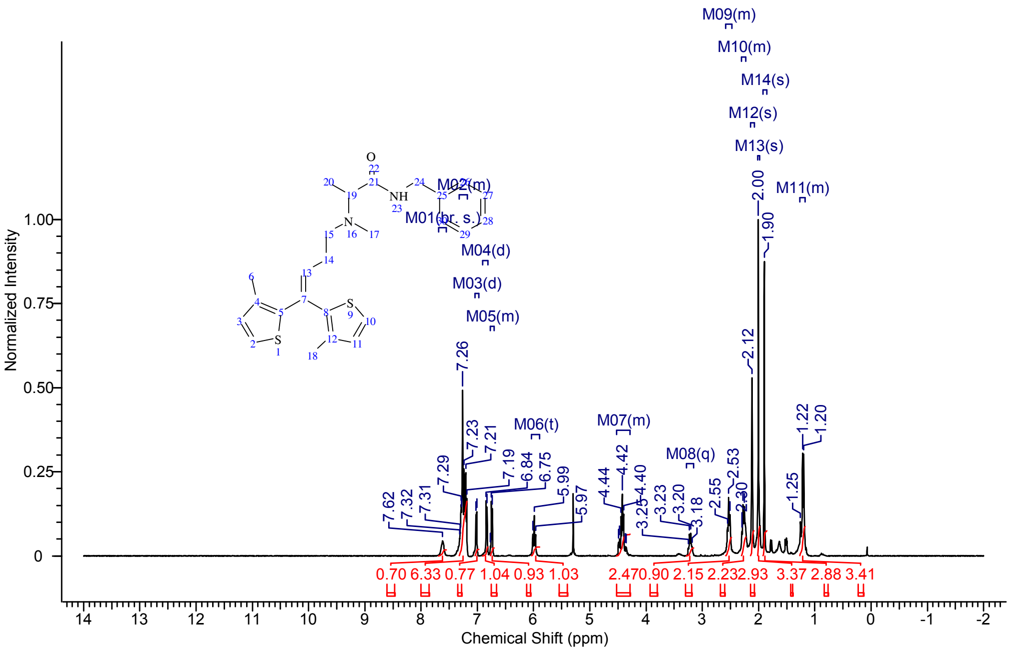

^{13}C NMR spectrum of *N*-Benzyl-2-((4,4-bis(3-methylthiophen-2-yl)but-3-en-1-yl)(methyl)amino)propanamide (**67**)

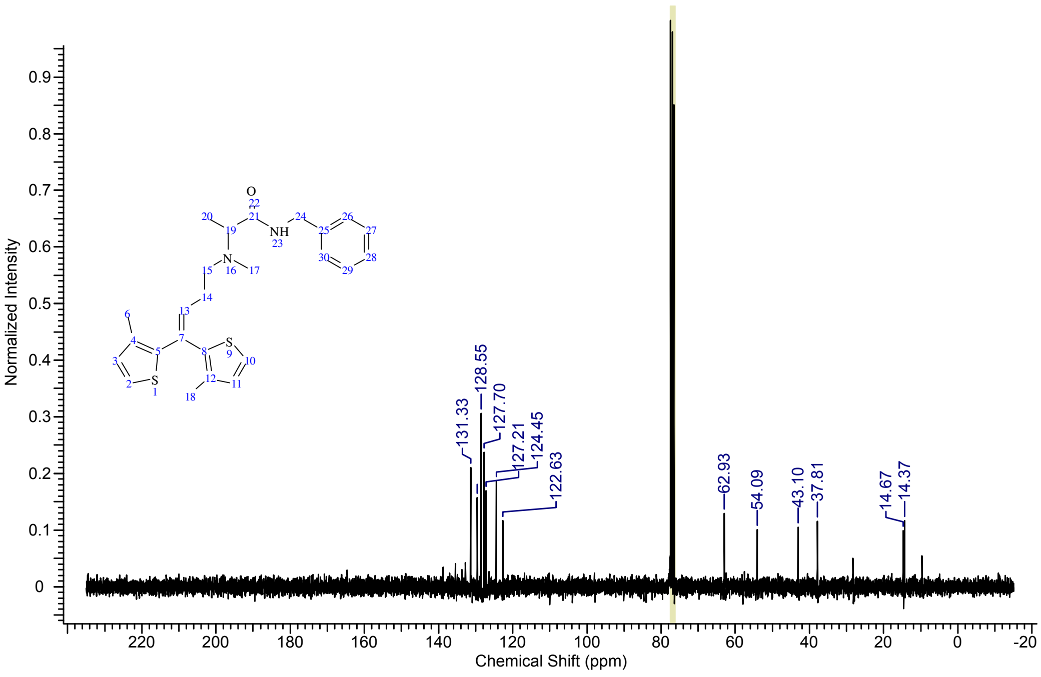

^{1}H NMR spectrum of ethyl 2-((4,4-bis(3-methylthiophen-2-yl)but-3-en-1-yl)(methyl)amino)propanoate (**73**)

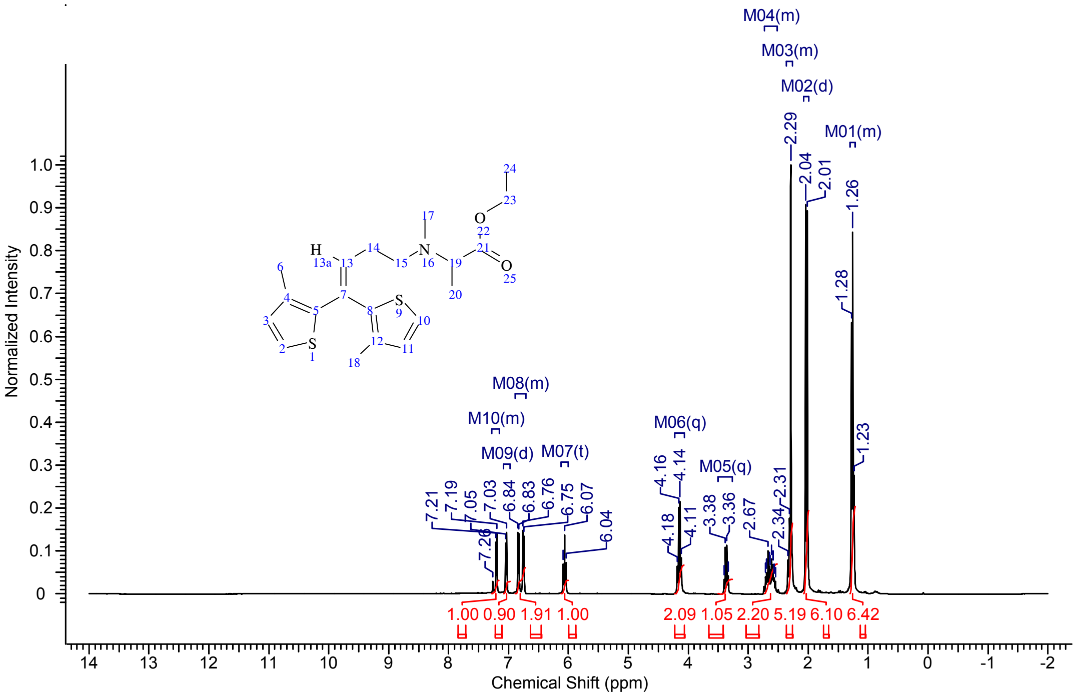

^{13}C NMR spectrum of ethyl 2-((4,4-bis(3-methylthiophen-2-yl)but-3-en-1-yl)(methyl)amino)propanoate (**73**)

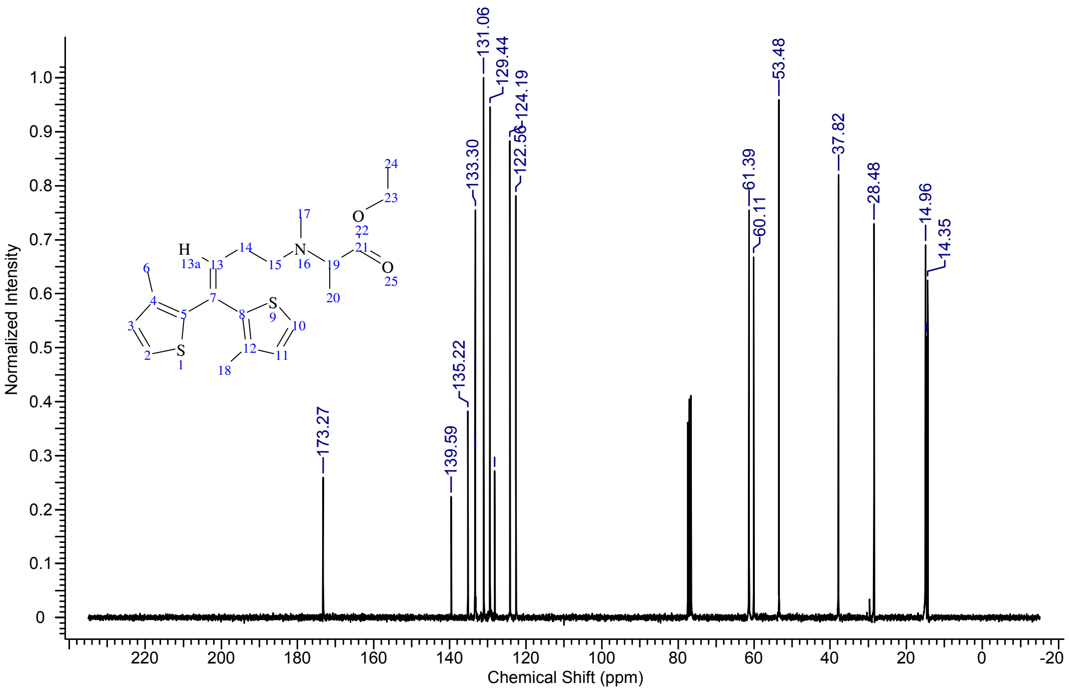

4. REFERENCE

[1] J. Lee, S.U. Kang, J.O. Lim, H.K. Choi, M.K. Jin, A. Toth, L. V. Pearce, R. Tran, Y. Wang, T. Szabo, P.M. Blumberg, N-[4-(Methylsulfonylamino)benzyl]thiourea analogues as vanilloid receptor antagonists: Analysis of structure-activity relationships for the “C-Region,” Bioorganic Med. Chem. 12 (2004) 371–385. https://doi.org/10.1016/j.bmc.2003.10.047.

[2] G. Chen, H. Xia, Y. Cai, D. Ma, J. Yuan, C. Yuan, Synthesis and SAR study of diphenylbutylpiperidines as cell autophagy inducers, Bioorganic Med. Chem. Lett. 21 (2011) 234–239. https://doi.org/10.1016/j.bmcl.2010.11.029.

[3] D. BOYLE, Craig, J. GREENLEE, William, S. CHACKALAMANNIL, Muscarine Antagonists WO 03/091220 A1, 2003.

[4] K. Stach, DE1203253, 1965.

[5] P. Zareba, K. Sałat, G.C. Hoefner, K. Łątka, M. Bajda, G. Latacz, K. Kotniewicz, A. Rapacz, A. Podkowa, M. Maj, K. Jóźwiak, B. Filipek, K.T. Wanner, B. Malawska, K. Kulig, Development of tricyclic N -benzyl-4-hydroxybutanamide derivatives as inhibitors of GABA transporters mGAT1-4 with anticonvulsant , antinociceptive , and antidepressant activity, Eur. J. Med. Chem. 221 (2021) 113512. https://doi.org/10.1016/j.ejmech.2021.113512.

[6] P.W. Jackson, Process for the preparation of 10,11-dihydro-5H-dibenzo[a,d]cyclohept-5-enes and derivatives thereof. WO 98/38166 A1, 1998.

[7] D.B. Kastrinsky, J. Sangodkar, N. Zaware, S. Izadmehr, N.S. Dhawan, G. Narla, M. Ohlmeyer, Reengineered tricyclic anti-cancer agents, Bioorg. Med. Chem. 23 (2015) 6528–6534. https://doi.org/10.1016/j.bmc.2015.07.007.

[8] P.P. Kung, R. Bharadwaj, A.S. Fraser, D.R. Cook, A.M. Kawasaki, P.D. Cook, Solution-phase synthesis of novel linear oxyamine combinatorial libraries with antibacterial activity, J. Org. Chem. 63 (1998) 1846–1852. https://doi.org/10.1021/jo971655u.

[9] X. Li, J. Hou, C. Wang, X. Liu, H. He, P. Xu, Z. Yang, Z. Chen, Y. Wu, L. Zhang, Synthesis and Biological Evaluation of RGD-Conjugated MEK1/2 Kinase Inhibitors for Integrin-Targeted Cancer Therapy, Molecules. 18 (2013) 13957–13978. https://doi.org/10.3390/molecules181113957.

[10] K.E. Andersen, C. Braestrup, F.C. Grønwald, A.S. Jørgensen, E.B. Nielsen, U. Sonnewald, P.O. Sørensen, P.D. Suzdak, L.J. Knutsen, The synthesis of novel GABA uptake inhibitors. 1.

Elucidation of the structure-activity studies leading to the choice of (R)-1-[4,4-bis(3-methyl-2-thienyl)-3-butenyl]-3-piperidinecarboxylic acid (tiagabine) as an anticonvulsant drug candidate., J. Med. Chem. 36 (1993) 1716–1725. https://doi.org/10.1021/jm00064a005.

[11] R.P. Clausen, E.K. Moltzen, J. Perregaard, S.M. Lenz, C. Sanchez, E. Falch, B. Frølund, T. Bolvig, A. Sarup, O.M. Larsson, A. Schousboe, P. Krogsgaard-Larsen, Selective inhibitors of GABA uptake: Synthesis and molecular pharmacology of 4-N-methylamino-4,5,6,7-tetrahydrobenzo[d]isoxazol-3-ol analogues, Bioorganic Med. Chem. 13 (2005) 895–908. https://doi.org/10.1016/j.bmc.2004.10.029.

[12] S.B. Vogensen, L. Jørgensen, K.K. Madsen, N. Borkar, P. Wellendorph, J. Skovgaard-Petersen, A. Schousboe, H.S. White, P. Krogsgaard-Larsen, R.P. Clausen, Selective mGAT2 (BGT-1) GABA uptake inhibitors: Design, synthesis, and pharmacological characterization, J. Med. Chem. 56 (2013) 2160–2164. https://doi.org/10.1021/jm301872x.

[13] S. Song, S.F. Zhu, L.Y. Pu, Q.L. Zhou, Iridium-catalyzed enantioselective hydrogenation of unsaturated heterocyclic acids, Angew. Chemie - Int. Ed. 52 (2013) 6072–6075. https://doi.org/10.1002/anie.201301341.
